# Use of hormone replacement therapy and risk of breast cancer: nested case-control studies using the QResearch and CPRD databases

**DOI:** 10.1136/bmj.m3873

**Published:** 2020-10-28

**Authors:** Yana Vinogradova, Carol Coupland, Julia Hippisley-Cox

**Affiliations:** 1 Division of Primary Care, University Park, University of Nottingham, Nottingham NG2 7RD, UK; 2 Nuffield Department of Primary Care Health Sciences, University of Oxford, Oxford, UK

## Abstract

**Objective:**

To assess the risks of breast cancer associated with different types and durations of hormone replacement therapy (HRT).

**Design:**

Two nested case-control studies.

**Setting:**

UK general practices contributing to QResearch or Clinical Practice Research Datalink (CPRD), linked to hospital, mortality, social deprivation, and cancer registry (QResearch only) data.

**Participants:**

98 611 women aged 50-79 with a primary diagnosis of breast cancer between 1998 and 2018, matched by age, general practice, and index date to 457 498 female controls.

**Main outcome measures:**

Breast cancer diagnosis from general practice, mortality, hospital, or cancer registry records. Odds ratios for HRT types, adjusted for personal characteristics, smoking status, alcohol consumption, comorbidities, family history, and other prescribed drugs. Separate results from QResearch or CPRD were combined.

**Results:**

Overall, 33 703 (34%) women with a diagnosis of breast cancer and 134 391 (31%) controls had used HRT prior to one year before the index date. Compared with never use, in recent users (<5 years) with long term use (≥5 years), oestrogen only therapy and combined oestrogen and progestogen therapy were both associated with increased risks of breast cancer (adjusted odds ratio 1.15 (95% confidence interval 1.09 to 1.21) and 1.79 (1.73 to 1.85), respectively). For combined progestogens, the increased risk was highest for norethisterone (1.88, 1.79 to 1.99) and lowest for dydrogesterone (1.24, 1.03 to 1.48). Past long term use of oestrogen only therapy and past short term (<5 years) use of oestrogen-progestogen were not associated with increased risk. The risk associated with past long term oestrogen-progestogen use, however, remained increased (1.16, 1.11 to 1.21). In recent oestrogen only users, between three (in younger women) and eight (in older women) extra cases per 10 000 women years would be expected, and in oestrogen-progestogen users between nine and 36 extra cases per 10 000 women years. For past oestrogen-progestogen users, the results would suggest between two and eight extra cases per 10 000 women years.****

**Conclusion:**

This study has produced new generalisable estimates of the increased risks of breast cancer associated with use of different hormone replacement preparations in the UK. The levels of risks varied between types of HRT, with higher risks for combined treatments and for longer duration of use.

## Introduction

Hormone replacement therapy (HRT) (also known as hormone therapy (HT) or menopausal hormonal therapy (MHT)) is prescribed to relieve the symptoms of menopause, which can be life changing. HRT is used by millions of women, sometimes over extended periods. A range of hormone combinations are available, each with different efficacy and side effects. HRT can bring several improvements to quality of life, and it can prevent osteoporosis. Concerns about adverse effects, particularly the increased risk of breast cancer associated with HRT,^1^ has, however, resulted in a substantial decrease in HRT use over the past 17 years.^2^ Breast cancer is the most common cancer in women, with more than 55 000 women in the UK affected each year,^3^ so different drug use scenarios might result in substantial differences in the number of women who develop breast cancer, even though the risk differences between hormones might seem relatively small. Current clinical guidelines recommend use of HRT for no longer than five years and have signalled that more information is needed about the risks of breast cancer associated with different types of HRT.^4^ ^5^

Randomised trials using enrolled participants are now impractical to investigate the risks of breast cancer associated with HRT because of the numbers required and the length of follow-up. Trials would also be difficult to justify ethically, given the known harms that are associated with some types of HRT. Earlier trials have been limited, focusing on specific age groups possibly unrepresentative of women likely to request HRT or selecting specific types of HRT^6^ (the largest being the Women’s Health Initiative trial),^7^ or, because the design failed to distinguish between treatment types, looking only at overall effect or association.^8^ ^9^ ^10^ Observational studies are more feasible but require access to large datasets covering lengthy time periods; so far only the Million Women Study has approached the requisite power.^10^ A recent meta-analysis published after our study commenced, pooled information from 24 prospective observational studies to provide more comprehensive data on the details of exposure and breast cancer risks for the most commonly prescribed oestrogens and progestogens.^11^ This meta-analysis reported that the risk of breast cancer is increased for both oestrogen only and oestrogen-progestogen current users, with, respectively, a 17% and 60% increase for 1-4 years of use and a 33% and 108% increase for 5-14 years of use. The results also showed a remaining increased risk even after discontinuation of HRT. As with many meta-analyses, however, the included studies were conducted in different settings, had different selection criteria, and had different definitions of exposure, so the data and original study designs were heterogeneous. The study provided information for the most commonly used HRT preparations, albeit with notably smaller statistical power for dydrogesterone—a progestogen previously found to be associated with a low increased risk of breast cancer.^12^ At publication, the focus of publicity was the higher than expected associations with breast cancer risks than had been suggested by earlier trials. The Medicine and Healthcare products Regulatory Agency subsequently raised an HRT drug safety alert specific to breast cancer, but this has since been questioned as having caused “considerable anxiety,” particularly for women who might need HRT for reasons other than menopausal symptoms.^13^

Our study focused on exposure to all the commonly prescribed types of HRT in the UK over the past 20 years in a representative primary care population. We assessed the differences in risks associated with the individual component hormones used in HRT, including dydrogesterone. Our findings are based on prospectively collected electronic health records from the two largest UK primary care databases linked to secondary care data sources. We analysed these separately and then combined the results. In contrast with data and analytical designs used in studies included in the recent meta-analysis,^11^ our data were homogeneous, and the analytical approach was common. This has allowed us to gain a realistic picture of exposure in the UK to component hormones used in HRT, and the associations with increased breast cancer risk of specific treatments, providing consistently derived information for patients and doctors.

## Methods

### Study design

Full details for this study are available in the published protocol.^14^ To summarise, we undertook a nested case-control study using the two largest UK primary care databases, QResearch and Clinical Practice Research Datalink (CPRD) GOLD, and utilised linked data from Hospital Episode Statistics (HES), Office for National Statistics (ONS) mortality data, and (QResearch only) cancer registry data. We included all general practices that had contributed data for at least three years and from these we identified two open cohorts of women aged between 50 and 79 and registered with the general practice between 1 January 1998 and 31 December 2018. We excluded women with already diagnosed breast cancer or records of mastectomy at the cohort entry date, and, to ensure completeness, any with fewer than three years of medical records.

### Selection of cases and controls

Across both databases, we identified all cases between 1 January 1998 and 31 December 2018. From the QResearch database, we identified all cases of incident breast cancer using general practice, hospital admission, mortality, and cancer registry records. From CPRD, when practices were linked, we used general practice, hospital admission (up to 31 December 2017), and mortality data records (up to 13 February 2018) to identify cases, and, when not linked, general practice records only. Each case was matched to a maximum of five controls by year of birth and general practice using incidence density sampling.^15^ For each case in any data source, the date of the first breast cancer record became the index date for their matched controls. QResearch and CPRD GOLD use different computer systems to collect records from practices, and as patients can be registered with only one practice, there was no overlap of cases and controls.

### Exposure to HRT

We extracted prescription information for all oestrogens, progestogens, and tibolone from practice records. Symptoms indicative of developing breast cancer before diagnosis could have resulted in cessation of HRT. To minimise this source of possible protopathic bias, we excluded prescriptions issued in the year before the index date.^16^

Exposure to HRT was taken as the date from when a woman received her first prescription containing systemic oestrogen (oral, subcutaneous, or transdermal) indicated to treat menopausal symptoms. If a woman received no prescription that contained a progestogen after this date, she was classified as an oestrogen only therapy user. If a woman received any prescription that contained a progestogen, she was classified as a combined therapy user. We also included topical oestrogen preparations (vaginal pessaries or cream) and tibolone, because both are commonly prescribed to menopausal women.

A large proportion of women switched between different combinations of oestrogens and progestogens, so we analysed each hormonal preparation as a separate exposure. For oestrogen only users, we distinguished between types, doses, and application method, whereas for combined therapy users we analysed combinations of any oestrogen, concentrating on progestogen type and application method. If combined therapy users had also used oestrogen only therapy, we analysed the women as oestrogen-progestogen users but adjusted the combined exposure results to account for periods of oestrogen only treatment. For all treatments, the reference category was no exposure (never users) to HRT.

At the time of our study, two types of oestrogen (conjugated equine oestrogen and estradiol) and four types of progestogen (norethisterone acetate, levonorgestrel, medroxyprogesterone, and dydrogesterone) were commonly prescribed in the UK and were included in our analyses. Of these, sufficient data were available for estradiol, estradiol-norethisterone, and oestrogen-levonorgestrel to facilitate separate analysis of application methods—oral, transdermal, or injection, and (for levonorgestrel) intrauterine. We investigated two daily dosage levels of oestrogen: low (0.625 mg/day or less for oral conjugated equine oestrogen, 1 mg/day or less for oral estradiol, and 50 mg or less for transdermal estradiol) and high (all other dosage levels). Median dosages for each oestrogen and for each woman were also calculated and analysed.

We have not specified the type of oestrogen for combinations with progestogens, but in our data conjugated equine oestrogen was by far the most commonly prescribed drug in combination with medroxyprogesterone (only 16% of prescriptions included estradiol) and levonorgestrel (only 5% included estradiol). Estradiol was the only oestrogen prescribed in combination with norethisterone and dydrogesterone.

Our data showed that HRT prescriptions were frequently issued for three months, so we assessed the durations of use by summing the lengths of prescriptions in days, including gaps of fewer than 90 days between prescriptions. Most (79%) repeated prescriptions were, however, issued within 30 days. We then categorised durations of use as never (0), less than 1 year, 1-2 years (≥1 and <3), 3-4 years (≥3 and <5), 5-9 years (≥5 and <10), and 10 years or more. Excluding prescriptions in the past year, the gap between the end of the last prescription and the index date was categorised as 1-2 years (>1 and <2), 2-4 years (≥2 and <4), 5-9 years (≥5 and <10), and 10 years or more.

Because some women discontinued HRT more than a year before the index date, and associated breast cancer risks might have diminished noticeably, we investigated two recency related exposures: recent, if the women had a prescription more than one year and less than five years before the index date (this includes current users of HRT at one year before the index date), and past, if their last prescription ended before that period (≥5 years before). Using these, we analysed different durations of exposures in relation to the recency of the last prescription.

### Confounders

Analyses were all adjusted by the same factors—those that might have affected a doctor’s prescribing decision for HRT or might have affected a woman’s decision to take HRT or are associated with an increased breast cancer risk.^3^ ^11^ ^14^ The data for confounders were derived from practice or hospital records, and data for drugs were from practice records only. To minimise protopathic bias, records of confounders had to be from at least a year before the index date. Confounders included lifestyle factors (smoking status, alcohol consumption, body mass index (BMI), and Townsend fifth as a measure of deprivation (in QResearch only)), self-assigned ethnicity (based on practice and hospital data), family history of cancers and osteoporosis, history of other cancers, records of early and late menopause, oophorectomy or hysterectomy, uptake of mammography or scanning, menopausal symptoms, comorbidities, and use, or when possible, duration of use of other drugs. Comorbidities included benign breast disease, diabetes, and bipolar disorder or schizophrenia.^14^ Other drugs included combined and progestogen only contraceptive drugs, aspirin, non-steroidal anti-inflammatory drugs, tamoxifen, and raloxifene. When numbers permitted, we categorised duration of use of other drugs up to one year before the index date as never, less than 1 year, 1-2 years, 3-4 years, and 5 years or more. Early menopause was estimated from records of menopausal symptoms or of oophorectomy or hysterectomy before age 45 years. Late menopause was considered if the first menopause related record was after 55 years for women older than 55 at the index date. For all other women we assumed onset of menopause was between age 50 and 55 years.

### Statistical analysis

As data from QResearch and CPRD cannot be pooled, for all analyses we processed extracted datasets in parallel as similarly as possible. To calculate associations between breast cancer risk and different exposures to HRT, we used conditional logistic regression to estimate odds ratios with 95% confidence intervals. A small proportion of women had missing values for BMI, smoking status, and alcohol consumption, which we assumed to be missing at random. We imputed these separately for each dataset using chained equations over 10 imputed datasets, where the imputation model included all listed confounders, exposures, and case-control status indicators, and we combined the odds ratios obtained from the imputed datasets using Rubin’s rule.^17^

We considered duration of exposure both in the form of defined categories of exposure and as a continuous variable. For ease of comparability with other studies and to simplify interpretation, our main results are presented using defined categories of exposure, with duration of HRT expressed in years. We used a meta-analytical technique to combine the obtained odds ratios from the separate analyses run on each database.^18^ A fixed effect model with inverse variance weights was used for the main analysis and a random effect model as a sensitivity analysis. In the main tables and text we only include the combined results; the separate results for QResearch and CPRD are in the supplementary tables.

To model exposures as continuous variables, we ran separate analyses on each database using fractional polynomials to explore non-linear risk associations for durations of exposure, measured in days.^19^ This was done for both recent and past exposures to all the types of HRT under investigation. Variables found to have non-linear associations were then transformed into the suggested powers, the separate analyses were rerun, and the resulting coefficients and standard errors were combined.

### Additional and sensitivity analyses

To assess possible age related differences in risks associated with exposures to hormone, we performed additional analyses for different age categories at the index date: 50-59 years, 60-69 years, and 70-79 years. We ran another subgroup analysis for women in three different BMI groups: less than 25, 25 up to 30, and 30 or more. In this analysis, we included only controls in the same body mass category as their matched case.

For the main analysis, we considered women to have recently used HRT if they had a prescription between one and five years before the index date. The risk associated with HRT has been found to decrease rapidly after discontinuation,^20^ so we needed a measure showing excess of risk for the most recently exposed women. To assess this, we repeated the analysis, defining recent use as exposure between one and two years before the index date.

It is possible that some women were classified as never exposed only because they were not registered with the practice at the time when they had used HRT. Although any systematic difference between cases and controls is unlikely, we addressed this possible misclassification of exposure by repeating the analysis in a subgroup of women with at least 10 years of medical records. Another sensitivity analysis dealt with unknown adherence to HRT, because it is possible that some women with apparent gaps between prescriptions had in fact spread their HRT supply over longer periods. In this analysis, we defined duration of HRT use as the period between the first HRT prescription and the last one prior to one year before the index date.

The main analysis was run on women aged 50 to 79, which may include some premenopausal and perimenopausal women who have a higher risk of breast cancer.^21^ To deal with this and provide comparability of our results with those of a meta-analysis,^11^ we also ran an additional analysis restricting our sample to women aged 55 to 79.

To check our assumption of missing at random for some confounders, we compared patterns of missingness in exposed and non-exposed women and repeated the analyses, including only cases and controls with recorded values. In the final sensitivity analysis, we dealt with problems that might have arisen from the different levels of linkage in QResearch and CPRD. All QResearch practices were linked to deprivation, hospital, mortality, and cancer registry data, so cases could be identified using all sources of data. For CPRD, however, only 60% of practices were linked to deprivation, hospital, and mortality data, so to include data from all usable practices, we were limited to identifying cases from all available data. To assess the possible effect on our results, we ran an analysis using only fully linked CPRD practices.

To estimate the excess of breast cancer cases associated with different HRT exposures we calculated the incidence rate in the unexposed female population for different age categories (50-59, 60-69, and 70-79) using the underlying cohort from CPRD. The rate in the exposed population was derived by multiplying the baseline rate by relevant odds ratios obtained from the combined analysis.

We used Stata v16 for all analyses. A 1% level of statistical significance was used to allow for multiple comparisons. To facilitate comparison with other studies, however, we present the results as odds ratios with 95% confidence intervals.

### Patient and public involvement

This epidemiological study investigated a research question recommended by a National Institute for Health and Care Excellence committee, which included lay members.^5^ It used routinely collected data and appropriate statistical techniques. The grant application process and the publication process of *The BMJ* both had lay involvement. No other lay people were involved in setting or extending the research question or the outcome measures in our study, nor were they involved in developing plans for the design or implementation of the study. However, to better understand motivations for starting HRT and possible adherence issues related to prescribed treatment, formal and informal conversations with some women taking HRT were also organised. In these, women generally reported high levels of adherence, regardless of whether they had sought treatment themselves or were recommended it by a doctor. Some of the women involved have also agreed to help further with interpretation and dissemination of the results through women’s menopausal forums.

## Results

**Fig 1:**
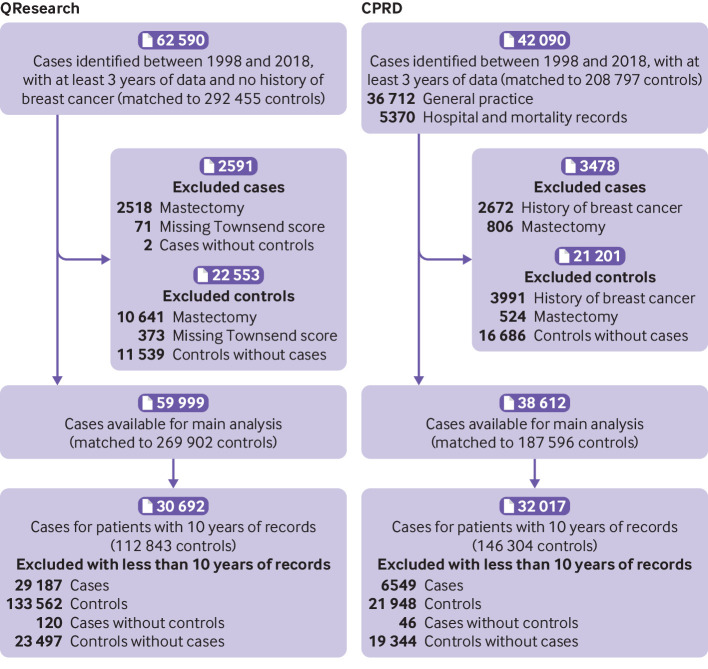
Flow chart of included cases and controls

Overall, 59 999 cases of breast cancer were identified in QResearch between 1 January 1998 and 31 July 2018, using general practice, hospital admissions, mortality, and cancer registry records. In total, 38 612 breast cancer cases were identified in CPRD between 1 January 1998 and 31 December 2018, using general practice records and, for linked practices, also using hospital admission records (until 31 December 2017) and mortality records (until 13 February 2018) (fig 1).

Table 1 shows the characteristics of the cases and matched controls from QResearch and CPRD. Cases were more likely than controls to be overweight or obese (53% *v* 50%), to be former smokers (29% *v* 27%), to have a record of benign breast disease (9% *v* 6%) or other cancers (3.1% *v* 2.6%), or to have a family history of breast cancer (4% *v* 2.5%).

**Table 1:** Characteristics of women with breast cancer and matched controls one year before index date by database (QResearch and CPRD). Values are percentages (numbers) of participants unless stated otherwise

Characteristics	QResearch					CPRD	
	Cases	Controls				Cases	Controls
Total	59 999	269 902				38 612	187 596
Mean (SD) age (years)	63.4 (8.3)	63.6 (8.3)				63.4 (8.3)	63.3 (8.3)
Age group (years):							
50-59	35.7 (21 448)	34.9 (94 327)				36.1 (13 946)	36.6 (68 597)
60-69	37.4 (22 458)	37.6 (101 481)				37.7 (14 557)	37.6 (70 585)
70-79	26.8 (16 093)	27.5 (74 094)				26.2 (10 109)	25.8 (48 414)
Mean (SD) years of records	10.5 (5.5)	10.4 (5.5)				15.4 (5.9)	16.3 (5.6)
Ethnicity:							
Recorded	69.3 (41 584)	70.4 (189 903)				71.3 (27 546)	65.0 (121 959)
White or not recorded	96.1 (57 646)	95.1 (256 792)				97.9 (37 789)	97.7 (183 347)
Bangladeshi	0.1 (73)	0.3 (691)				0.0 (9)	0.0 (85)
Black African	0.3 (191)	0.5 (1450)				0.2 (60)	0.2 (298)
Caribbean	0.7 (439)	0.9 (2299)				0.3 (119)	0.3 (588)
Chinese	0.2 (115)	0.2 (654)				0.1 (48)	0.1 (223)
Indian	1.0 (581)	1.1 (3078)				0.6 (226)	0.6 (1134)
Other	0.7 (424)	0.8 (2216)				0.6 (222)	0.6 (1093)
Other Asian	0.5 (281)	0.5 (1467)				0.2 (79)	0.3 (503)
Pakistani	0.4 (249)	0.5 (1255)				0.2 (60)	0.2 (325)
Townsend fifth*:					*) based on linked cases and controls		
1 (most affluent)	24.7 (14 810)	24.5 (66 100)				27.4 (6201)	27.1 (29 634)
2	23.7 (14 191)	23.2 (62 617)				25.4 (5745)	25.1 (27 488)
3	21.1 (12 630)	21.1 (56 830)				21.0 (4747)	20.9 (22 831)
4	17.5 (10 492)	18.0 (48 495)				17.0 (3847)	17.2 (18 849)
5 (most deprived)	13.1 (7876)	13.3 (35 860)				9.7 (2098)	9.8 (10 702)
Body mass index:							
Recorded	80.3 (48 190)	79.5 (214 580)				90.2 (34 834)	86.5 (162 297)
Mean (SD)	27.5 (5.5)	27.2 (5.5)				27.9 (5.7)	27.6 (5.7)
15-24	30.0 (17 999)	31.6 (85 193)				32.1 (12 405)	32.7 (61 395)
25-29	27.8 (16 696)	27.0 (72 808)				31.3 (12 082)	30.2 (56 570)
≥30	22.5 (13 495)	21.0 (56 579)				26.8 (10 347)	23.6 (44 332)
Smoking status:							
Recorded	87.4 (52 411)	86.7 (233 955)				95.5 (36 890)	91.8 (172 123)
Non-smoker	40.4 (24 241)	41.2 (111 282)				58.5 (22 585)	57.4 (107 617)
Former smoker	32.7 (19 611)	31.6 (85 217)				22.2 (8589)	19.6 (36 761)
Current smoker:							
Light (1-9 cigarettes/day)	7.6 (4549)	7.5 (20 259)				5.9 (2284)	6.1 (11 379)
Moderate (10-19)	4.0 (2419)	4.0 (10 783)				5.5 (2141)	5.3 (9921)
Heavy (≥20)	2.7 (1591)	2.4 (6414)				3.3 (1291)	3.4 (6445)
Alcohol consumption:							
Recorded	79.3 (47 566)	78.3 (211 354)				87.4 (33735)	83.8 (157 295)
None	18.2 (10 949)	19.6 (52 896)				29.5 (11 376)	29.6 (55 519)
Former use	8.7 (5198)	8.8 (23 638)				1.8 (712)	1.6 (2935)
Current user:							
Trivial (<1unit/day)	30.9 (18 547)	30.9 (83 301)				31.9 (12 304)	31.0 (58 125)
Light (1-2 units/day)	11.6 (6949)	10.8 (29 208)				16.7 (6434)	15.0 (28 090)
Moderate (3-6)	9.4 (5610)	7.9 (21 215)				5.9 (2279)	5.4 (10 134)
Heavy (7-9)	0.3 (206)	0.3 (718)				1.1 (416)	0.9 (1714)
Very heavy (≥10)	0.2 (107)	0.1 (378)				0.6 (214)	0.4 (778)
History of other cancers:							
Any cancer	2.8 (1656)	2.4 (6570)				3.6 (1389)	3.0 (5540)
Haematological	0.4 (252)	0.3 (936)				0.5 (180)	0.4 (755)
Cervical	0.2 (114)	0.2 (612)				0.2 (73)	0.2 (440)
Colorectal	0.5 (287)	0.4 (1202)				0.6 (234)	0.5 (976)
Lung	0.1 (77)	0.1 (258)				0.1 (35)	0.1 (256)
Melanoma	0.5 (305)	0.5 (1230)				0.5 (203)	0.4 (660)
Ovarian	0.2 (103)	0.1 (375)				0.3 (110)	0.2 (436)
Uterine	0.2 (101)	0.2 (434)				0.3 (130)	0.3 (481)
Chronic conditions:							
Benign breast disease	8.6 (5174)	5.5 (14 787)				9.3 (3591)	6.0 (11 186)
Diabetes	6.9 (4112)	6.7 (17 972)				7.1 (2757)	6.6 (12 395)
Mental health disorder	0.9 (542)	0.8 (2046)				0.9 (345)	0.8 (1521)
Osteoporosis	3.3 (1985)	3.8 (10171)				4.0 (1558)	4.5 (8469)
Other characteristics:							
Early menopause	13.1 (7831)	14.0 (37 764)				11.0 (4230)	11.9 (22 365)
Late menopause	3.2 (1948)	2.8 (7592)				8.0 (3093)	7.0 (13 225)
Menopausal symptoms	13.2 (7930)	12.9 (34 803)				23.9 (9214)	22.7 (42 579)
Mammography scans*	50.3 (30 170)	49.9 (134 728)				21.3 (8242)	20.1 (37 718)
MRI/CT scans*	6.7 (4004)	6.6 (17 850)				2.6 (1004)	2.6 (4919)
Oophorectomy or hysterectomy	20.7 (12 437)	21.6 (58 412)				21.4 (8264)	21.1 (39 583)
Family history:							
Any cancer	9.6 (5784)	7.9 (21 260)				5.0 (1939)	3.8 (7127)
Breast cancer	4.3 (2598)	2.8 (7656)				3.4 (1329)	2.1 (3901)
Cervical cancer	0.0 (22)	0.0 (112)				0.0 (19)	0.0 (51)
Osteoporosis	0.9 (513)	0.8 (2147)				0.9 (355)	0.9 (1610)
Ovarian cancer	0.2 (93)	0.1 (364)				0.2 (73)	0.2 (286)
Uterine cancer	0.1 (45)	0.1 (172)				0.0 (7)	0.0 (29)
Any use of other drugs before index date:							
Aspirin	15.9 (9533)	16.3 (43 880)				16.3 (6276)	15.9 (29 869)
Non-steroidal anti-inflammatory drugs	62.2 (37 305)	61.5 (166 024)				65.6 (25 329)	64.5 (120 920)
Contraceptive drugs	10.9 (6554)	9.4 (25 494)				17.4 (6735)	17.7 (33 149)
Tamoxifen	0.7 (436)	0.1 (277)				1.0 (389)	0.2 (394)
Raloxifene	0.4 (224)	0.5 (1278)				0.4 (168)	0.5 (858)

MRI=magnetic resonance imaging; CT=computed tomography.

* Based on Hospital Episode Statistics and general practice data for QResearch and on general practice data for Clinical Practice Research Datalink (CPRD).

### Exposure

Across both databases, 33 703 (34%) cases and 142 391 (31%) controls had ever been exposed to HRT. Of those, 8860 (26%) cases and 42 799 (30%) controls had been exposed to oestrogen only therapy and 24 843 (74%) cases and 99 592 (70%) controls had been exposed to oestrogen-progestogen therapy (supplementary eTable 1). Women in the 60-69 age category were relatively more exposed to oestrogen only (47% in cases and controls) and oestrogen-progestogen therapies (48% in cases and controls). A high proportion of women using oestrogen only therapy had undergone oophorectomy or hysterectomy (89% cases and 90% controls), but, overall, users of oestrogen only and oestrogen-progestogen therapies had characteristics broadly similar to those of never users for most confounders. Some women switched between hormones during HRT exposure. About 20% of oestrogen only users had exposure to both oestrogens. About 57% of combined therapy users had only one oestrogen-progestogen combination recorded. About 0.6% of cases and controls started HRT in the year before the index date, but these are considered as never users in the analyses.

### Overall exposure

Overall (or ever) exposure to HRT was associated with an increased risk of breast cancer (adjusted odds ratio 1.21, 95% confidence interval 1.19 to 1.23). The increased risk was mostly attributable to oestrogen-progestogen therapy (1.26, 1.24 to 1.29), with oestrogen only therapy showing a small increased risk (1.06, 1.03 to 1.10), both compared with never users (supplementary eTable 2). No increased risk was associated with oestrogen cream or vaginal preparations (supplementary eTable 3). The risks associated with HRT increased with duration of use, but the associations were less strong for oestrogen only therapy and for tibolone than for oestrogen-progestogen therapy, apart from estradiol-dydrogesterone preparations. Norethisterone, levonorgestrel, and medroxyprogesterone were associated with similar risks, increasing across all duration categories longer than one year. For all exposure durations, the combined treatment with the lowest associated risk increase was estradiol-dydrogesterone. No differences were found between low and high doses of oestrogens or between different application methods for estradiol, norethisterone, or levonorgestrel (supplementary eTable 4).

Associations between use of HRT and risk of breast cancer rapidly decreased with increasing years of discontinuation (supplementary eFigure 1 and eTable 5). For oestrogen only, estradiol combined with norethisterone and dydrogesterone, and tibolone, no significantly increased risk was found from two years after discontinuation. For medroxyprogesterone, the risk was reduced after two years but remained raised until after five years; for levonorgestrel until after 10 years.

### Duration of recent and past exposures as categorical variables

**Fig 2:**
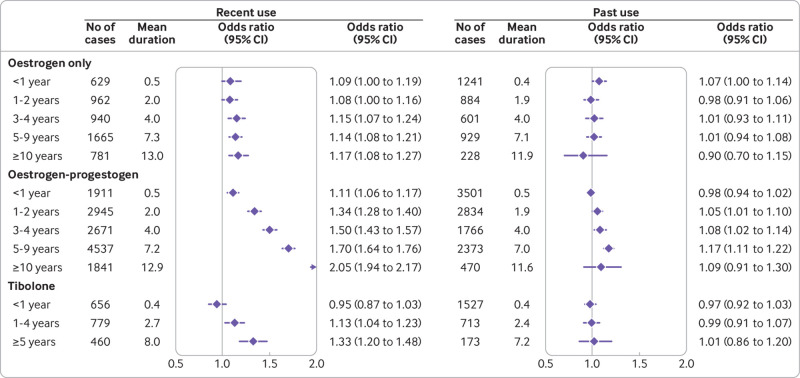
Recent and past use of oestrogen, oestrogen-progestogen, and tibolone in association with breast cancer risk. Odds ratios are with reference to never users and adjusted for smoking status, alcohol consumption, Townsend fifth (QResearch only), body mass index, ethnicity, history of other cancers, oophorectomy or hysterectomy, records of early and late menopause, menopausal symptoms, mammography or scans, family history, comorbidities, other drugs, and years of data. Cases are matched to controls by age, general practice, and index date

**Fig 3:**
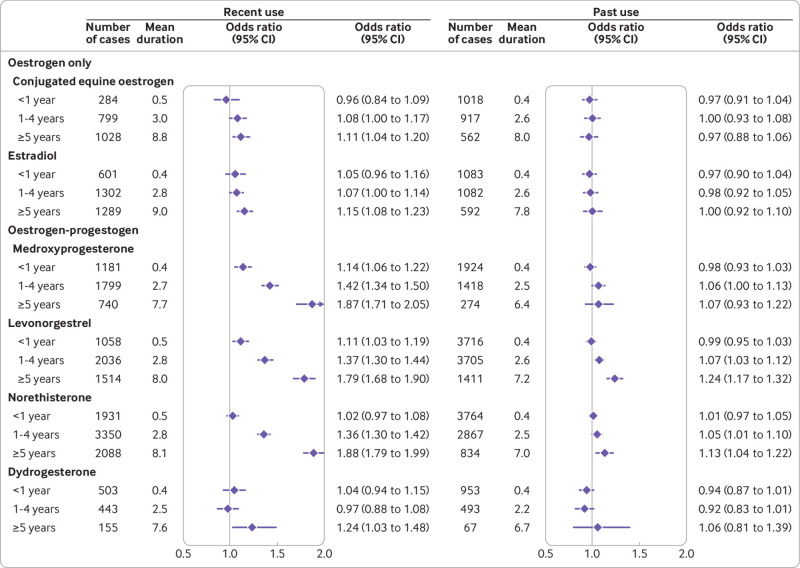
Recent and past use of different hormones in association with breast cancer risk. Odds ratios are with reference to never users and adjusted for smoking status, alcohol consumption, Townsend fifth (QResearch only), body mass index, ethnicity, history of other cancers, oophorectomy or hysterectomy, records of early and late menopause, menopausal symptoms, mammography or scans, family history, comorbidities, other drugs, and years of data. Cases are matched to controls by age, general practice, and index date

Recent users of HRT (ie, those with prescriptions more than one year and less than five years before the index date) comprised 56% (18 879) of cases and 50% (70 931) of controls ever exposed to HRT. Figure 2 and figure 3 (supplementary eTable 6) show the associations between categorised durations of HRT and risks of breast cancer in women with recent and past exposures. The patterns of risks for recently exposed women were similar to those for overall exposures, but the risks were consistently higher and more pronounced, particularly for oestrogen-progestogen therapy. For women with past exposures, risks associated with longer durations of use of oestrogen-progestogen, particularly longer use of levonorgestrel (>3 years) and norethisterone (>5 years), remained high, but for other hormones the risks were not statistically significant. Findings for recent exposures to different doses and applications also had similar patterns to the overall exposure analysis, but with higher odds ratios (supplementary eTable 7).

A further restriction to recency (defined now as one prescription or more in the period 1-2 years before the index date) resulted in fewer women in each category of exposure, but the increased risks associated with longer exposures were even more pronounced (supplementary eFigure 2). In HRT users, 40% (13 463) of cases and 32% (44 972) of controls ever exposed to HRT had one or more prescriptions in the period 1-2 years before the index date. The patterns of risks for these recently exposed women were similar to those of overall exposures, but the risks were consistently higher and more pronounced for progestogens. For women with a last exposure more than two years before the index date, risks associated with long exposures to levonorgestrel (>3 years) remained high, but for other hormones the risks were not statistically significant (supplementary eFigure 3 and eTable 8).

### Duration of recent and past exposures as continuous variables

**Fig 4:**
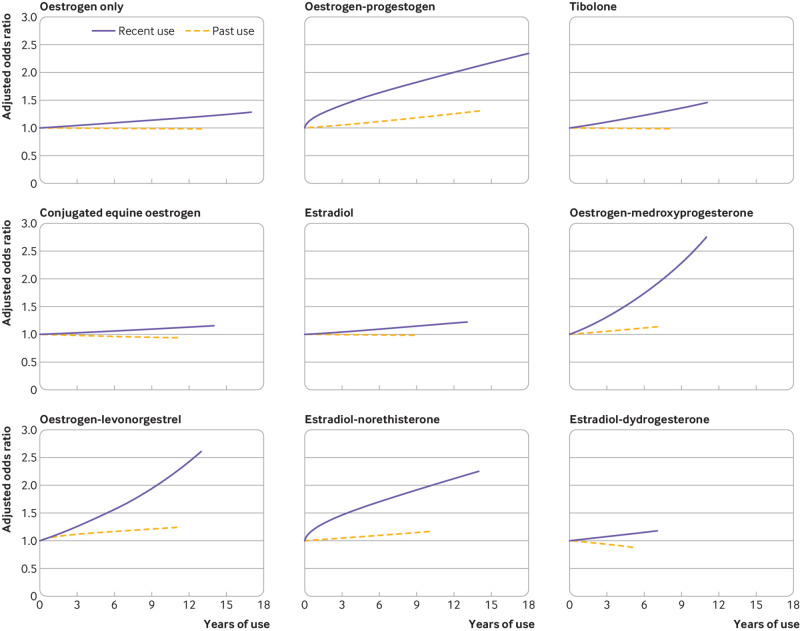
Adjusted odds ratios for different durations of recent and past exposures to hormone replacement therapies in association with breast cancer risk. Odds ratios are with reference to never users and adjusted for smoking status, alcohol consumption, Townsend fifth (QResearch only), body mass index, ethnicity, history of other cancers, oophorectomy or hysterectomy, records of early and late menopause, menopausal symptoms, mammography or scans, family history, comorbidities, other drugs, and years of data. Cases are matched to controls by age, general practice, and index date. Model includes fractional polynomial terms for recent use of oestrogen-progestogen (power 0.5), estradiol-norethisterone (power 0.5), past use of oestrogen-levonorgestrel (power 0.5), and linear terms (1) for all other exposures

Figure 4 (supplementary eTable 6) shows the associations between duration of different types of HRT and risks of breast cancer for recent (1-5 years before index date) and past users (prescriptions ≥5 years previously). A linear relation was found between duration of exposure as a continuous variable for most types of HRT, with risk increasing uniformly over time. However, for recent exposure to oestrogen-progestogen or to estradiol-norethisterone, and for past exposure to oestrogen-medroxyprogesterone, square root transformations gave the best fit for an association with breast cancer risk, showing that risk for these treatments increased faster earlier in the exposure. Additions of further fractional polynomial terms were not statistically significant.

Risk increases for recent users were more pronounced than for past users, and different types of HRT showed different patterns of increase as the durations of exposure increased. Oestrogen-medroxyprogesterone and oestrogen-levonorgestrel formulations showed the greatest increases with duration. Oestrogen only (including separately conjugated equine oestrogen and estradiol), tibolone, and estradiol-dydrogesterone formulations showed the smallest increases with duration.

### Subgroup analyses

**Fig 5:**
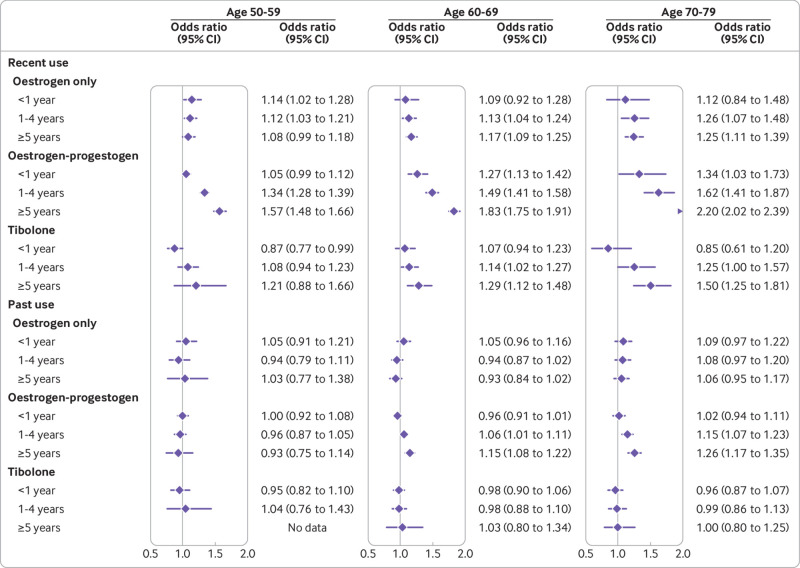
Use of oestrogen only, oestrogen-progestogen, and tibolone in women of different ages in association with breast cancer risk. Odds ratios are with reference to never users and adjusted for smoking status, alcohol consumption, Townsend fifth (QResearch only), body mass index, ethnicity, history of other cancers, oophorectomy or hysterectomy, records of early and late menopause, menopausal symptoms, mammography or scans, family history, comorbidities, other drugs, and years of data. Cases are matched to controls by age, general practice, and index date

The subgroup analyses for different age categories showed similar patterns in magnitudes of risk for recent and past exposures (fig 5, supplementary eFigure 4 and eTables 9 and 10). The oldest age group (70-79) had a smaller number of recent (1-5 years before the index date) users and, although odds ratios appeared to be higher than for the younger age groups, the confidence intervals were too wide to reach statistical significance for oestrogen only users. The younger age group (50-59) had the lowest odds ratios, which could reflect shorter durations of exposure, particularly in the category of five years or more (supplementary eTables 9 and 10). The mean duration for the category of 1-4 years, however, was only slightly lower for the younger group but similar between the older groups.

**Fig 6:**
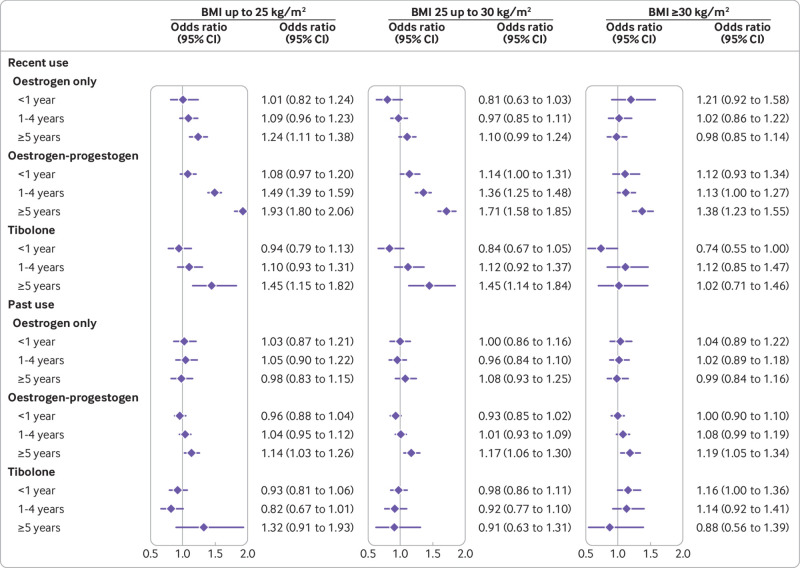
Use of oestrogen-only, oestrogen-progestogen, and tibolone in women of different body mass index in association with breast cancer risk. Odds ratios are with reference to never users and adjusted for smoking status, alcohol consumption, Townsend fifth (QResearch only), body mass index, ethnicity, history of other cancers, oophorectomy or hysterectomy, records of early and late menopause, menopausal symptoms, mammography or scans, family history, comorbidities, other drugs, and years of data. Cases are matched to controls by age, general practice, and index date

Figure 6 presents the associations with breast cancer risk for recent and past exposures in different BMI categories (supplementary eFigure5 and eTables 11 and 12). Overall, the pattern of risks in the subgroups were similar to those of the main analyses. For women with a higher BMI (>30), however, the risks associated with HRT for recent users appeared slightly lower than in women with a lower BMI, both for oestrogen only and for oestrogen-progestogen therapies. For oestrogen only therapy and more than five years of use, the association with risk of breast cancer was statistically significant only in the lowest BMI group (1.24, 1.11 to 1.35) compared with never use. For oestrogen-progestogen, more than five years of use was associated with the highest adjusted odds ratio in the lowest BMI group and the lowest adjusted odds ratio in the highest BMI group (1.93, 1.80 to 2.05 for BMI <25; 1.71, 1.58 to 1.85 for BMI 25-30; and 1.38, 1.23 to 1.55 for BMI >30). For past use, no difference between BMI groups was observed.

### Excess numbers in HRT users

The crude incidence rate of breast cancer in the underlying CPRD cohort was 33.0 (95% confidence interval 32.7 to 33.3) per 10 000 women years, whereas the crude incidence rate in women not exposed to HRT was 31.5 (31.1 to 31.7) per 10 000 women years. The rate for unexposed women varied with age, with the lowest rate in younger women (28.2, 27.6 to 28.7 in women aged 50-59; 34.1, 33.4 to 34.8 in women aged 60-69; and 33.3, 32.6 to 34.0 in women aged 70-79). The highest rate in the 60-69 years group was consistent with national data from cancer registration statistics in England.^22^

**Fig 7:**
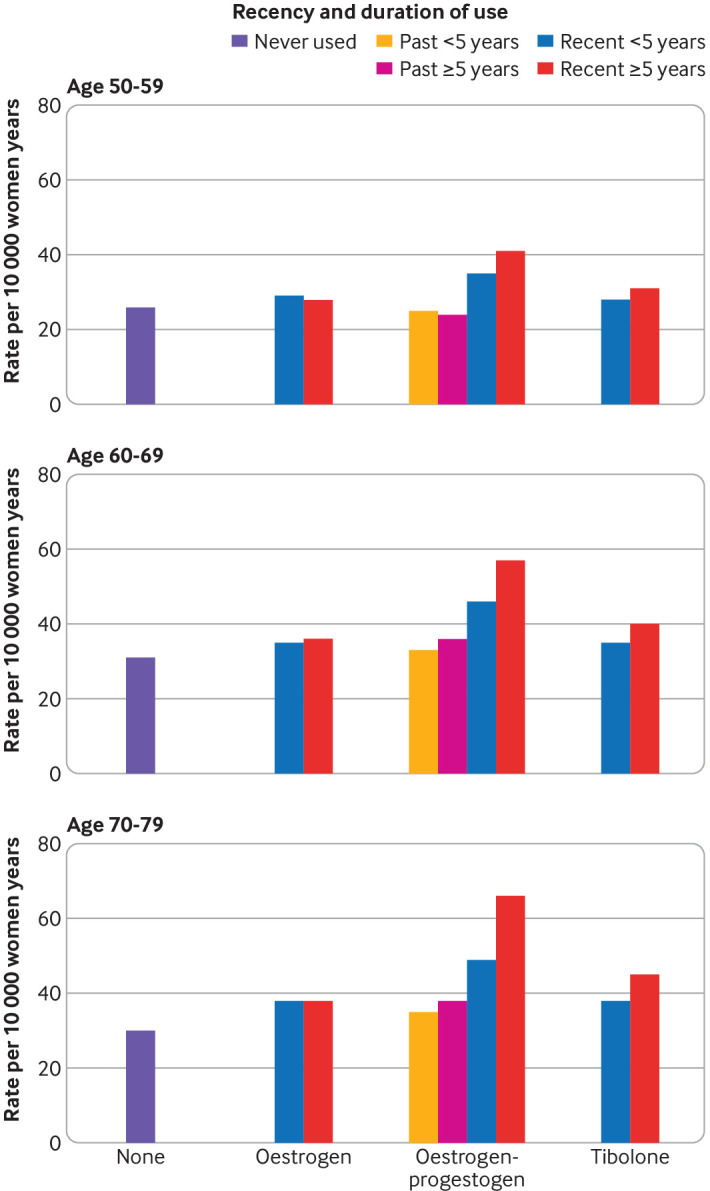
Incident breast cancer rate per 10 000 women years for women unexposed and exposed for different durations to different hormone replacement therapies by age range. Rates were estimated using rates in unexposed populations multiplied by adjusted odds ratios derived from subgroup analyses for different age categories (see fig 5)

Table 2 and fig 7 contain incidence rates and excess rates of breast cancer in users of HRT at different ages and for different durations. The number of extra cases is consistently larger for older women for all exposures. Compared with never users, the estimated number of excess cases per 10 000 women years in recent long term (≥5 years) users of oestrogen only treatment was three in women aged 50-59, four in women aged 60 to 69, and eight in women aged 70-79. Compared with never users, the number of excess cases per 10 000 women years in recent long term users of oestrogen-progestogen treatment was 15 in women aged 50-59, 26 in women aged 60-69, and 36 in women aged 70-79. For tibolone in recent long term users, the numbers exposed in younger women were too small to provide sufficient data, but within the older groups there are an estimated nine extra cases per 10 000 women years in women aged 60 to 69 and 15 extra cases per 10 000 women years in women aged 70 to 79.

**Table 2:** Incidence rates and excess of cases of breast cancer compared with never use per 10 000 women years by different age categories and different durations and recency of hormone replacement therapy (HRT) use

HRT and duration of use by recency	Age group (years)					
	50-59		60-69		70-79	
	Rate per 10 000	Extra cases	Rate per 10 000	Extra cases	Rate per 10 000	Extra cases
Never use	26	–	31	–	30	–
Oestrogen only:						
Recent use 1-5 years	29	3	35	4	38	8
Recent use ≥5 years	28	–	36	5	38	8
Oestrogen-progestogen:						
Past use 1-5 years	25	–	33	2	35	5
Past use ≥5 years	24	–	36	5	38	8
Recent use 1-<5 years	35	9	46	15	49	19
Recent use ≥5 years	41	15	57	26	66	36
Tibolone:						
Recent use 1-<5 years	28	–	35	4	38	8
Recent use ≥5 years	31	–	40	9	45	15

Rates were estimated using rates in unexposed populations from Clinical Practice Research Datalink multiplied by adjusted odds ratios derived from subgroup analyses for different age categories (see fig 5). Extra cases were reported only for statistically significant findings. Recent use is within one and five years; past use is five years before the index date.

### Sensitivity analyses

The sensitivity analysis run on women with at least 10 years of recorded data showed similar patterns of risks associated with different durations of HRT use, but the risks appeared slightly higher, particularly for exposure to oestrogen-progestogen combinations of between five and 10 years (supplementary eFigure 6 and eTable 13). The sensitivity analysis on the subgroup of women aged 55 to 79 showed similar patterns of risks, with all values consistent with the subgroup analyses for different age groups (supplementary eFigure 6 and eTable 14). The sensitivity analysis with duration of exposure defined as from the first prescription of HRT to the end of the last prescription showed results similar to those of the main analysis (supplementary eTable 15). The results of analyses run on cases and controls without missing data for smoking status, alcohol consumption, and BMI were similar to those of the main analyses—as were the analysis restricted to CPRD cases and controls with linked data.

## Discussion

This large observational study found that exposure to most HRT drugs is associated with an increased risk of breast cancer. In comparison with a recent meta-analysis, however, our findings generally suggest lower increased risk associations between longer term HRT use and breast cancer, and we report a more noticeable decline in risks once HRT has stopped. Risk increases were mostly associated with oestrogen-progestogen treatments, but small increases were also associated with oestrogen only treatments. For all exposure durations, the combined treatment with the lowest associated risk increase was estradiol-dydrogesterone. Associations for all treatments depended on duration, with no increased risks for less than one year of treatment but increasing risks for longer exposures to medroxyprogesterone, norethisterone, and levonorgestrel. Associations were more pronounced for older women and less noticeable for obese women.

### Strengths and weaknesses of this study

The main strengths of this original study are its size, consistent sources of primary care data, almost complete follow-up of diagnoses using linked data, consistent design, and resulting generalisability of the findings. Combining results from the two largest UK primary care research databases with national coverage has provided increased power, representiveness, and wide geographical coverage of included practices. The study relates to an important health problem and used only objective information on prescriptions for HRT in the UK, facilitating inclusion of the full range of preparations available within this national setting and presenting in detail the increased risk of breast cancer associated with usage patterns. The follow-up and validation of breast cancer diagnoses through linkages to hospital, mortality, and cancer registry (QResearch only) data reduced both ascertainment and recording bias. As our study was based on routinely collected data, it was also not susceptible to recall bias. Matching by general practice enabled consideration of possible differences in prescribing and recording patterns across practices. Differences in menopause onset and age at start of HRT were partially dealt with through matching by age. The results were also adjusted for information on lifestyle, comorbidities, and use of other drugs, and the study presents subgroup analyses for women in different age groups and in different BMI categories. Protopathic bias, from diagnostic and prescribing problems created by symptoms common to early breast cancer and onset of menopause, was minimised by excluding prescriptions issued in the year before the index date.

Some limitations of this study arise from inevitable shortfalls in completeness and accuracy within any routinely collected dataset. A small proportion of women had missing information on smoking status, alcohol consumption, and BMI, but these were dealt with by multiple imputation. As we did not have reliable data for age at onset of menopause for all women, we estimated onset from the first menopause specific record before the earliest HRT prescription. For women with no such record we assumed onset within the most common age range of 50 to 54 years. We did not investigate the differences between continuous and sequential HRT because these regimens are prescribed at different times after menopause. As our cases and controls were matched by age, they would likely have been prescribed similar regimens, making a comparison infeasible. Our primary focus, anyway, was recent long term exposure.

No reliable data were available for established risk factors for breast cancer, such as parity or time of the first pregnancy, but there is no evidence to show that these are related to HRT use. No data for physical activity were available, but a possible risk reduction for active women has been shown not to be influenced by menopausal status.^23^ Some women might have joined their current practice after the onset of menopause, so records of past treatments might not have been available. The results from the subgroup restricted to women with at least 10 years of data, however, showed a similar pattern of risk associations to that of the main analysis. Use of HRT might have the side effect of increased breast density, possibly masking cancers and leading to diagnostic delays,^24^ which could shift odds ratios for short duration towards unity. Also, although there was no information about adherence to HRT, any systematic differences between cases and controls seems unlikely because information was recorded prospectively before diagnosis. Conversations with lay women involved in this research also revealed a high adherence to HRT.

### Strengths and weaknesses in relation to other studies

Our study used a nested case-control design, so it did not follow women prospectively from the start of HRT or assess average lifetime risks. Rather, it looked back at already recorded exposures to HRT for women with a diagnosis of breast cancer and matched controls in the age range 50 to 79 and produced comparisons of risks averaged across all time points at which diagnoses in the datasets occurred. The study is based on data derived from real world treatment settings, when women might not have had a constant supply of a preparation and might have needed to switch drugs during the study period. Including all exposures prescribed over time allowed us to present information for a wide range of common types of HRT.

Most trials produced results for a more restricted number of treatments. A meta-analysis of existing trials,^7^ taken largely from the Women’s Health Initiative study, provided estimates only for the specific treatments of conjugated equine oestrogen with and without medroxyprogesterone.^6^ In contrast to our estimates of a slightly increased risk for long term users of conjugated equine oestrogen (average duration for recent exposure of 5.6 years, odds ratio 1.07, 95% confidence interval 1.01 to 1.12), the meta-analysis found no difference in risk of breast cancer (relative risk 0.79, 95% confidence interval 0.61 to 1.02) after a mean duration of 7.2 years. The observed relative risk for the combined conjugated equine oestrogen with medroxyprogesterone therapy after a mean duration of 5.6 years (1.27, 1.03 to 1.56) was similar to our findings for recent exposure, with an average duration of 3.7 years (odds ratio 1.35, 1.30 to 1.41).

Our estimates were consistent with previous observational studies.^9^ ^25^ ^26^ ^27^ The Million Women Study^28^ ^29^ showed slightly higher risks than our study: for recent oestrogen only users a relative risk of 1.30 (95% confidence interval 1.21 to 1.40) compared with our odds ratio of 1.12 (1.08 to 1.16), and for recent oestrogen-progestogen users a relative risk of 2.00 (1.88 to 2.12) compared with our odds ratio of 1.51 (1.47 to 1.54). However, the Million Women Study only covered a selected population of women who had undergone mammography, and the initial study used just a single baseline questionnaire to collect information.^28^ Taken together, the relatively high proportion of HRT users in the initial study (55% were ever users and 35% were current users) and the less than 65% response rate at three years of follow-up, which would be expected also to be skewed towards HRT users, would suggest that women who used HRT were more likely to have participated.^29^

In general, some inconsistency was found between the proportions of women exposed to HRT in data used for our study and those used in the 2019 meta-analysis.^11^ The predominant (40%) data source for the meta-analysis was from the Million Women Study, where, 51% of cases had ever been exposed to HRT and 18% of cases were current users (<5 years). The second largest data source, comprising 28% of the data used in the meta-analysis, was routinely collected CPRD data (one of the two data sources in our study), and here 40% of women with breast cancer had been exposed to HRT and 12% were current users. Both these exposure rates contrast with those in our study, which overall had 34% of cases ever exposed and 19% of cases with prescriptions within 1-5 years before diagnosis.

We cannot speculate on reasons for these differences in the CPRD data used because we do not have access to relevant information for the 2019 meta-analysis. That sample contained slightly older cases (mean age at diagnosis 66 *v* 63 in our sample) but no age range was reported. The estimations of risk for overall use of HRT in our CPRD analysis (odds ratio 1.21, 95% confidence interval 1.18, 1.25) were, however, similar to the CPRD specific estimations reported in the meta-analysis (relative risk 1.25, 95% confidence interval 1.20, 1.30). For recent use (prescriptions 1-2 years before the index date), when the proportions of HRT users in CPRD data used in our study and in the meta-analysis were closest, the estimates of risk were also similar. In our analysis of CPRD data on recent use, the odds ratios were 1.25 (1.17 to 1.34) for oestrogen only and 1.91 (1.83 to 1.99) for oestrogen-progestogen, whereas for the CPRD data used in the meta-analysis the corresponding findings were 1.36 (1.25 to 1.4) and 2.16 (2.02 to 2.31).

Comparative assessment of the findings from the 2019 meta-analysis is in general complicated by the heterogeneity of included studies and data sources. The meta-analysis included data from 24 differently designed prospective studies from around the world. Differences between findings from our large, consistently designed study and those from the meta-analysis might be related to the different periods covered by included studies or several problems relating to the different data sources. Some studies used routinely collected data with different definitions of exposure,^9^ ^30^ some used questionnaires with a single baseline assessment of exposure,^27^ ^28^ ^31^ and others used repeated biennial questionnaires.^25^ ^32^ Some participants were recruited from different countries with ever exposure levels varying from 19% to 69%,^8^ and some studies were from different profession related populations.^8^ ^25^

Overall, our results were broadly in line with those of the meta-analysis^11^ but with slightly lower risks for long term exposures. This might partly be explained by almost half of the cases in the meta-analysis coming from the Million Women Study. For current use, however, the meta-analysis reported similar associations with risk of breast cancer, regardless of whether such use was restricted to HRT exposure within the past five years or within the past two years. By contrast, we found associations to be more pronounced for users with a prescription recency of 1-2 years before the index date, with higher odds ratios than for an exposure recency of 1-5 years. Our results with a recency definition of 1-2 years were broadly similar to those of the meta-analysis for either of their definitions of current use, whereas our findings for 1-5 years recency were lower. The difference in risk found by us seems to be more in line with previous expectations of declines in risk after cessation of HRT.^7^

Our findings for oestrogen only users with recent (1-5 years) use of more than five years (odds ratio 1.15, 95% confidence interval 1.09 to 1.21) were lower than those from the meta-analysis (relative risk 1.33, 95% confidence interval 1.28 to 1.38).^11^ Our study also found a marginally higher risk associated with estradiol than with conjugated equine oestrogen. For oestrogen-progestogen therapy, our finding for recent use of more than five years duration was also lower (1.79, 1.73 to 1.85) than the meta-analysis estimate (2.08, 2.02 to 2.15). Despite the similar average duration of exposures between our study and those in the meta-analysis, our findings for the different types of the most common progestogens and tibolone were consistently lower than those of the meta-analysis (supplementary eTable 16).

For dydrogesterone, our study found lower risks associated with more than five years of exposure than in the meta-analysis (1.24, 1.03 to 1.48 *v* 1.41, 1.17 to 1.71).^11^ The risk from dydrogesterone was much lower than for any other progestogen, but one of our sensitivity analyses did show a statistically significant increased risk for a small subgroup of dydrogesterone users—those with a prescription 1-2 years before the index date and more than five years of use (112 cases, odds ratio 1.47, 1.19 to 1.83).

Our study showed differential risks associated with HRT use by age category. For recent exposures of more than five years’ duration, associated risks of breast cancer rose with increasing age category. This might partly be explained by generally longer usages in older age categories, although exposure of 1-4 years was similarly associated with increasing risk from younger to older age groups of women. Our findings for the 70-79 age group for oestrogen only use (1.25, 1.11 to 1.39) and for oestrogen-progestogen use (2.20, 2.02 to 2.39) are in line with findings from the meta-analysis^11^ for women who started HRT at the age of 55-59 and continued treatment for 5-14 years : oestrogen only use of 1.26 (1.12 to 1.41) and oestrogen-progestogen use of 1.97 (1.81 to 2.15).

The adiposity of included women differed between our study and previous studies. Mean BMI in our study (27.7 in cases) was higher than in other observational studies (average 25)^33^ but slightly lower than in the Women’s Health Initiative trial (28.5).^34^ This could help to explain overall differences in associations between our findings and those of other studies, although the mean BMI in our study reflects the distribution within women with breast cancer diagnosed in the general UK population over the study period. Our findings for women matched by age and category of BMI are detailed and comprehensive estimations of duration dependent associations for HRT exposure and breast cancer risk. They are broadly similar to those from previous studies and the 2019 meta-analysis,^11^ ^33^ with the lowest associations between HRT use and risk of breast cancer in women in the highest BMI category. These concur with findings from the Million Women Study (which had relatively small numbers) and an earlier meta-analysis.^29^ ^35^ Some complex biological relation might exist between fat tissue and HRT,^36^ although it might also be related to differences in timeliness of diagnoses between women with different body weights.

### Implications for clinicians and policymakers

This study delivers more generalisable estimates of the different risks of breast cancer associated with specific progestogen components of HRT, while confirming no increased risks from short term use of oestrogen only, estradiol-dydrogesterone, and tibolone. Increasing duration of use was generally associated with increased risk, with tibolone and estradiol-dydrogesterone showing the smallest risks. The frequency of prescribing for treatments including dydrogesterone was, however, much lower than for those including norethisterone, medroxyprogesterone, or levonorgestrel.

### Unanswered questions and future research

In our study protocol we did not prespecify analyses relating to cancer stage or tumour type because these lay outside the main question of interest. Although information on risk related to individual progestogens could be improved, previous studies have shown that the associated risks between HRT and tumour types might differ, with higher risks of developing oestrogen receptor positive tumours and lobular tumours.^11^ Knowing the cancer stage could also address the question of risk differences between women of various body weights, to clarify whether systematic differences might exist in diagnostic delay. Other unknowns include questions about breast cancer survival rates and all cause mortality in women using HRT.^13^

### Conclusion

This large observational study of HRT and breast cancer risk based on two large primary care databases analysed in an identical manner has confirmed the excess risk to be attributable mostly to combined treatments, with the lowest risks associated with use of the least commonly prescribed dydrogesterone. Rarely prescribed tibolone also showed low increased risks.

Our findings of generally lower increased risks for combined HRT treatments and of more pronounced declines in risk once HRT has stopped, provide some counterbalance to the higher than expected risks reported in a recently published meta-analysis.^11^ Our results add more evidence to the existing knowledge base and should help doctors and women to identify the most appropriate HRT formulation and treatment regimen, and provide more consistently derived information for women’s health experts, healthcare researchers, and treatment policy professionals.

**What is already known on this topic**

Long term systemic use of hormone replacement therapy (HRT) is associated with increased risks of breast cancer, mostly attributable to the progestogens medroxyprogesterone, norethisterone, and levonorgestrel
After discontinuation of treatment, the increased risks decline, but remain raised for some years
A recent large meta-analysis has reported higher than expected breast cancer risks associated with HRT

**What this study adds**

The study confirmed increased risks of breast cancer associated with long term use of oestrogen only therapy and combined oestrogen and progestogen therapy
The combined treatment associated with the lowest risk increase was estradiol-dydrogesterone
The findings suggest lower increased risks of breast cancer associated with longer term HRT use, and a more noticeable decline in risks once treatment is stopped compared with the meta-analysis

## Web extra

Extra material supplied by authors

Supplementary information: Tables e1-e16 and figures e1-e7

## Notes

Contributors: YV contributed to the study protocol, reviewed the literature, designed the study, organised the extraction of CPRD data, did the analysis on both datasets and wrote the draft of the manuscript. JHC initiated the study, undertook the original literature review, drafted the study protocol, organised the extraction of the QResearch data, advised on the design and clinical aspects of the study and interpretation of the results and drafting of the paper. CC contributed to the development of the idea and the study design and advised on the analysis and interpretation of the results. JHC and CC critically reviewed the paper. YV is the guarantor of the study. All authors have approved the submitted version. The corresponding author attests that all listed authors meet authorship criteria and that no others meeting the criteria have been omitted.

Ethical approval: The protocol for QResearch has been published in ePrints and was reviewed in accordance with the requirements for the East Midlands Derby Research Ethic Committee (ref 03/4/021). The protocol for CPRD has been approved by the Independent Scientific Advisory Committee for MHRA Database Research (N 16_282).

Data sharing: To guarantee the confidentiality of personal and health information only the authors have had access to the data during the study in accordance with the relevant licence agreements. Access to the QResearch data are according to the information on the QResearch website (www.qresearch.org). CPRD linked data were provided under a licence that does not permit sharing.

The lead author affirms that the manuscript is an honest, accurate, and transparent account of the study being reported; that no important aspects of the study have been omitted and that any discrepancies from the study as planned have been explained.

Dissemination to participants and related patient and public communities: The results of the study will be sent to University of Nottingham press office, to related patients, and to the funders. A lay summary will be created and published at SPCR NIHR and QResearch website.

Provenance and peer review: Not commissioned; externally peer reviewed.

## References

[1] Marjoribanks J Farquhar C Roberts H Lethaby A Lee J Long-term hormone therapy for perimenopausal and postmenopausal women. Cochrane Database Syst Rev 2017;1:CD004143. 10.1002/14651858.CD004143.pub5 28093732 PMC6465148

[2] Parkin DM 10. Cancers attributable to exposure to hormones in the UK in 2010. Br J Cancer 2011;105(Suppl 2):S42-8. 10.1038/bjc.2011.483 22158320 PMC3252057

[3] Cancer Research UK. Risk factors for Breast Cancer. 2015. https://www.cancerresearchuk.org/health-professional/cancer-statistics/statistics-by-cancer-type/breast-cancer/risk-factors.

[4] National Collaborating Centre for Women’s and Children’s Health. Menopause. Full guideline. National Institute for Health and Care Excellence. 2015 (Version 1.5) 26598775

[5] National Institute for Health and Care Excellence. Menopause: diagnosis and management. NICE guideline NG23. 2015. 33141539

[6] Gartlehner G Patel SV Feltner C Hormone therapy for the primary prevention of chronic conditions in postmenopausal women: Evidence report and systematic review for the US preventive services task force. JAMA 2017;318:2234-49. 10.1001/jama.2017.16952 29234813

[7] Chlebowski RT Rohan TE Manson JE Breast Cancer After Use of Estrogen Plus Progestin and Estrogen Alone: Analyses of Data From 2 Women’s Health Initiative Randomized Clinical Trials. JAMA Oncol 2015;1:296-305. 10.1001/jamaoncol.2015.0494 26181174 PMC6871651

[8] Bakken K Fournier A Lund E Menopausal hormone therapy and breast cancer risk: impact of different treatments. The European Prospective Investigation into Cancer and Nutrition. Int J Cancer 2011;128:144-56. 10.1002/ijc.25314 20232395

[9] Román M Graff-Iversen S Weiderpass E Postmenopausal Hormone Therapy and Breast Cancer Prognostic Characteristics: A Linkage between Nationwide Registries. Cancer Epidemiol Biomarkers Prev 2016;25:1464-73. 10.1158/1055-9965.EPI-16-0240 27461048

[10] Lee S Kolonel L Wilkens L Wan P Henderson B Pike M Postmenopausal hormone therapy and breast cancer risk: the Multiethnic Cohort. Int J Cancer 2006;118:1285-91. 10.1002/ijc.21481 16170777

[11] Collaborative Group on Hormonal Factors in Breast Cancer Type and timing of menopausal hormone therapy and breast cancer risk: individual participant meta-analysis of the worldwide epidemiological evidence. Lancet 2019;394:1159-68. 10.1016/S0140-6736(19)31709-X 31474332 PMC6891893

[12] Fournier A Berrino F Clavel-Chapelon F Unequal risks for breast cancer associated with different hormone replacement therapies: results from the E3N cohort study. Breast Cancer Res Treat 2008;107:103-11. 10.1007/s10549-007-9523-x 17333341 PMC2211383

[13] Rymer J Brian K Regan L HRT and breast cancer risk. BMJ 2019;367:l5928. 10.1136/bmj.l5928 31604711

[14] Vinogradova Y, Coupland C, Hippisley-Cox J. Protocol to assess risk of breast cancer associated with use of hormone replacement therapy in real world settings: two nested case-control studies in primary care. 2019. https://nottingham-repository.worktribe.com/output/2309731.

[15] Etminan M Pharmacoepidemiology II: the nested case-control study—a novel approach in pharmacoepidemiologic research. Pharmacotherapy 2004;24:1105-9. 10.1592/phco.24.13.1105.38083 15460170

[16] Delgado-Rodríguez M Llorca J Bias. J Epidemiol Community Health 2004;58:635-41. 10.1136/jech.2003.008466 15252064 PMC1732856

[17] Royston P Multiple imputation of missing values. Stata J 2004;4:227-41 10.1177/1536867X0400400301.

[18] Borenstein M Hedges LV Higgins JP Rothstein HR A basic introduction to fixed-effect and random-effects models for meta-analysis. Res Synth Methods 2010;1:97-111. 10.1002/jrsm.12 26061376

[19] Royston P Ambler G Sauerbrei W The use of fractional polynomials to model continuous risk variables in epidemiology. Int J Epidemiol 1999;28:964-74. 10.1093/ije/28.5.964 10597998

[20] Manson JE Chlebowski RT Stefanick ML Menopausal hormone therapy and health outcomes during the intervention and extended poststopping phases of the Women’s Health Initiative randomized trials. JAMA 2013;310:1353-68. 10.1001/jama.2013.278040 24084921 PMC3963523

[21] Collaborative Group on Hormonal Factors in Breast Cancer Menarche, menopause, and breast cancer risk: individual participant meta-analysis, including 118 964 women with breast cancer from 117 epidemiological studies. Lancet Oncol 2012;13:1141-51. 10.1016/S1470-2045(12)70425-4 23084519 PMC3488186

[22] Office for National Statistics. Cancer registration statistics, England: 2017. 2019 www.ons.gov.uk/peoplepopulationandcommunity/healthandsocialcare/conditionsanddiseases/bulletins/cancerregistrationstatisticsengland/2017.

[23] Pizot C Boniol M Mullie P Physical activity, hormone replacement therapy and breast cancer risk: A meta-analysis of prospective studies. Eur J Cancer 2016;52:138-54. 10.1016/j.ejca.2015.10.063 26687833

[24] Zahl P-H Mæhlen J Bias in Observational Studies of the Association between Menopausal Hormone Therapy and Breast Cancer. PLoS One 2015;10:e0124076. 10.1371/journal.pone.0124076 25938446 PMC4418576

[25] Colditz GA Hankinson SE Hunter DJ The use of estrogens and progestins and the risk of breast cancer in postmenopausal women. N Engl J Med 1995;332:1589-93. 10.1056/NEJM199506153322401 7753136

[26] Prentice RL Chlebowski RT Stefanick ML Estrogen plus progestin therapy and breast cancer in recently postmenopausal women. Am J Epidemiol 2008;167:1207-16. 10.1093/aje/kwn044 18372396 PMC2670848

[27] Saxena T Lee E Henderson KD Menopausal hormone therapy and subsequent risk of specific invasive breast cancer subtypes in the California Teachers Study. Cancer Epidemiol Biomarkers Prev 2010;19:2366-78. 10.1158/1055-9965.EPI-10-0162 20699377 PMC2936672

[28] Million Women Study Collaborators Breast cancer and hormone-replacement therapy in the Million Women Study. Lancet 2003;362:419-27. 12927427 10.1016/s0140-6736(03)14065-2

[29] Beral V Reeves G Bull D Green J Million Women Study Collaborators Breast cancer risk in relation to the interval between menopause and starting hormone therapy. J Natl Cancer Inst 2011;103:296-305. 10.1093/jnci/djq527 21278356 PMC3039726

[30] Opatrny L Dell’Aniello S Assouline S Suissa S Hormone replacement therapy use and variations in the risk of breast cancer. BJOG 2008;115:169-75, discussion 175. 10.1111/j.1471-0528.2007.01520.x 18081598

[31] Bakken K Alsaker E Eggen AE Lund E Hormone replacement therapy and incidence of hormone-dependent cancers in the Norwegian Women and Cancer study. Int J Cancer 2004;112:130-4. 10.1002/ijc.20389 15305384

[32] Fournier A Berrino F Riboli E Avenel V Clavel-Chapelon F Breast cancer risk in relation to different types of hormone replacement therapy in the E3N-EPIC cohort. Int J Cancer 2005;114:448-54. 10.1002/ijc.20710 15551359

[33] Wang K Li F Chen L Lai YM Zhang X Li HY Change in risk of breast cancer after receiving hormone replacement therapy by considering effect-modifiers: a systematic review and dose-response meta-analysis of prospective studies. Oncotarget 2017;8:81109-24. 10.18632/oncotarget.20154 29113371 PMC5655266

[34] Rossouw JE Anderson GL Prentice RL Writing Group for the Women’s Health Initiative Investigators Risks and benefits of estrogen plus progestin in healthy postmenopausal women: principal results From the Women’s Health Initiative randomized controlled trial. JAMA 2002;288:321-33. 10.1001/jama.288.3.321 12117397

[35] Collaborative Group on Hormonal Factors in Breast Cancer Breast cancer and hormone replacement therapy: collaborative reanalysis of data from 51 epidemiological studies of 52,705 women with breast cancer and 108,411 women without breast cancer. Lancet 1997;350:1047-59. 10.1016/S0140-6736(97)08233-0 10213546

[36] Feigelson HS Jonas CR Teras LR Thun MJ Calle EE Weight gain, body mass index, hormone replacement therapy, and postmenopausal breast cancer in a large prospective study. Cancer Epidemiol Biomarkers Prev 2004;13:220-4. 10.1158/1055-9965.EPI-03-0301 14973094

