# Supplementary material for: Use of hormone replacement therapy and risk of breast cancer: nested case-control studies using the QResearch and CPRD databases
Source: BMJ. 2020 Oct 28;371:m3873. doi: 10.1136/bmj.m3873 (PMC7592147; doi:10.1136/bmj.m3873)
Supplement: Supplementary file 1 — Supplementary information: Tables e1-e16 and figures e1-e7 [file viny053479.ww.pdf]

Use of hormone replacement therapy and risk of breast cancer: two nested case-control studies in primary care

Supplementary tables

eTable 1 Characteristics in cases and controls at least 1 years before the index date across the databases by exposure to HRT 3

eTable 2 **All cases and controls:** Duration of use for different hormones of HRT: number of cases and controls, unadjusted and adjusted odds ratios by database and combined results. 6

eTable 3 Other medications prescribed for menopausal women by database and combined analysis 9

eTable 4 **All cases and controls:** Duration of use for different doses of hormones and types of application by database 10

eTable 5 **All cases and controls:** Gap since the last use of different hormones of HRT by database. 14

eTable 6 **All cases and controls:** Duration of use for different hormones of HRT and tibolone in women with **recent** (**between >1 and <5 years** before the index date) and **past** (**5 years or more** before the index date)exposures, combined analysis 16

eTable 7 **All cases and controls:** Duration of use for different doses of hormones and types of application in women with **recent** (**between >1 and <5** years before the index date) and **past** (**5 years or more** before the index date)exposures, combined analysis 19

eTable 8 **All cases and controls:** Duration of use for different hormones of HRT and tibolone in women with **recent** (**between >1 and <2 years** before the index date) and **past** (**2 years or more** before the index date)exposures, combined analysis 23

eTable 9 Duration of use for different hormones of HRT and tibolone in women with **recent** (between >1 and <5 years) different exposures by age at the index date 27

eTable 10 Duration of use for different hormones of HRT and tibolone in women with **past** (5 years or more before the index date) different exposures by age at the index date 29

eTable 11 Duration of use for different hormones of HRT and tibolone in women with **recent** (between >1 and <5 years before the index date) different exposures by body-mass categories at 1years before the index date 32

eTable 12 Duration of use for different hormones of HRT and tibolone in women with **past** (5 years or more before the index date) different exposures by body-mass categories at 1 year before the index date 34

eTable 13 **All cases and controls with 10 years of records:** Duration of use for different hormones of HRT and tibolone in women with **recent** (**between >1 and <5 years** before the index date) and **past** (**5 years** or more before the index date) exposures, combined analysis 37

eTable 14 **All cases and controls aged 55 and older:** Duration of use for different hormones of HRT and tibolone in women with **recent** (**between >1 and <5 years** before the index date) and **past** (**5 years or more** before the index date)exposures, combined analysis 40

eTable 15 **All cases and controls:** Years of use since the first prescription, for different hormones of HRT, by database 42

eTable 16 Comparison our results with the recent meta-analysis (Lancet 2019) 45

Supplementary figures

eFigure 1 Gap since the last use of oestrogen only, oestrogen-progestogen and tibolone therapies 13
eFigure 3 Exposure to HRT by definition of recency 21
eFigure 4 Duration of exposure to oestrogen-only and oestrogen-progestogen therapies and risks in recent (last use within >1 and <2 years before the index date) and past (≥2 years) users 22
eFigure 5 Recent (between >1 and <5 years) use of different types of HRT in women of different age 26
eFigure 6 Recent (between >1 and <5 years) exposure to different types of HRT in women of different body mass index 31
eFigure 7 Sensitivity analyses: women with 10 years of records and women 55 years and older 36

eTable 1 Characteristics in cases and controls at least 1 years before the index date across the databases by exposure to HRT

	**No exposure**		**Oestrogen only**		**Combined therapy**	
	Cases; % (N)	Controls; % (N)	Cases; % (N)	Controls; % (N)	Cases; % (N)	Controls; % (N)
Total	64908	315107	8860	42799	24843	99592
**Age in years**						
age (mean, SE)	63.6 (9.0)	63.7 (8.9)	63.9 (7.2)	63.5 (7.2)	62.7 (6.8)	62.6 (6.9)
50 to 59	37.3 (24187)	36.4 (114734)	29.4 (2609)	31.3 (13382)	34.6 (8598)	35.0 (34808)
60 to 69	32.3 (20949)	33.0 (104036)	46.8 (4147)	47.2 (20211)	48.0 (11919)	48.0 (47819)
70 to 79	30.5 (19772)	30.6 (96337)	23.7 (2104)	21.5 (9206)	17.4 (4326)	17.0 (16965)
**Years of records (mean, SE)**	12.0 (5.6)	12.4 (5.5)	12.9 (5.7)	13.4 (5.5)	13.3 (5.6)	13.8 (5.5)
**Ethnicity**						
Recorded	69.2 (44902)	67.2 (211682)	71.7 (6349)	70.4 (30137)	72.0 (17879)	70.3 (70043)
White or not recorded	96.1 (62360)	95.5 (301061)	97.9 (8675)	97.6 (41763)	98.2 (24400)	97.7 (97315)
**Townsend quintile***						
Most affluent	24.6 (13311)	24.4 (63648)	26.7 (1997)	26.5 (9386)	27.1 (5703)	27.4 (22700)
2	23.6 (12798)	23.4 (60934)	24.8 (1854)	24.4 (8667)	25.1 (5284)	24.7 (20504)
3	21.2 (11477)	21.1 (55099)	20.2 (1511)	21.0 (7455)	20.9 (4389)	20.6 (17107)
4	17.8 (9645)	18.2 (47475)	17.0 (1268)	17.3 (6138)	16.3 (3426)	16.5 (13731)
Most deprived	12.7 (6888)	13.0 (33794)	11.4 (850)	10.8 (3822)	10.6 (2236)	10.8 (8946)
**Body mass index**						
recorded	82.3 (53402)	79.8 (251518)	87.7 (7770)	87.8 (37577)	88.0 (21852)	88.1 (87782)
mean (SE)	27.9 (5.7)	27.5 (5.7)	27.9 (5.4)	27.7 (5.4)	26.9 (5.2)	26.8 (5.2)
15 to 24 kg/m^2	29.0 (18807)	30.6 (96492)	29.3 (2599)	31.0 (13280)	36.2 (8998)	37.0 (36816)
25 to 29 kg/m^2	28.3 (18375)	27.0 (85234)	31.6 (2803)	31.6 (13529)	30.6 (7600)	30.7 (30615)
30 kg/m^2 and over	25.0 (16220)	22.1 (69792)	26.7 (2368)	25.2 (10768)	21.1 (5254)	20.4 (20351)
**Smoking**						
recorded	89.4 (58054)	86.9 (273892)	92.4 (8189)	92.4 (39561)	92.8 (23058)	93.0 (92625)

none	49.2 (31962)	48.9 (154121)	45.6 (4038)	47.1 (20147)	43.6 (10826)	44.8 (44631)
ex	26.8 (17399)	24.8 (78050)	31.3 (2772)	29.7 (12708)	32.3 (8029)	31.3 (31220)
light (1-9 cigarettes/day)	6.4 (4186)	6.5 (20567)	7.4 (659)	7.3 (3129)	8.0 (1988)	8.0 (7942)
moderate (10-19)	4.3 (2801)	4.2 (13266)	4.9 (435)	5.1 (2164)	5.3 (1324)	5.3 (5274)
heavy (≥20)	2.6 (1706)	2.5 (7888)	3.2 (285)	3.3 (1413)	3.6 (891)	3.6 (3558)
**Alcohol**						
recorded	80.6 (52328)	78.1 (246231)	85.0 (7534)	85.1 (36425)	86.3 (21439)	86.3 (85993)
none	24.0 (15585)	24.6 (77646)	23.2 (2052)	24.5 (10471)	18.9 (4688)	20.4 (20298)
ex-use	5.6 (3624)	5.4 (17134)	7.3 (648)	6.9 (2952)	6.6 (1638)	6.5 (6487)
trivial (<1units/day)	30.2 (19604)	29.5 (92911)	32.9 (2914)	33.5 (14317)	33.5 (8333)	34.3 (34198)
light (1-2)	12.6 (8193)	11.6 (36502)	13.7 (1212)	12.9 (5509)	16.0 (3978)	15.3 (15287)
moderate (3-6)	7.3 (4723)	6.3 (19816)	7.2 (636)	6.6 (2841)	10.2 (2530)	8.7 (8692)
heavy (7-9)	0.6 (384)	0.5 (1495)	0.6 (51)	0.5 (224)	0.8 (187)	0.7 (713)
very heavy (≥10)	0.3 (215)	0.2 (727)	0.2 (21)	0.3 (111)	0.3 (85)	0.3 (318)
**History of other cancers**						
any cancer	3.0 (1919)	2.6 (8053)	4.5 (400)	3.7 (1574)	2.9 (726)	2.5 (2483)
blood cancer	0.4 (249)	0.4 (1118)	0.5 (41)	0.4 (176)	0.6 (142)	0.4 (397)
cervical cancer	0.1 (93)	0.2 (558)	0.7 (64)	0.8 (326)	0.1 (30)	0.2 (168)
colorectal	0.6 (358)	0.5 (1550)	0.6 (54)	0.5 (197)	0.4 (109)	0.4 (431)
lung cancer	0.1 (60)	0.1 (337)	0.2 (18)	0.2 (69)	0.1 (34)	0.1 (108)
melanoma	0.5 (305)	0.4 (1196)	0.8 (69)	0.5 (217)	0.5 (134)	0.5 (477)
ovarian cancer	0.2 (126)	0.2 (479)	0.5 (43)	0.5 (195)	0.2 (44)	0.1 (137)
uterine cancer	0.3 (168)	0.2 (698)	0.2 (17)	0.2 (75)	0.2 (46)	0.1 (142)
**Chronic conditions**						
benign breast disease	8.0 (5225)	5.0 (15776)	10.9 (965)	7.7 (3281)	10.4 (2575)	6.9 (6916)
diabetes	7.6 (4962)	7.1 (22309)	7.2 (639)	6.9 (2971)	5.1 (1268)	5.1 (5087)
mental health disorder	0.9 (578)	0.8 (2373)	0.8 (73)	0.7 (316)	0.9 (236)	0.9 (878)
osteoporosis	3.2 (2065)	3.6 (11373)	4.4 (389)	4.9 (2113)	4.4 (1089)	5.2 (5154)

**Other characteristics**						
Age at HRT start (mean, SD)	n/a	n/a	52.4 (6.4)	51.7 (6.5)	51.6 (5.4)	51.2 (5.4)
younger than 44	n/a	n/a	8.7 (773)	11.2 (4801)	6.9 (1709)	8.1 (8033)
45 to 49	n/a	n/a	25.5 (2255)	27.4 (11707)	28.8 (7151)	30.5 (30384)
50 to 54	n/a	n/a	33.4 (2960)	32.1 (13724)	38.9 (9670)	38.4 (38249)
55 to 59	n/a	n/a	18.1 (1607)	17.4 (7459)	17.3 (4291)	15.9 (15839)
60 and older	n/a	n/a	14.3 (1265)	11.9 (5108)	8.1 (2022)	7.1 (7087)
early menopause	3.0 (1919)	3.2 (10194)	22.0 (1947)	23.6 (10103)	2.4 (596)	3.1 (3098)
late menopause	2.6 (1667)	2.3 (7154)	4.3 (381)	4.2 (1797)	4.7 (1179)	4.7 (4642)
menopausal symptoms	10.4 (6735)	10.3 (32310)	25.8 (2283)	26.0 (11121)	32.7 (8126)	34.1 (33951)
mammography scans#	33.8 (21971)	33.4 (105191)	47.9 (4247)	46.6 (19948)	49.1 (12194)	47.5 (47307)
MRI/CT scans#	4.5 (2899)	4.3 (13452)	7.6 (674)	7.6 (3262)	5.8 (1435)	6.1 (6055)
oophorectomy/hysterectomy	15.2 (9865)	14.9 (47049)	88.6 (7854)	89.8 (38425)	12.0 (2982)	12.6 (12521)
**Family history**						
any cancer	7.6 (4956)	5.9 (18641)	8.1 (714)	6.8 (2928)	8.3 (2053)	6.8 (6818)
breast cancer	3.8 (2457)	2.3 (7349)	4.5 (400)	3.0 (1275)	4.3 (1070)	2.9 (2933)
cervical cancer	0.0 (30)	0.0 (106)	0.0 (2)	0.0 (16)	0.0 (9)	0.0 (41)
osteoporosis	0.6 (396)	0.5 (1730)	1.0 (91)	1.2 (502)	1.5 (381)	1.5 (1525)
ovarian cancer	0.2 (101)	0.1 (420)	0.2 (16)	0.2 (76)	0.2 (49)	0.2 (154)
uterine cancer	0.0 (32)	0.0 (148)	0.1 (6)	0.0 (20)	0.1 (14)	0.0 (33)
**Other medications before the index date**						
aspirin	15.9 (10323)	15.8 (49685)	20.9 (1850)	20.4 (8722)	14.6 (3636)	15.4 (15342)
non-steroidal anti-inflammatory drugs	58.2 (37795)	57.1 (179988)	76.3 (6761)	77.3 (33064)	72.8 (18078)	74.2 (73892)
contraceptive drugs	14.2 (9191)	13.1 (41149)	6.5 (574)	6.9 (2949)	12.5 (3110)	13.2 (13126)
tamoxifen	1.1 (684)	0.1 (451)	0.5 (43)	0.2 (92)	0.4 (98)	0.1 (128)
raloxifene	0.2 (138)	0.2 (779)	0.6 (54)	0.8 (361)	0.8 (200)	1.0 (996)

*based on cases and controls with available Townsend data; n/a – not applicable

based on HES and GP data for QResearch and on GP data for CPRD

eTable 2 ***All cases and controls:*** *Duration of use for different hormones of HRT: number of cases and controls, unadjusted and adjusted odds ratios by database and combined results.*

	QResearch			CPRD			Combined	
	N of cases; controls	Unadjusted odds ratio (95% confidence interval)	Adjusted odds ratio[#] (95% confidence interval)	N of cases; controls	Unadjusted odds ratio (95% confidence interval)	Adjusted odds ratio[#] (95% confidence interval)	Adjusted odds ratio[#] (95% confidence interval)	P-value*
Overall use								
HRT	20494; 83576	1.19 (1.17 to 1.22)[β]	1.21 (1.19 to 1.24)[β]	13209; 58815	1.17 (1.14 to 1.20)[β]	1.21 (1.18 to 1.25)[β]	1.21 (1.19 to 1.23)	<0.001
Oestrogen only	5126; 24302	1.03 (1.00 to 1.06)	1.06 (1.02 to 1.10)[α]	3734; 18497	1.05 (1.01 to 1.09)	1.07 (1.02 to 1.12)[α]	1.06 (1.03 to 1.10)	<0.001
Combined therapy	15368; 59274	1.27 (1.24 to 1.29)[β]	1.26 (1.23 to 1.29)[β]	9475; 40318	1.23 (1.19 to 1.26)[β]	1.26 (1.23 to 1.30)[β]	1.26 (1.24 to 1.29)	<0.001
Duration of Exposure								
**HRT**								
<1 year	4002; 19353	0.99 (0.96 to 1.03)	1.01 (0.98 to 1.05)	3276; 16114	1.05 (1.01 to 1.10)	1.08 (1.04 to 1.13)[β]	1.04 (1.01 to 1.07)	0.004
1-2 years	4367; 19337	1.09 (1.06 to 1.13)[β]	1.11 (1.07 to 1.16)[β]	3258; 14625	1.16 (1.11 to 1.21)[β]	1.19 (1.14 to 1.25)[β]	1.15 (1.12 to 1.18)	<0.001
3-4 years	3734; 14793	1.23 (1.19 to 1.28)[β]	1.26 (1.21 to 1.31)[β]	2243; 9852	1.19 (1.13 to 1.25)[β]	1.23 (1.17 to 1.29)[β]	1.25 (1.21 to 1.29)	<0.001
5-9 years	6188; 23180	1.32 (1.28 to 1.37)[β]	1.37 (1.32 to 1.41)[β]	3315; 13909	1.25 (1.20 to 1.31)[β]	1.34 (1.28 to 1.40)[β]	1.36 (1.32 to 1.39)	<0.001
10+ years	2203; 6913	1.59 (1.51 to 1.67)[β]	1.67 (1.58 to 1.76)[β]	1117; 4315	1.37 (1.28 to 1.47)[β]	1.52 (1.42 to 1.64)[β]	1.62 (1.55 to 1.69)	<0.001
OESTROGEN ONLY								
<1 year	956; 4475	1.04 (0.96 to 1.11)	1.07 (0.99 to 1.15)	914; 4370	1.08 (1.00 to 1.16)	1.09 (1.01 to 1.18)	1.08 (1.02 to 1.14)	0.005
1-2 years	987; 4981	0.97 (0.90 to 1.04)	1.00 (0.93 to 1.08)	859; 4282	1.04 (0.97 to 1.12)	1.05 (0.97 to 1.14)	1.02 (0.97 to 1.08)	0.4
3-4 years	924; 4236	1.07 (0.99 to 1.15)	1.11 (1.03 to 1.20)[α]	617; 3062	1.05 (0.96 to 1.15)	1.07 (0.97 to 1.17)	1.09 (1.03 to 1.16)	0.003
5-9 years	1606; 7706	1.03 (0.98 to 1.09)	1.08 (1.02 to 1.15)	988; 4936	1.05 (0.98 to 1.13)	1.11 (1.03 to 1.20)[α]	1.09 (1.04 to 1.15)	<0.001
10+ years	653; 2904	1.12 (1.03 to 1.22)	1.19 (1.09 to 1.31)[β]	356; 1847	1.02 (0.91 to 1.14)	1.12 (0.99 to 1.26)	1.17 (1.08 to 1.25)	<0.001
**Conjugated equine oestrogen**								
<1 year	710; 3631	0.94 (0.86 to 1.02)	0.96 (0.88 to 1.04)	592; 3094	0.98 (0.89 to 1.07)	0.99 (0.90 to 1.09)	0.97 (0.91 to 1.03)	0.4
1-2 years	531; 2808	0.91 (0.82 to 1.00)	0.94 (0.85 to 1.03)	435; 2049	1.08 (0.98 to 1.21)	1.08 (0.97 to 1.20)	1.00 (0.93 to 1.07)	0.9
3-4 years	467; 2077	1.09 (0.98 to 1.21)	1.12 (1.01 to 1.24)	283; 1385	1.05 (0.92 to 1.19)	1.06 (0.92 to 1.21)	1.09 (1.01 to 1.19)	0.03
5-9 years	730; 3561	1.01 (0.93 to 1.10)	1.05 (0.96 to 1.14)	462; 2234	1.07 (0.97 to 1.19)	1.12 (1.01 to 1.25)	1.07 (1.01 to 1.15)	0.03

10+ years	242; 1174	1.02 (0.89 to 1.18)	1.07 (0.93 to 1.23)	156; 860	0.94 (0.79 to 1.12)	1.02 (0.85 to 1.21)	1.05 (0.94 to 1.17)	0.4
**Estradiol**								
<1 year	835; 4144	0.98 (0.91 to 1.06)	0.99 (0.91 to 1.07)	849; 4268	1.02 (0.94 to 1.10)	1.01 (0.93 to 1.10)	1.00 (0.95 to 1.06)	1
1-2 years	762; 3685	1.02 (0.94 to 1.10)	1.02 (0.94 to 1.11)	670; 3328	1.03 (0.95 to 1.13)	1.03 (0.94 to 1.13)	1.03 (0.97 to 1.09)	0.4
3-4 years	564; 2675	1.04 (0.94 to 1.14)	1.05 (0.96 to 1.16)	388; 2033	0.98 (0.88 to 1.10)	0.98 (0.88 to 1.10)	1.02 (0.95 to 1.10)	0.5
5-9 years	869; 4089	1.06 (0.98 to 1.14)	1.08 (1.00 to 1.16)	523; 2567	1.05 (0.96 to 1.16)	1.09 (0.99 to 1.21)	1.08 (1.02 to 1.15)	0.01
10+ years	343; 1383	1.24 (1.10 to 1.40)$^{\beta}$	1.29 (1.14 to 1.46)$^{\beta}$	146; 755	1.01 (0.84 to 1.21)	1.08 (0.90 to 1.30)	1.22 (1.10 to 1.35)	<0.001
OESTROGEN COMBINED with								
**any progestogen**								
<1 year	3050; 14908	0.99 (0.95 to 1.03)	0.99 (0.95 to 1.03)	2362; 11744	1.05 (1.00 to 1.10)	1.07 (1.02 to 1.12)$^{\alpha}$	1.02 (0.99 to 1.06)	0.1
1-2 years	3380; 14362	1.14 (1.10 to 1.19)$^{\beta}$	1.14 (1.10 to 1.19)$^{\beta}$	2399; 10343	1.21 (1.15 to 1.27)$^{\beta}$	1.24 (1.18 to 1.30)$^{\beta}$	1.18 (1.15 to 1.22)	<0.001
3-4 years	2811; 10559	1.31 (1.25 to 1.36)$^{\beta}$	1.31 (1.25 to 1.37)$^{\beta}$	1626; 6790	1.26 (1.19 to 1.33)$^{\beta}$	1.29 (1.21 to 1.36)$^{\beta}$	1.30 (1.25 to 1.35)	<0.001
5-9 years	4583; 15475	1.48 (1.42 to 1.53)$^{\beta}$	1.49 (1.43 to 1.54)$^{\beta}$	2327; 8973	1.37 (1.31 to 1.44)$^{\beta}$	1.44 (1.36 to 1.51)$^{\beta}$	1.47 (1.42 to 1.51)	<0.001
10+ years	1550; 4010	1.94 (1.82 to 2.06)$^{\beta}$	1.96 (1.84 to 2.09)$^{\beta}$	761; 2468	1.65 (1.51 to 1.79)$^{\beta}$	1.77 (1.62 to 1.93)$^{\beta}$	1.89 (1.80 to 1.99)	<0.001
**Medroxyprogesterone**								
<1 year	1907; 7851	1.01 (0.96 to 1.06)	1.01 (0.96 to 1.07)	1198; 5166	1.07 (1.00 to 1.14)	1.08 (1.00 to 1.15)	1.04 (0.99 to 1.08)	0.1
1-2 years	1282; 4806	1.12 (1.05 to 1.19)$^{\beta}$	1.11 (1.04 to 1.19)$^{\alpha}$	766; 2893	1.23 (1.13 to 1.33)$^{\beta}$	1.24 (1.14 to 1.34)$^{\beta}$	1.16 (1.10 to 1.22)	<0.001
3-4 years	784; 2504	1.35 (1.24 to 1.47)$^{\beta}$	1.36 (1.25 to 1.48)$^{\beta}$	385; 1379	1.31 (1.17 to 1.47)$^{\beta}$	1.32 (1.17 to 1.48)$^{\beta}$	1.35 (1.26 to 1.44)	<0.001
5-9 years	617; 1836	1.50 (1.36 to 1.65)$^{\beta}$	1.49 (1.35 to 1.64)$^{\beta}$	268; 1009	1.27 (1.11 to 1.46)$^{\beta}$	1.31 (1.14 to 1.51)$^{\beta}$	1.43 (1.32 to 1.55)	<0.001
10+ years	100; 156	2.90 (2.24 to 3.74)$^{\beta}$	2.90 (2.24 to 3.76)$^{\beta}$	29; 83	1.71 (1.12 to 2.62)	1.78 (1.16 to 2.74)$^{\alpha}$	2.55 (2.04 to 3.18)	<0.001
Levonorgestrel								
<1 year	2911; 12407	1.02 (0.97 to 1.07)	1.02 (0.97 to 1.07)	1863; 8809	1.00 (0.95 to 1.06)	1.02 (0.96 to 1.08)	1.02 (0.98 to 1.06)	0.3
1-2 years	2321; 8629	1.17 (1.11 to 1.23)$^{\beta}$	1.16 (1.11 to 1.22)$^{\beta}$	1267; 5513	1.07 (1.01 to 1.15)	1.09 (1.02 to 1.16)	1.13 (1.09 to 1.18)	<0.001
3-4 years	1386; 4924	1.24 (1.17 to 1.32)$^{\beta}$	1.23 (1.16 to 1.31)$^{\beta}$	767; 2952	1.22 (1.12 to 1.32)$^{\beta}$	1.26 (1.15 to 1.37)$^{\beta}$	1.24 (1.18 to 1.30)	<0.001
5-9 years	1595; 4778	1.53 (1.44 to 1.62)$^{\beta}$	1.52 (1.44 to 1.62)$^{\beta}$	907; 3256	1.32 (1.22 to 1.42)$^{\beta}$	1.38 (1.27 to 1.49)$^{\beta}$	1.47 (1.40 to 1.54)	<0.001
10+ years	261; 666	1.86 (1.61 to 2.16)$^{\beta}$	1.89 (1.63 to 2.19)$^{\beta}$	162; 519	1.54 (1.29 to 1.85)$^{\beta}$	1.68 (1.40 to 2.01)$^{\beta}$	1.80 (1.61 to 2.02)	<0.001
**Norethisterone**								
<1 year	3220; 13971	1.00 (0.96 to 1.04)	1.00 (0.96 to 1.05)	2475; 11566	1.03 (0.98 to 1.08)	1.04 (0.99 to 1.09)	1.02 (0.99 to 1.05)	0.3
1-2 years	2398; 9538	1.11 (1.06 to 1.16)$^{\beta}$	1.11 (1.06 to 1.17)$^{\beta}$	1544; 6281	1.20 (1.13 to 1.27)$^{\beta}$	1.22 (1.15 to 1.29)$^{\beta}$	1.15 (1.11 to 1.20)	<0.001

3-4 years	1494; 5258	1.29 (1.21 to 1.37)[β]	1.29 (1.21 to 1.37)[β]	781; 3153	1.22 (1.13 to 1.33)[β]	1.25 (1.15 to 1.36)[β]	1.27 (1.21 to 1.34)	<0.001
5-9 years	1686; 5346	1.47 (1.38 to 1.55)[β]	1.48 (1.40 to 1.57)[β]	761; 2561	1.49 (1.37 to 1.62)[β]	1.56 (1.43 to 1.70)[β]	1.51 (1.43 to 1.58)	<0.001
10+ years	353; 778	2.16 (1.90 to 2.45)[β]	2.21 (1.94 to 2.51)[β]	122; 370	1.67 (1.36 to 2.06)[β]	1.77 (1.44 to 2.18)[β]	2.08 (1.86 to 2.32)	<0.001
**Dydrogesterone**								
<1 year	932; 3745	0.99 (0.92 to 1.07)	0.99 (0.92 to 1.07)	524; 2406	0.96 (0.87 to 1.06)	0.97 (0.88 to 1.07)	0.98 (0.92 to 1.04)	0.6
1-2 years	469; 1913	1.00 (0.90 to 1.11)	1.00 (0.90 to 1.11)	228; 1141	0.90 (0.78 to 1.04)	0.91 (0.78 to 1.05)	0.96 (0.89 to 1.05)	0.4
3-4 years	165; 788	0.87 (0.74 to 1.04)	0.85 (0.72 to 1.01)	74; 381	0.89 (0.70 to 1.15)	0.93 (0.72 to 1.19)	0.88 (0.76 to 1.01)	0.07
5-9 years	140; 490	1.22 (1.01 to 1.48)	1.23 (1.02 to 1.49)	49; 289	0.80 (0.59 to 1.08)	0.85 (0.62 to 1.15)	1.11 (0.94 to 1.31)	0.2
10+ years	23; 66	1.51 (0.93 to 2.45)	1.51 (0.93 to 2.45)	10; 27	1.81 (0.87 to 3.73)	2.07 (1.00 to 4.29)	1.66 (1.11 to 2.49)	0.01
TIBOLONE								
<1 year	1260; 5373	0.97 (0.91 to 1.04)	0.98 (0.92 to 1.04)	923; 4512	0.93 (0.86 to 1.00)	0.93 (0.87 to 1.01)	0.96 (0.91 to 1.01)	0.1
1-2 years	591; 2494	1.01 (0.92 to 1.10)	1.00 (0.91 to 1.10)	422; 1763	1.10 (0.99 to 1.22)	1.08 (0.97 to 1.20)	1.03 (0.96 to 1.11)	0.4
3-4 years	272; 1233	0.98 (0.86 to 1.12)	0.96 (0.84 to 1.10)	207; 852	1.14 (0.98 to 1.33)	1.15 (0.98 to 1.34)	1.04 (0.94 to 1.15)	0.5
5-9 years	326; 1242	1.19 (1.06 to 1.35)[α]	1.18 (1.05 to 1.34)[α]	205; 871	1.14 (0.98 to 1.33)	1.15 (0.99 to 1.35)	1.17 (1.06 to 1.29)	0.001
10+ years	66; 220	1.40 (1.06 to 1.85)	1.42 (1.08 to 1.88)	36; 167	1.08 (0.75 to 1.55)	1.13 (0.78 to 1.63)	1.31 (1.05 to 1.63)	0.02

[#]Odds ratios are with reference to never-users and based on cases and controls matched by age and practice and adjusted for smoking status, body mass index, family history of cancer, medical conditions and events, other medications and contraceptive drugs.

[α] P-value<0.01; [β] P-value<0.001

eTable 3 Other medications prescribed for menopausal women by database and combined analysis

	QResearch			CPRD			Combined	
	N of cases; controls	Unadjusted odds ratio (95% confidence interval)	Adjusted odds ratio# (95% confidence interval)	N of cases; controls	Unadjusted odds ratio (95% confidence interval)	Adjusted odds ratio# (95% confidence interval)	adjusted odds ratio# (95% confidence interval)	
**Oestrogen cream**								
<1 year	4381; 20620	0.96 (0.92 to 0.99)	0.96 (0.93 to 0.99)	1802; 9163	0.95 (0.90 to 1.00)	0.94 (0.89 to 1.00)	0.95 (0.93 to 0.98)	0.003
1-2 years	595; 2934	0.92 (0.84 to 1.01)	0.93 (0.85 to 1.02)	189; 951	0.95 (0.81 to 1.12)	0.92 (0.79 to 1.09)	0.93 (0.86 to 1.00)	0.06
3+ years	233; 1045	1.03 (0.89 to 1.18)	1.03 (0.89 to 1.19)	51; 269	0.91 (0.68 to 1.23)	0.93 (0.69 to 1.26)	1.01 (0.89 to 1.15)	0.9
**Vaginal preparations**								
<1 year	2485; 11591	0.96 (0.91 to 1.00)	0.96 (0.92 to 1.01)	1605; 8366	0.93 (0.88 to 0.98)[α]	0.92 (0.87 to 0.97)[α]	0.94 (0.91 to 0.98)	0.002
1-2 years	416; 1926	0.97 (0.87 to 1.08)	0.96 (0.86 to 1.07)	247; 1240	0.95 (0.83 to 1.09)	0.93 (0.81 to 1.07)	0.95 (0.87 to 1.04)	0.2
3+ years	141; 720	0.87 (0.73 to 1.05)	0.89 (0.74 to 1.07)	113; 445	1.21 (0.98 to 1.49)	1.15 (0.93 to 1.43)	0.99 (0.87 to 1.14)	0.9

#Odds ratios are with reference to never-users and based on cases and controls matched by age and practice and adjusted for smoking status, body mass index, family history of cancer, medical conditions and events, other medications and contraceptive drugs.

[α] P-value<0.01; [β] P-value<0.001

eTable 4 ***All cases and controls:*** *Duration of use for different doses of hormones and types of application by database*

	QResearch		CPRD		Combined analysis	
	N of cases; controls	Adjusted odds ratio[#] (95% confidence interval)	N of cases; controls	Adjusted odds ratio[#] (95% confidence interval)	Combined odds ratio (95% confidence interval)	P-value
OESTROGEN ONLY						
**Conjugated equine oestrogen**						
≤0.625mg						
<1 year	580; 2841	1.01 (0.92 to 1.11)	411; 2282	0.94 (0.84 to 1.05)	0.98 (0.91 to 1.05)	0.5
1-2 years	394; 2054	0.97 (0.87 to 1.08)	301; 1390	1.12 (0.98 to 1.28)	1.03 (0.95 to 1.12)	0.5
3-4 years	331; 1468	1.14 (1.01 to 1.28)	198; 892	1.16 (0.99 to 1.36)	1.15 (1.04 to 1.26)	0.006
5-9 years	493; 2446	1.04 (0.94 to 1.15)	307; 1431	1.17 (1.03 to 1.33)	1.09 (1.01 to 1.18)	0.03
10+ years	125; 718	0.90 (0.74 to 1.09)	100; 510	1.10 (0.88 to 1.37)	0.98 (0.85 to 1.14)	0.8
>0.625mg						
<1 year	130; 790	0.80 (0.66 to 0.96)	181; 812	1.17 (0.99 to 1.38)	0.99 (0.87 to 1.12)	0.9
1-2 years	137; 754	0.90 (0.75 to 1.08)	134; 659	1.03 (0.85 to 1.25)	0.96 (0.84 to 1.10)	0.6
3-4 years	136; 609	1.11 (0.92 to 1.34)	85; 493	0.92 (0.73 to 1.17)	1.03 (0.89 to 1.20)	0.7
5-9 years	237; 1115	1.09 (0.95 to 1.26)	155; 803	1.08 (0.91 to 1.29)	1.09 (0.97 to 1.22)	0.1
10+ years	117; 456	1.37 (1.11 to 1.69)[α]	56; 350	0.93 (0.70 to 1.24)	1.20 (1.01 to 1.42)	0.03
**Estradiol**						
≤1mg						
<1 year	725; 3552	1.01 (0.93 to 1.10)	727; 3558	1.06 (0.97 to 1.15)	1.04 (0.98 to 1.10)	0.3
1-2 years	611; 2915	1.06 (0.96 to 1.16)	511; 2584	1.04 (0.94 to 1.15)	1.05 (0.98 to 1.12)	0.2
3-4 years	422; 2012	1.06 (0.95 to 1.18)	295; 1477	1.05 (0.93 to 1.20)	1.06 (0.97 to 1.15)	0.2
5-9 years	648; 3041	1.10 (1.00 to 1.20)	377; 1800	1.14 (1.01 to 1.28)	1.11 (1.03 to 1.19)	0.004
10+ years	250; 957	1.39 (1.20 to 1.60)[β]	86; 540	0.90 (0.72 to 1.14)	1.23 (1.09 to 1.39)	<0.001

>1mg						
<1 year	110; 592	0.89 (0.72 to 1.09)	122; 710	0.89 (0.73 to 1.08)	0.89 (0.77 to 1.02)	0.1
1-2 years	151; 768	0.96 (0.80 to 1.15)	159; 744	1.07 (0.90 to 1.28)	1.01 (0.89 to 1.15)	0.8
3-4 years	142; 663	1.09 (0.91 to 1.31)	93; 556	0.87 (0.70 to 1.09)	1.00 (0.86 to 1.15)	1
5-9 years	221; 1048	1.09 (0.94 to 1.26)	146; 767	1.06 (0.88 to 1.27)	1.08 (0.96 to 1.21)	0.2
10+ years	93; 426	1.16 (0.93 to 1.46)	60; 215	1.58 (1.18 to 2.12)[α]	1.31 (1.09 to 1.56)	0.004
oral						
<1 year	556; 2699	1.00 (0.91 to 1.11)	484; 2353	1.07 (0.97 to 1.19)	1.03 (0.96 to 1.11)	0.3
1-2 years	365; 1730	1.02 (0.91 to 1.15)	292; 1374	1.08 (0.94 to 1.23)	1.05 (0.96 to 1.14)	0.3
3-4 years	220; 1115	0.99 (0.85 to 1.15)	158; 835	0.97 (0.82 to 1.16)	0.98 (0.88 to 1.10)	0.8
5-9 years	293; 1451	1.01 (0.89 to 1.15)	171; 976	0.94 (0.79 to 1.11)	0.98 (0.89 to 1.09)	0.7
10+ years	80; 371	1.13 (0.88 to 1.44)	60; 243	1.35 (1.01 to 1.81)	1.22 (1.01 to 1.47)	0.04
transdermal						
<1 year	634; 3246	0.96 (0.88 to 1.05)	719; 3710	0.97 (0.89 to 1.06)	0.97 (0.91 to 1.03)	0.3
1-2 years	546; 2627	1.03 (0.94 to 1.14)	450; 2379	0.97 (0.87 to 1.08)	1.00 (0.93 to 1.08)	0.9
3-4 years	361; 1679	1.08 (0.96 to 1.21)	242; 1234	1.01 (0.87 to 1.16)	1.05 (0.96 to 1.15)	0.3
5-9 years	571; 2540	1.14 (1.04 to 1.25)[α]	338; 1508	1.20 (1.06 to 1.35)[α]	1.16 (1.08 to 1.25)	<0.001
10+ years	238; 919	1.35 (1.16 to 1.56)[β]	73; 460	0.88 (0.69 to 1.14)	1.21 (1.07 to 1.38)	0.003
OESTROGEN COMBINED with						
**Levonorgestrel**						
oral						
<1 year	2796; 12008	1.01 (0.97 to 1.06)	1796; 8612	1.01 (0.95 to 1.07)	1.01 (0.98 to 1.05)	0.6
1-2 years	2170; 8158	1.15 (1.10 to 1.21)[β]	1225; 5321	1.09 (1.02 to 1.17)	1.13 (1.08 to 1.18)	<0.001
3-4 years	1292; 4549	1.25 (1.17 to 1.34)[β]	715; 2771	1.25 (1.15 to 1.37)[β]	1.25 (1.19 to 1.32)	<0.001
5-9 years	1531; 4590	1.53 (1.43 to 1.62)[β]	878; 3128	1.39 (1.28 to 1.50)[β]	1.47 (1.40 to 1.55)	<0.001
10+ years	252; 647	1.88 (1.62 to 2.18)[β]	159; 504	1.70 (1.41 to 2.04)[β]	1.80 (1.61 to 2.02)	<0.001
transdermal						
<1 year	212; 835	1.03 (0.88 to 1.21)	94; 341	1.26 (1.00 to 1.59)	1.10 (0.96 to 1.25)	0.2
1-2 years	98; 273	1.47 (1.16 to 1.86)[α]	13; 66	0.93 (0.51 to 1.69)	1.38 (1.11 to 1.72)	0.004

3 years or more	42; 124	1.45 (1.01 to 2.07)	6; 17	1.75 (0.68 to 4.47)	1.48 (1.06 to 2.07)	0.02
intra-uterine device	231; 726	1.21 (1.04 to 1.41)	141; 559	1.11 (0.91 to 1.34)	1.17 (1.04 to 1.32)	0.01
**Norethisterone**						
oral						
<1 year	3019; 13097	0.99 (0.95 to 1.03)	2231; 10436	1.03 (0.97 to 1.08)	1.00 (0.97 to 1.04)	0.8
1-2 years	2000; 7626	1.15 (1.09 to 1.21)[β]	1268; 5158	1.20 (1.12 to 1.28)[β]	1.17 (1.12 to 1.22)	<0.001
3-4 years	1221; 4248	1.29 (1.21 to 1.38)[β]	675; 2684	1.27 (1.16 to 1.38)[β]	1.28 (1.21 to 1.35)	<0.001
5-9 years	1324; 3975	1.56 (1.46 to 1.67)[β]	672; 2184	1.60 (1.46 to 1.76)[β]	1.58 (1.49 to 1.66)	<0.001
10+ years	240; 514	2.23 (1.91 to 2.61)[β]	101; 306	1.77 (1.40 to 2.23)[β]	2.07 (1.82 to 2.36)	<0.001
transdermal						
<1 year	1160; 4686	1.01 (0.94 to 1.08)	821; 3944	0.95 (0.88 to 1.03)	0.99 (0.94 to 1.04)	0.6
1-2 years	689; 2860	1.01 (0.93 to 1.11)	333; 1330	1.19 (1.05 to 1.35)[α]	1.07 (1.00 to 1.15)	0.07
3-4 years	283; 1085	1.14 (0.99 to 1.30)	107; 436	1.17 (0.94 to 1.45)	1.15 (1.02 to 1.28)	0.02
5-9 years	309; 1107	1.28 (1.12 to 1.45)[β]	77; 286	1.39 (1.07 to 1.80)	1.30 (1.16 to 1.46)	<0.001
10+ years	83; 181	2.24 (1.72 to 2.92)[β]	6; 31	0.92 (0.38 to 2.23)	2.09 (1.62 to 2.69)	<0.001

[#]Odds ratios are with reference to never-users and based on cases and controls matched by age and practice and adjusted for smoking status, body mass index, family history of cancer, medical conditions and events, other medications and contraceptive drugs.

[α] P-value<0.01; [β] P-value<0.001

eFigure 1 Gap since the last use of oestrogen only, oestrogen-progestogen and tibolone therapies

	Years since the last use	Cases; controls	Odds ratio (95% CI)
**Oestrogen only**			
**Conjugated equine oestrogen**			
	13-23 months	1394; 6372	1.14 (1.07 to 1.21)
	2-4 years	717; 3680	0.97 (0.89 to 1.05)
	5-9 years	1157; 6092	0.95 (0.89 to 1.02)
	>10 years	1340; 6729	0.99 (0.93 to 1.05)
**Estradiol**			
	13-23 months	2209; 9657	1.18 (1.13 to 1.25)
	2-4 years	983; 5180	0.95 (0.89 to 1.02)
	5-9 years	1490; 7587	0.98 (0.92 to 1.04)
	>10 years	1267; 6503	0.95 (0.89 to 1.02)
**Oestrogen and progestogen**			
**Medroxyprogesterone acetate**			
	13-23 months	2316; 6307	1.64 (1.56 to 1.72)
	2-4 years	1404; 5505	1.11 (1.04 to 1.18)
	5-9 years	1986; 8545	1.04 (0.98 to 1.09)
	>10 years	1630; 7326	1.01 (0.96 to 1.07)
**Levonorgestrel**			
	13-23 months	2475; 7421	1.53 (1.46 to 1.61)
	2-4 years	2133; 7737	1.16 (1.10 to 1.22)
	5-9 years	3796; 15015	1.13 (1.08 to 1.17)
	>10 years	5036; 22280	1.05 (1.01 to 1.09)
**Norethisterone acetate**			
	13-23 months	4756; 13819	1.63 (1.57 to 1.69)
	2-4 years	2613; 11445	1.03 (0.98 to 1.07)
	5-9 years	3909; 17234	1.04 (1.00 to 1.08)
	>10 years	3556; 16324	1.01 (0.97 to 1.05)
**Dydrogesterone**			
	13-23 months	594; 2221	1.10 (1.01 to 1.21)
	2-4 years	507; 2228	0.90 (0.81 to 0.99)
	5-9 years	782; 3525	0.93 (0.85 to 1.00)
	>10 years	731; 3272	0.96 (0.89 to 1.05)

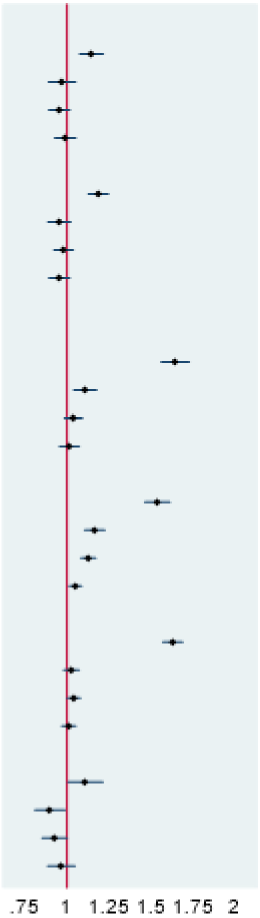

Odds ratios are with reference to never-users and adjusted for smoking, alcohol consumption, Townsend quintile (QResearch only), body mass index, ethnicity, history of other cancers, oophorectomy/hysterectomy, records of menopause, scans, comorbidities, other medications, years of data. Cases are matched to controls by age, general practice and index date.

eTable 5 ***All cases and controls:*** *Gap since the last use of different hormones of HRT by database.*

	QResearch		CPRD		Combined analysis	
	N of cases; controls	Adjusted odds ratio# (95% confidence interval)	N of cases; controls	Adjusted odds ratio# (95% confidence interval)	Combined odds ratio (95% confidence interval)	P-value
**HRT**						
13-23 months	8304; 27776	1.53 (1.48 to 1.58)$^{\beta}$	5159; 17196	1.70 (1.63 to 1.76)$^{\beta}$	1.59 (1.55 to 1.63)	<0.001
2-4 years	3270; 15451	1.05 (1.00 to 1.09)	2146; 10508	1.10 (1.05 to 1.16)$^{\beta}$	1.07 (1.03 to 1.10)	<0.001
5-9 years	4718; 21883	1.05 (1.01 to 1.09)	3210; 16348	1.03 (0.98 to 1.07)	1.04 (1.01 to 1.07)	0.006
10+ years	4202; 18466	1.07 (1.03 to 1.12)$^{\beta}$	2694; 14763	0.93 (0.89 to 0.98)$^{\alpha}$	1.01 (0.98 to 1.05)	0.4
OESTROGEN ONLY						
13-23 months	2072; 9332	1.16 (1.09 to 1.22)$^{\beta}$	1458; 6342	1.26 (1.18 to 1.35)$^{\beta}$	1.19 (1.15 to 1.25)	<0.001
2-4 years	804; 4370	0.92 (0.85 to 1.00)	643; 3185	1.07 (0.97 to 1.17)	0.98 (0.92 to 1.04)	0.5
5-9 years	1235; 5964	1.02 (0.96 to 1.09)	897; 4937	0.94 (0.87 to 1.02)	0.99 (0.94 to 1.04)	0.6
10+ years	1015; 4636	1.05 (0.97 to 1.13)	736; 4033	0.93 (0.85 to 1.01)	1.00 (0.94 to 1.05)	0.9
**Conjugated equine oestrogen**						
13-23 months	833; 3910	1.09 (1.01 to 1.18)	561; 2462	1.22 (1.11 to 1.35)$^{\beta}$	1.14 (1.07 to 1.21)	<0.001
2-4 years	419; 2216	0.93 (0.83 to 1.03)	298; 1464	1.03 (0.90 to 1.17)	0.97 (0.89 to 1.05)	0.4
5-9 years	674; 3463	0.96 (0.88 to 1.04)	483; 2629	0.95 (0.86 to 1.05)	0.95 (0.89 to 1.02)	0.2
10+ years	754; 3662	0.98 (0.90 to 1.07)	586; 3067	1.00 (0.90 to 1.10)	0.99 (0.93 to 1.05)	0.7
**Estradiol**						
13-23 months	1277; 5641	1.16 (1.08 to 1.23)$^{\beta}$	932; 4016	1.23 (1.14 to 1.33)$^{\beta}$	1.18 (1.13 to 1.25)	<0.001
2-4 years	527; 2934	0.89 (0.81 to 0.98)	456; 2246	1.04 (0.94 to 1.16)	0.95 (0.89 to 1.02)	0.2
5-9 years	849; 4027	1.03 (0.95 to 1.12)	641; 3560	0.91 (0.83 to 1.00)	0.98 (0.92 to 1.04)	0.5
10+ years	720; 3374	1.02 (0.93 to 1.11)	547; 3129	0.87 (0.79 to 0.96)$^{\alpha}$	0.95 (0.89 to 1.02)	0.2
OESTROGEN COMBINED with						
**any progestogen**						
13-23 months	6234; 18453	1.69 (1.64 to 1.75)$^{\beta}$	3701; 10854	1.91 (1.83 to 2.00)$^{\beta}$	1.77 (1.73 to 1.82)	<0.001
2-4 years	2467; 11088	1.09 (1.04 to 1.14)$^{\beta}$	1503; 7323	1.10 (1.03 to 1.16)$^{\alpha}$	1.09 (1.05 to 1.13)	<0.001

5-9 years	3486; 15928	1.05 (1.01 to 1.09)	2313; 11411	1.05 (1.00 to 1.10)	1.05 (1.01 to 1.08)	0.004
10+ years	3187; 13845	1.07 (1.02 to 1.12)[α]	1958; 10730	0.92 (0.87 to 0.97)[α]	1.01 (0.97 to 1.05)	0.6
**Medroxyprogesterone**						
13-23 months	1499; 4049	1.60 (1.50 to 1.70)[β]	817; 2258	1.72 (1.58 to 1.87)[β]	1.64 (1.56 to 1.72)	<0.001
2-4 years	884; 3506	1.06 (0.98 to 1.15)	520; 1999	1.18 (1.07 to 1.31)[α]	1.11 (1.04 to 1.18)	0.001
5-9 years	1251; 5192	1.03 (0.97 to 1.10)	735; 3353	1.05 (0.96 to 1.14)	1.04 (0.98 to 1.09)	0.2
10+ years	1056; 4406	1.02 (0.95 to 1.10)	574; 2920	1.00 (0.90 to 1.10)	1.01 (0.96 to 1.07)	0.7
Levonorgestrel						
13-23 months	1626; 4954	1.44 (1.36 to 1.53)[β]	849; 2467	1.73 (1.59 to 1.88)[β]	1.53 (1.46 to 1.61)	<0.001
2-4 years	1420; 5064	1.16 (1.08 to 1.23)[β]	713; 2673	1.18 (1.08 to 1.28)[β]	1.16 (1.10 to 1.22)	<0.001
5-9 years	2371; 9151	1.12 (1.07 to 1.18)[β]	1425; 5864	1.13 (1.06 to 1.21)[β]	1.13 (1.08 to 1.17)	<0.001
10+ years	3057; 12235	1.11 (1.06 to 1.17)[β]	1979; 10045	0.96 (0.91 to 1.02)	1.05 (1.01 to 1.09)	0.009
**Norethisterone**						
13-23 months	2955; 8677	1.54 (1.47 to 1.61)[β]	1801; 5142	1.78 (1.67 to 1.88)[β]	1.63 (1.57 to 1.69)	<0.001
2-4 years	1615; 6825	1.02 (0.96 to 1.08)	998; 4620	1.03 (0.96 to 1.11)	1.03 (0.98 to 1.07)	0.3
5-9 years	2354; 10044	1.03 (0.98 to 1.08)	1555; 7190	1.05 (0.99 to 1.12)	1.04 (1.00 to 1.08)	0.05
10+ years	2227; 9345	1.05 (0.99 to 1.11)	1329; 6979	0.95 (0.89 to 1.02)	1.01 (0.97 to 1.05)	0.6
**Dydrogesterone**						
13-23 months	390; 1435	1.09 (0.97 to 1.23)	204; 786	1.13 (0.96 to 1.33)	1.10 (1.01 to 1.21)	0.04
2-4 years	328; 1414	0.88 (0.77 to 0.99)	179; 814	0.93 (0.79 to 1.10)	0.90 (0.81 to 0.99)	0.03
5-9 years	506; 2100	0.96 (0.87 to 1.06)	276; 1425	0.87 (0.76 to 1.00)	0.93 (0.85 to 1.00)	0.06
10+ years	505; 2053	1.00 (0.90 to 1.11)	226; 1219	0.89 (0.76 to 1.03)	0.96 (0.89 to 1.05)	0.4
TIBOLONE						
13-23 months	648; 2476	1.14 (1.04 to 1.25)[α]	463; 1585	1.37 (1.23 to 1.52)[β]	1.23 (1.15 to 1.32)	<0.001
2-4 years	468; 1994	1.00 (0.90 to 1.11)	316; 1544	0.93 (0.82 to 1.05)	0.97 (0.89 to 1.05)	0.4
5-9 years	756; 3220	1.01 (0.93 to 1.10)	527; 2556	0.97 (0.88 to 1.07)	0.99 (0.93 to 1.06)	0.9
10+ years	643; 2872	0.96 (0.88 to 1.05)	487; 2480	0.99 (0.89 to 1.10)	0.97 (0.91 to 1.04)	0.4

[#]Odds ratios are with reference to never-users and based on cases and controls matched by age and practice and adjusted for smoking status, body mass index, family history of cancer, medical conditions and events, other medications and contraceptive drugs.; [α] P-value<0.01; [β] P-value<0.001

*eTable 6 **All cases and controls:** Duration of use for different hormones of HRT and tibolone in women with **recent** (**between >1 and <5 years** before the index date) and **past** (**5 years or more** before the index date)exposures, combined analysis*

	Recent use (between >1 and <5 years)			Past use (5 years or more)		
	N of cases; controls	Mean duration: cases; controls	Combined odds ratio[#] (95% confidence interval)	N of cases; controls	Mean duration: cases; controls	Combined odds ratio[#] (95% confidence interval)
OESTROGEN ONLY						
<1 year	629; 2917	0.5; 0.5	1.09 (1.00 to 1.19)	1241; 5928	0.4; 0.4	1.07 (1.00 to 1.14)
1-2 years	962; 4626	2.0; 2.0	1.08 (1.00 to 1.16)	884; 4637	1.9; 1.9	0.98 (0.91 to 1.06)
3-4 years	940; 4265	4.0; 4.0	1.15 (1.07 to 1.24)[β]	601; 3033	4.0; 4.0	1.01 (0.93 to 1.11)
5-9 years	1665; 7829	7.3; 7.2	1.14 (1.08 to 1.21)[β]	929; 4813	7.1; 7.2	1.01 (0.94 to 1.08)
10+ years	781; 3592	13.0; 13.1	1.17 (1.08 to 1.27)[β]	228; 1159	11.9; 11.8	0.90 (0.70 to 1.15)
For each year of use			1.02 (1.01 to 1.02)[β]			1.00 (0.99 to 1.01)
**Conjugated equine oestrogen**						
<1 year	284; 1431	0.5; 0.5	0.96 (0.84 to 1.09)	1018; 5294	0.4; 0.4	0.97 (0.91 to 1.04)
1-2 years	373; 1919	2.0; 2.0	0.98 (0.87 to 1.10)	593; 2938	1.8; 1.8	1.01 (0.92 to 1.11)
3-4 years	426; 1830	4.0; 4.0	1.19 (1.07 to 1.33)[α]	324; 1632	3.9; 3.9	0.99 (0.88 to 1.12)
5-9 years	736; 3397	7.2; 7.2	1.15 (1.06 to 1.25)[α]	562; 2957	8.0; 8.0	0.97 (0.88 to 1.06)
10+ years	292; 1475	12.9; 12.9	1.04 (0.92 to 1.18)	n/d	n/d	n/d
For each year of use			1.01 (1.00 to 1.02)			0.99 (0.98 to 1.00)
**Estradiol**						
<1 year	601; 2823	0.4; 0.5	1.06 (0.96 to 1.16)	1083; 5589	0.4; 0.4	0.97 (0.90 to 1.04)
1-2 years	733; 3444	2.0; 2.0	1.07 (0.99 to 1.17)	699; 3569	1.9; 1.9	0.99 (0.91 to 1.08)
3-4 years	569; 2731	3.9; 4.0	1.07 (0.97 to 1.17)	383; 1977	3.9; 3.9	0.97 (0.87 to 1.09)
5-9 years	891; 4172	7.2; 7.1	1.12 (1.04 to 1.21)[α]	592; 2955	7.8; 7.8	1.01 (0.92 to 1.11)
10+ years	398; 1667	13.0; 13.1	1.25 (1.12 to 1.40)[β]	n/d	n/d	n/d
For each year of use			1.02 (1.01 to 1.02)[β]			1.00 (0.99 to 1.01)

OESTROGEN COMBINED with						
**any progestogen**						
<1 year	1911; 8627	0.5; 0.5	1.11 (1.06 to 1.17)[β]	3501; 18025	0.5; 0.4	0.98 (0.94 to 1.02)
1-2 years	2945; 11239	2.0; 2.0	1.34 (1.28 to 1.40)[β]	2834; 13466	1.9; 1.9	1.05 (1.01 to 1.10)
3-4 years	2671; 9166	4.0; 4.0	1.50 (1.43 to 1.57)[β]	1766; 8183	4.0; 3.9	1.08 (1.02 to 1.14)[α]
5-9 years	4537; 14035	7.2; 7.1	1.70 (1.64 to 1.76)[β]	2373; 10413	7.0; 7.0	1.17 (1.11 to 1.22)[β]
10+ years	1841; 4651	12.9; 12.7	2.05 (1.94 to 2.17)[β]	470; 1827	11.6; 11.8	1.09 (0.91 to 1.30)
For each year of use[γ]			1.22 (1.21 to 1.23)[β]			1.02 (1.01 to 1.03)[β]
**Medroxyprogesterone**						
<1 year	1181; 4293	0.4; 0.4	1.14 (1.07 to 1.22)[β]	1924; 8724	0.4; 0.4	0.98 (0.93 to 1.03)
1-2 years	1085; 3583	1.9; 1.9	1.32 (1.23 to 1.42)[β]	963; 4116	1.9; 1.8	1.03 (0.96 to 1.11)
3-4 years	714; 2047	3.9; 3.9	1.62 (1.48 to 1.77)[β]	455; 1836	3.9; 3.9	1.14 (1.03 to 1.27)
5-9 years	621; 1673	6.8; 6.6	1.78 (1.62 to 1.96)[β]	274; 1195	6.4; 6.4	1.07 (0.94 to 1.23)
10+ years	119; 216	12.0; 12.1	2.63 (2.08 to 3.31)[β]	n/d	n/d	n/d
For each year of use			1.10 (1.09 to 1.11)[β]			1.02 (1.00 to 1.03)
**Levonorgestrel**						
<1 year	1058; 4167	0.5; 0.5	1.12 (1.04 to 1.20)[α]	3716; 17049	0.4; 0.4	0.99 (0.95 to 1.03)
1-2 years	1152; 3943	2.0; 1.9	1.33 (1.24 to 1.43)[β]	2436; 10199	1.9; 1.8	1.05 (1.00 to 1.10)
3-4 years	884; 2889	4.0; 4.0	1.44 (1.33 to 1.56)[β]	1269; 4987	3.9; 3.9	1.12 (1.05 to 1.19)[β]
5-9 years	1217; 3455	7.0; 7.0	1.73 (1.62 to 1.86)[β]	1411; 5060	7.2; 7.2	1.24 (1.16 to 1.32)[β]
10+ years	297; 704	12.3; 12.3	2.13 (1.85 to 2.44)[β]	n/d	n/d	n/d
For each year of use[δ]			1.08 (1.07 to 1.08)[β]			1.07 (1.05 to 1.08)[β]
**Norethisterone**						
<1 year	1931; 8218	0.5; 0.4	1.02 (0.97 to 1.08)	3764; 17319	0.4; 0.4	1.01 (0.97 to 1.05)
1-2 years	1988; 7119	2.0; 1.9	1.28 (1.22 to 1.35)[β]	1954; 8700	1.8; 1.8	1.05 (0.99 to 1.10)
3-4 years	1362; 4401	3.9; 3.9	1.50 (1.41 to 1.60)[β]	913; 4010	3.9; 3.9	1.07 (0.99 to 1.15)
5-9 years	1680; 4633	7.0; 6.9	1.82 (1.72 to 1.93)[β]	834; 3529	7.0; 7.0	1.14 (1.05 to 1.23)[α]
10+ years	408; 893	12.5; 12.2	2.28 (2.02 to 2.57)[β]	n/d	n/d	n/d
For each year of use[γ]			1.24 (1.22 to 1.26)[β]			1.02 (1.01 to 1.02)[β]

**Dydrogesterone**						
<1 year	503; 1922	0.4; 0.4	1.05 (0.94 to 1.16)	953; 4229	0.4; 0.4	0.94 (0.87 to 1.01)
1-2 years	307; 1350	1.9; 1.8	0.96 (0.85 to 1.09)	390; 1704	1.8; 1.8	0.97 (0.86 to 1.08)
3-4 years	136; 601	4.0; 3.9	1.02 (0.85 to 1.24)	103; 568	3.7; 3.8	0.78 (0.63 to 0.96)
5-9 years	127; 499	6.8; 6.7	1.18 (0.97 to 1.44)	67; 296	6.7; 6.8	1.07 (0.81 to 1.40)
10+ years	28; 77	11.3; 11.7	1.72 (1.11 to 2.67)	n/d	n/d	n/d
For each year of use			1.02 (1.00 to 1.04)			0.98 (0.95 to 1.01)
TIBOLONE						
<1 year	656; 2850	0.4; 0.4	0.95 (0.87 to 1.04)	1527; 7035	0.4; 0.4	0.97 (0.92 to 1.03)
1-2 years	501; 1904	1.9; 1.9	1.13 (1.03 to 1.26)	512; 2353	1.8; 1.8	0.98 (0.89 to 1.08)
3-4 years	278; 1167	4.0; 3.9	1.13 (0.99 to 1.29)	201; 918	3.9; 3.9	1.02 (0.87 to 1.19)
5-9 years	371; 1376	7.1; 7.0	1.30 (1.16 to 1.46)[β]	173; 822	7.2; 7.3	1.02 (0.86 to 1.21)
10+ years	89; 302	11.9; 12.7	1.46 (1.15 to 1.86)[α]	n/d	n/d	n/d
For each year of use			1.03 (1.02 to 1.05)[β]			1.00 (0.98 to 1.02)

[#]Odds ratios are with reference to never-users and based on cases and controls matched by age and practice and adjusted for smoking status, body mass index, family history of cancer, medical conditions and events, other medications and contraceptive drugs; [α] P-value<0.01; [β] P-value<0.001; n/d – not enough data, few observations added to previous category;

[γ] duration for recent use was transformed as square root (exposure)

[δ] duration for past use was transformed as square root (exposure)

eTable 7 ***All cases and controls:*** *Duration of use for different doses of hormones and types of application in women with* ***recent*** *(**between >1 and <5** years before the index date) and* ***past*** *(**5 years or more** before the index date)exposures, combined analysis*

	Recent use (between >1 and <5 years)		Past use (5 years or more)	
	N of cases; controls	Adjusted odds ratio[#] (95% confidence interval)	N of cases; controls	Adjusted odds ratio[#] (95% confidence interval)
OESTROGEN ONLY				
**Conjugated equine oestrogen**				
≤0.625mg				
<1 year	221; 1111	0.98 (0.84 to 1.13)	770; 4012	0.98 (0.90 to 1.06)
1-4 years	553; 2618	1.09 (0.99 to 1.20)	671; 3186	1.06 (0.97 to 1.15)
5+ years	655; 3097	1.12 (1.03 to 1.23)[α]	370; 2008	0.94 (0.84 to 1.06)
>0.625mg				
<1 year	63; 320	0.95 (0.72 to 1.25)	248; 1282	1.00 (0.87 to 1.15)
1-4 years	246; 1131	1.11 (0.97 to 1.28)	246; 1384	0.90 (0.78 to 1.03)
5+ years	373; 1775	1.15 (1.02 to 1.29)	192; 949	1.04 (0.89 to 1.22)
**Estradiol**				
≤1mg				
<1 year	518; 2407	1.08 (0.98 to 1.19)	934; 4703	1.01 (0.93 to 1.08)
1-4 years	974; 4623	1.09 (1.02 to 1.18)	865; 4365	1.01 (0.94 to 1.09)
5+ years	906; 4116	1.17 (1.08 to 1.26)[β]	455; 2222	1.04 (0.94 to 1.16)
>1mg				
<1 year	83; 416	1.00 (0.79 to 1.27)	149; 886	0.84 (0.70 to 1.00)
1-4 years	328; 1551	1.07 (0.95 to 1.21)	217; 1180	0.93 (0.80 to 1.08)
5+ years	383; 1723	1.19 (1.06 to 1.34)[α]	137; 733	0.95 (0.79 to 1.15)
oral				
<1 year	335; 1616	1.03 (0.91 to 1.16)	705; 3436	1.03 (0.95 to 1.12)
1-4 years	533; 2651	1.01 (0.92 to 1.11)	502; 2403	1.05 (0.95 to 1.16)
5+ years	415; 2037	1.06 (0.95 to 1.19)	189; 1004	0.94 (0.81 to 1.11)

transdermal				
<1 year	426; 2022	1.03 (0.93 to 1.15)	927; 4934	0.93 (0.87 to 1.01)
1-4 years	821; 3860	1.09 (1.00 to 1.18)	778; 4059	0.97 (0.89 to 1.05)
5+ years	833; 3533	1.23 (1.14 to 1.33)[β]	387; 1894	1.02 (0.91 to 1.14)
**Levonorgestrel**				
oral				
<1 year	943; 3773	1.10 (1.02 to 1.18)	3679; 16954	0.99 (0.95 to 1.02)
1-4 years	1798; 6043	1.39 (1.31 to 1.47)[β]	3635; 14870	1.08 (1.03 to 1.12)[β]
5+ years	1430; 3909	1.81 (1.70 to 1.93)[β]	1397; 4976	1.24 (1.17 to 1.33)[β]
transdermal	215; 768	1.13 (0.96 to 1.32)	250; 890	1.16 (1.00 to 1.34)
intra-uterine device	314; 971	1.29 (1.13 to 1.47)[β]	58; 314	0.74 (0.55 to 0.98)
**Norethisterone**				
oral				
<1 year	1764; 7539	1.01 (0.95 to 1.06)	3486; 15994	1.00 (0.96 to 1.04)
1-4 years	2846; 9441	1.40 (1.34 to 1.46)[β]	2318; 10275	1.04 (0.99 to 1.09)
5+ years	1692; 4358	1.94 (1.83 to 2.06)[β]	645; 2621	1.18 (1.08 to 1.29)[β]
transdermal				
<1 year	523; 2163	1.01 (0.91 to 1.11)	1458; 6467	0.99 (0.93 to 1.05)
1-4 years	601; 2260	1.21 (1.10 to 1.33)[β]	811; 3451	1.03 (0.95 to 1.12)
5+ years	307; 878	1.70 (1.49 to 1.94)[β]	168; 727	1.06 (0.89 to 1.25)

[#]Odds ratios are with reference to never-users and based on cases and controls matched by age and practice and adjusted for smoking status, body mass index, family history of cancer, medical conditions and events, other medications and contraceptive drugs.

[α] P-value<0.01; [β] P-value<0.001

eFigure 2 Exposure to HRT by definition of recency

Years of use	Recent (between >1 and <5 years) and past (5 years or more) use: Number of cases	Mean duration of use	Odds ratio (95% CI)	Recent (between >1 and <2 years) and past (2 years or more) use: Number of cases	Mean duration of use	Odds ratio (95% CI)
**Recent use**						
**Oestrogen only**						
<1 year	629	0.5	1.09 (1.00 to 1.19)	298	0.5	1.05 (0.93 to 1.20)
1-4 years	1902	3.0	1.11 (1.06 to 1.18)	1342	3.0	1.17 (1.10 to 1.24)
5+ years	2446	9.1	1.15 (1.09 to 1.21)	1890	9.2	1.25 (1.18 to 1.32)
**Oestrogen and progestogen**						
<1 year	1911	0.5	1.12 (1.06 to 1.18)	956	0.5	1.20 (1.12 to 1.29)
1-4 years	5616	2.9	1.42 (1.37 to 1.47)	4041	3.0	1.65 (1.59 to 1.71)
5+ years	6378	8.8	1.79 (1.73 to 1.85)	4938	9.0	2.10 (2.02 to 2.17)
**Past use**						
**Oestrogen only**						
<1 year	1241	0.4	1.07 (1.00 to 1.14)	1572	0.4	1.08 (1.02 to 1.14)
1-4 years	1485	2.7	0.99 (0.93 to 1.05)	2045	2.7	0.99 (0.94 to 1.05)
5+ years	1157	8.1	1.00 (0.93 to 1.07)	1713	8.3	0.95 (0.90 to 1.01)
**Oestrogen and progestogen**						
<1 year	3501	0.5	0.98 (0.94 to 1.01)	4456	0.5	0.99 (0.95 to 1.02)
1-4 years	4600	2.7	1.06 (1.02 to 1.09)	6175	2.7	1.05 (1.02 to 1.08)
5+ years	2843	7.8	1.16 (1.11 to 1.21)	4283	8.0	1.15 (1.11 to 1.19)

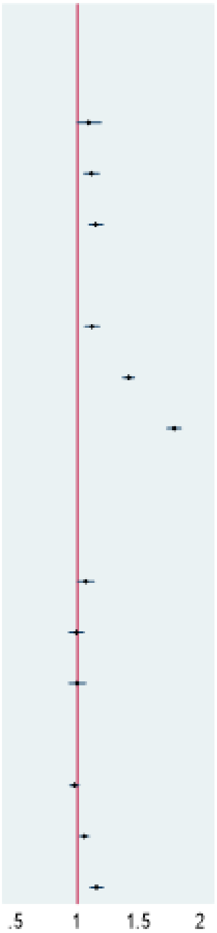

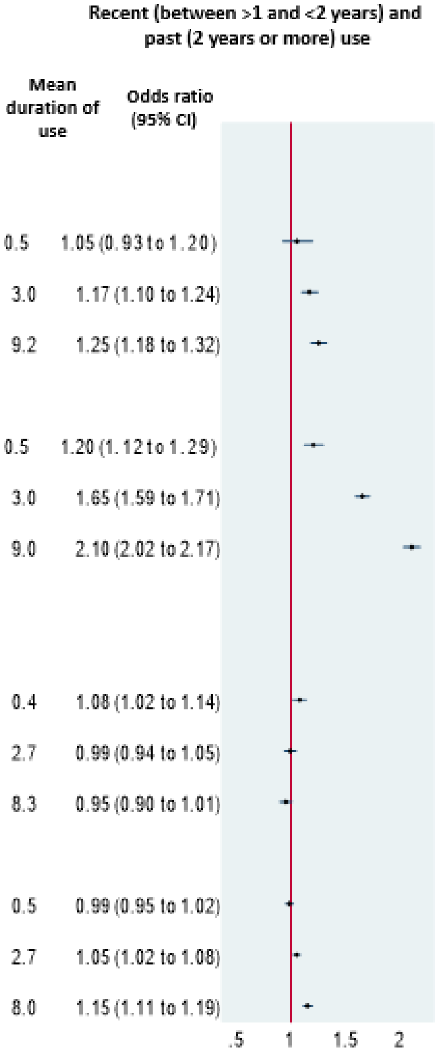

Odds ratios are with reference to never-users and adjusted for smoking, alcohol consumption, Townsend quintile (QResearch only), body mass index, ethnicity, history of other cancers, oophorectomy/hysterectomy, records of menopause, scans, comorbidities, other medications, years of data. Cases are matched to controls by age, general practice and index date.

eFigure 3 Duration of exposure to oestrogen-only and oestrogen-progestogen therapies and risks in recent (last use within >1 and <2 years before the index date) and past (≥2 years) users

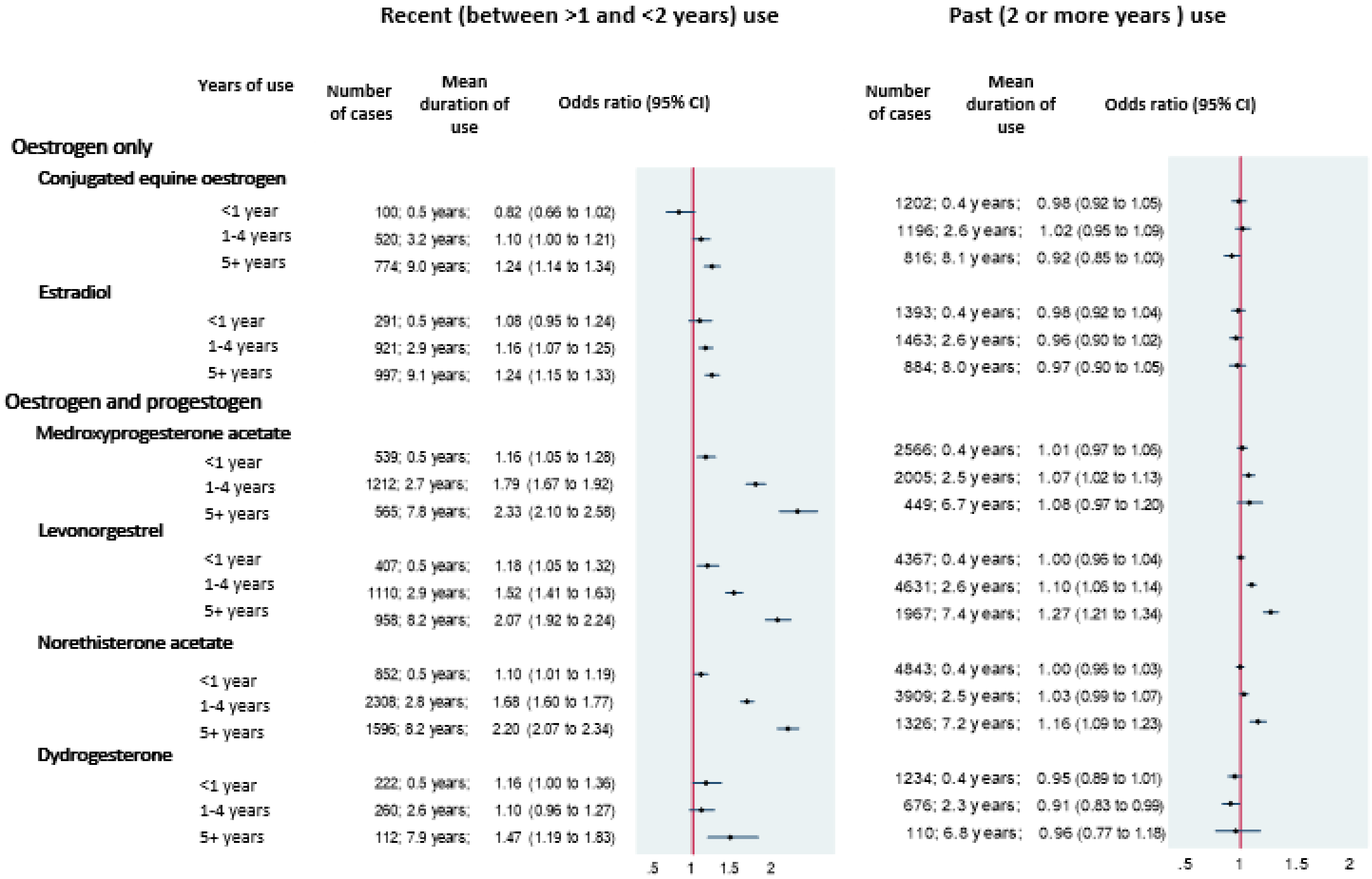

Odds ratios are with reference to never-users and adjusted for smoking, alcohol consumption, Townsend quintile (QResearch only), body mass index, ethnicity, history of other cancers, oophorectomy/hysterectomy, records of menopause, scans, comorbidities, other medications, years of data. Cases are matched to controls by age, general practice and index date.

eTable 8 ***All cases and controls:*** *Duration of use for different hormones of HRT and tibolone in women with* ***recent*** *(****between >1 and <2 years*** *before the index date) and* ***past*** *(****2 years or more*** *before the index date)exposures, combined analysis*

	**Recent exposure (between >1 and <2 years)**			**Past exposures (2 years or more)**		
	N of cases; controls	Mean duration: cases; controls	Combined odds ratio[#] (95% confidence interval)	N of cases; controls	Mean duration: cases; controls	Combined odds ratio[#] (95% confidence interval)
OESTROGEN ONLY						
<1 year	298; 1433	0.5; 0.5	1.05 (0.92 to 1.20)	1572; 7412	0.4; 0.4	1.08 (1.02 to 1.14)
1-2 years	648; 3037	2.0; 2.0	1.12 (1.02 to 1.22)	1198; 6226	1.9; 1.9	0.98 (0.92 to 1.05)
3-4 years	694; 3028	4.0; 4.0	1.22 (1.12 to 1.33)[β]	847; 4270	4.0; 4.0	1.01 (0.94 to 1.09)
5-9 years	1259; 5488	7.3; 7.2	1.25 (1.17 to 1.33)[β]	1335; 7154	7.2; 7.2	0.97 (0.91 to 1.03)
10+ years	631; 2688	13.1; 13.3	1.26 (1.15 to 1.38)[β]	378; 2063	12.2; 12.1	0.83 (0.69 to 1.01)
**Conjugated equine oestrogen**						
<1 year	100; 592	0.5; 0.5	0.82 (0.66 to 1.02)	1202; 6133	0.4; 0.4	0.98 (0.92 to 1.05)
1-2 years	221; 1149	2.0; 2.1	0.98 (0.85 to 1.14)	745; 3708	1.8; 1.9	1.01 (0.93 to 1.09)
3-4 years	299; 1287	4.0; 4.0	1.20 (1.06 to 1.37)[α]	451; 2175	3.9; 4.0	1.04 (0.94 to 1.15)
5-9 years	541; 2280	7.2; 7.2	1.28 (1.16 to 1.41)[β]	816; 4485	8.1; 8.2	0.92 (0.85 to 1.00)
10+ years	233; 1064	13.1; 13.1	1.16 (1.00 to 1.34)	n/d	n/d	n/d
**Estradiol**						
<1 year	291; 1326	0.5; 0.5	1.08 (0.95 to 1.24)	1393; 7086	0.4; 0.4	0.98 (0.92 to 1.04)
1-2 years	503; 2208	2.0; 2.0	1.16 (1.05 to 1.29)[α]	929; 4805	1.9; 1.9	0.97 (0.90 to 1.04)
3-4 years	418; 1892	3.9; 4.0	1.15 (1.03 to 1.28)	534; 2816	3.9; 3.9	0.95 (0.86 to 1.04)
5-9 years	675; 2954	7.2; 7.1	1.21 (1.11 to 1.32)[β]	884; 4563	8.0; 8.0	0.97 (0.90 to 1.05)
10+ years	322; 1277	13.1; 13.2	1.32 (1.17 to 1.50)[β]	n/d	n/d	n/d
OESTROGEN COMBINED with						
**any progestogen**						
<1 year	956; 4010	0.5; 0.5	1.20 (1.11 to 1.29)[β]	4456; 22642	0.5; 0.5	0.99 (0.95 to 1.02)
1-2 years	2052; 6821	2.0; 2.0	1.57 (1.49 to 1.65)[β]	3727; 17884	1.9; 1.9	1.04 (1.00 to 1.08)

3-4 years	1989; 6045	4.0; 4.0	1.73 (1.64 to 1.82)$^{\beta}$	2448; 11304	4.0; 3.9	1.08 (1.03 to 1.13)$^{\alpha}$
5-9 years	3415; 9114	7.2; 7.1	1.99 (1.91 to 2.08)$^{\beta}$	3495; 15334	7.1; 7.0	1.16 (1.12 to 1.20)$^{\beta}$
10+ years	1523; 3317	13.1; 12.9	2.37 (2.23 to 2.53)$^{\beta}$	788; 3161	11.8; 11.9	1.09 (0.95 to 1.25)
**Medroxyprogesterone**						
<1 year	539; 1887	0.5; 0.5	1.16 (1.05 to 1.28)$^{\alpha}$	2566; 11130	0.4; 0.4	1.01 (0.97 to 1.06)
1-2 years	706; 2026	1.9; 1.9	1.66 (1.52 to 1.81)$^{\beta}$	1342; 5673	1.9; 1.9	1.04 (0.98 to 1.11)
3-4 years	506; 1231	3.9; 3.9	2.02 (1.82 to 2.25)$^{\beta}$	663; 2652	3.9; 3.9	1.13 (1.04 to 1.24)$^{\alpha}$
5-9 years	465; 983	6.9; 6.7	2.27 (2.03 to 2.55)$^{\beta}$	449; 1921	6.7; 6.5	1.08 (0.97 to 1.20)
10+ years	100; 180	12.1; 12.2	2.65 (2.06 to 3.41)$^{\beta}$	n/d	n/d	n/d
**Levonorgestrel**						
<1 year	407; 1491	0.5; 0.5	1.18 (1.06 to 1.33)$^{\alpha}$	4367; 19725	0.4; 0.4	1.00 (0.97 to 1.04)
1-2 years	606; 1986	2.0; 2.0	1.45 (1.32 to 1.60)$^{\beta}$	2982; 12156	1.9; 1.8	1.08 (1.03 to 1.13)$^{\beta}$
3-4 years	504; 1561	4.0; 4.0	1.60 (1.45 to 1.78)$^{\beta}$	1649; 6315	3.9; 3.9	1.14 (1.08 to 1.21)$^{\beta}$
5-9 years	748; 1937	7.1; 7.0	1.99 (1.83 to 2.18)$^{\beta}$	1967; 6836	7.4; 7.3	1.28 (1.21 to 1.35)$^{\beta}$
10+ years	210; 446	12.4; 12.6	2.45 (2.07 to 2.90)$^{\beta}$	n/d	n/d	n/d
**Norethisterone**						
<1 year	852; 3392	0.5; 0.5	1.10 (1.01 to 1.19)	4843; 22145	0.4; 0.4	1.00 (0.96 to 1.03)
1-2 years	1327; 4076	2.0; 1.9	1.60 (1.50 to 1.71)$^{\beta}$	2615; 11743	1.8; 1.8	1.02 (0.97 to 1.07)
3-4 years	981; 2724	3.9; 3.9	1.82 (1.68 to 1.96)$^{\beta}$	1294; 5687	3.9; 3.9	1.06 (0.99 to 1.13)
5-9 years	1254; 2939	7.0; 6.9	2.16 (2.02 to 2.31)$^{\beta}$	1326; 5428	7.2; 7.1	1.16 (1.09 to 1.24)$^{\beta}$
10+ years	342; 688	12.7; 12.3	2.43 (2.13 to 2.77)$^{\beta}$	n/d	n/d	n/d
**Dydrogesterone**						
<1 year	222; 764	0.5; 0.4	1.17 (1.00 to 1.36)	1234; 5387	0.4; 0.4	0.95 (0.89 to 1.01)
1-2 years	177; 725	1.9; 1.9	1.15 (0.97 to 1.35)	520; 2329	1.8; 1.8	0.94 (0.85 to 1.03)
3-4 years	83; 372	4.0; 3.9	1.05 (0.83 to 1.34)	156; 797	3.8; 3.8	0.84 (0.70 to 1.00)
5+ years	112; 360	7.9; 7.6	1.48 (1.19 to 1.84)$^{\beta}$	110; 512	6.8; 6.9	0.96 (0.78 to 1.19)
TIBOLONE						
<1 year	261; 1062	0.4; 0.4	1.00 (0.87 to 1.15)	1922; 8823	0.4; 0.4	0.97 (0.92 to 1.02)

1-2 years	314; 1107	2.0; 1.9	1.37 (1.21 to 1.56)[β]	699; 3150	1.8; 1.8	0.99 (0.91 to 1.08)
3-4 years	196; 752	4.0; 3.9	1.28 (1.09 to 1.50)[α]	283; 1333	3.9; 3.9	0.98 (0.86 to 1.12)
5-9 years	270; 907	7.1; 7.0	1.44 (1.25 to 1.65)[β]	293; 1360	7.5; 7.4	1.04 (0.91 to 1.18)
10+ years	70; 233	12.0; 12.9	1.48 (1.13 to 1.94)[α]	n/d	n/d	n/d

[#]Odds ratios are with reference to never-users and based on cases and controls matched by age and practice and adjusted for smoking status, body mass index, family history of cancer, medical conditions and events, other medications and contraceptive drugs; [α] P-value<0.01; [β] P-value<0.001; n/d – not enough data, few observations added to previous category

eFigure 4 Recent (between >1 and <5 years) use of different types of HRT in women of different age

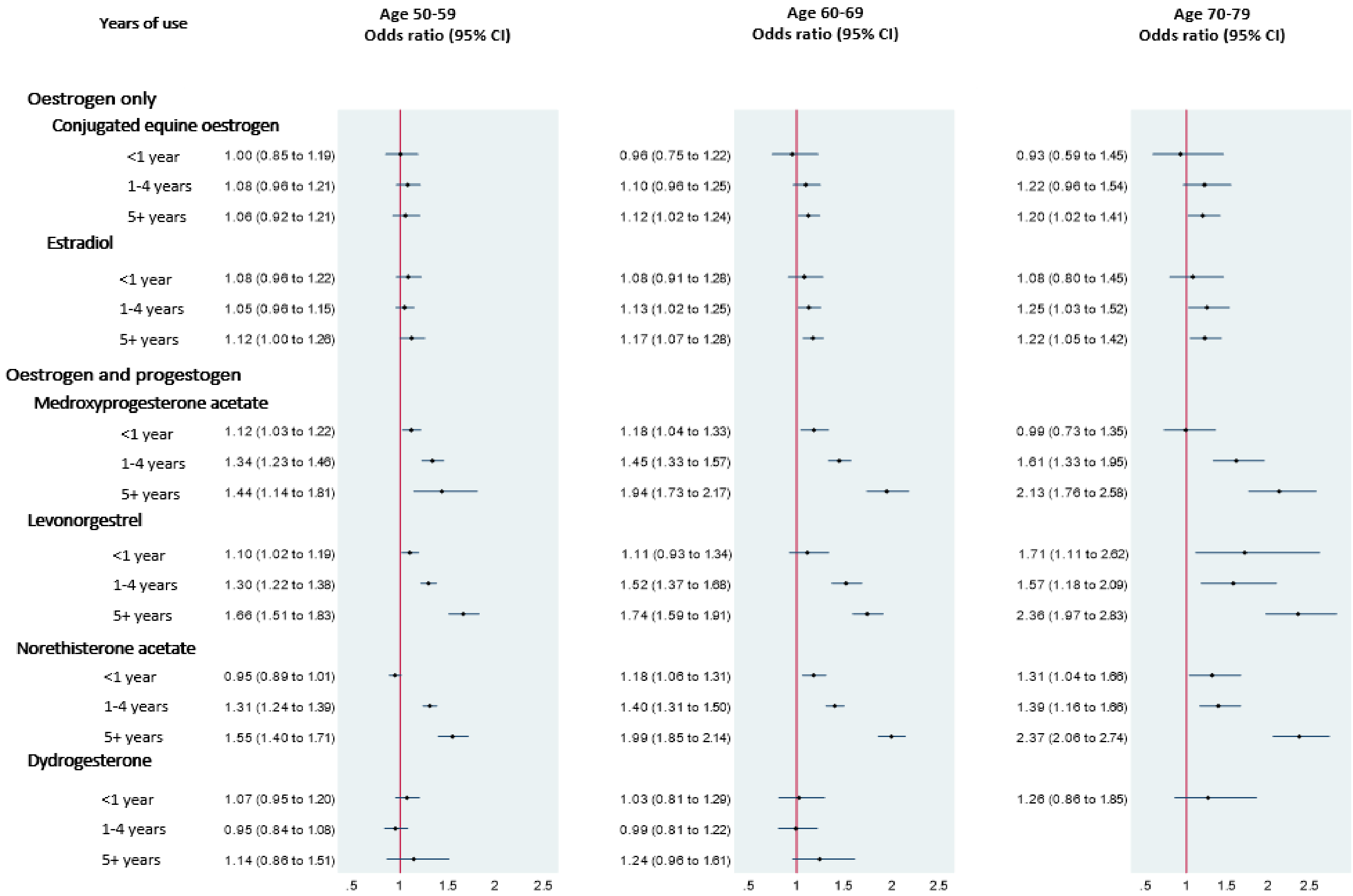

Odds ratios are with reference to never-users and adjusted for smoking, alcohol consumption, Townsend quintile (QResearch only), body mass index, ethnicity, history of other cancers, oophorectomy/hysterectomy, records of menopause, scans, comorbidities, other medications, years of data. Cases are matched to controls by age, general practice and index date.

*eTable 9 Duration of use for different hormones of HRT and tibolone in women with **recent** (between >1 and <5 years) different exposures by age at the index date*

	Age 50-59		Age 60-69		Age 70-79	
	N & mean duration for cases	Adjusted odds ratio# (95% confidence interval)	N & mean duration for cases	Adjusted odds ratio# (95% confidence interval)	N & mean duration for cases	Adjusted odds ratio# (95% confidence interval)
Total number of cases	35394		37015		26202	
OESTROGEN ONLY						
<1 year	384; 0.5	1.14 (1.02 to 1.28)	182; 0.5	1.09 (0.92 to 1.28)	63; 0.4	1.12 (0.84 to 1.48)
1-4 years	1010; 2.9	1.12 (1.03 to 1.21)α	691; 3.1	1.13 (1.04 to 1.24)α	201; 3.1	1.26 (1.07 to 1.48)α
5+ years	735; 7.9	1.08 (0.99 to 1.18)	1266; 9.3	1.17 (1.09 to 1.25)β	445; 10.7	1.25 (1.11 to 1.39)β
**Conjugated equine oestrogen**						
<1 year	181; 0.5	1.00 (0.85 to 1.19)	78; 0.5	0.96 (0.75 to 1.22)	25; 0.4	0.93 (0.59 to 1.45)
1-4 years	399; 2.9	1.08 (0.96 to 1.21)	308; 3.1	1.10 (0.96 to 1.25)	92; 3.2	1.22 (0.96 to 1.54)
5+ years	290; 7.8	1.06 (0.92 to 1.21)	538; 8.9	1.12 (1.02 to 1.24)	200; 10.3	1.20 (1.02 to 1.41)
**Estradiol**						
<1 year	366; 0.5	1.08 (0.96 to 1.22)	178; 0.4	1.08 (0.91 to 1.28)	57; 0.4	1.08 (0.80 to 1.45)
1-4 years	686; 2.7	1.05 (0.96 to 1.15)	479; 2.9	1.13 (1.02 to 1.25)	137; 3.0	1.25 (1.03 to 1.52)
5+ years	401; 7.7	1.12 (1.00 to 1.26)	663; 9.2	1.17 (1.07 to 1.28)β	225; 10.5	1.22 (1.05 to 1.42)α
OESTROGEN COMBINED with						
**any progestogen**						
<1 year	1461; 0.5	1.05 (0.99 to 1.12)	373; 0.5	1.27 (1.13 to 1.42)β	77; 0.4	1.34 (1.03 to 1.73)
1-4 years	3597; 2.8	1.34 (1.28 to 1.39)β	1742; 3.2	1.49 (1.41 to 1.58)β	277; 3.1	1.62 (1.41 to 1.87)β
5+ years	1982; 7.5	1.57 (1.48 to 1.66)β	3501; 9.1	1.83 (1.75 to 1.91)β	895; 11.1	2.20 (2.02 to 2.39)β
**Medroxyprogesterone**						
<1 year	745; 0.4	1.12 (1.03 to 1.22)	377; 0.5	1.18 (1.04 to 1.33)α	59; 0.4	0.99 (0.73 to 1.35)
1-4 years	769; 2.4	1.34 (1.23 to 1.46)β	868; 2.9	1.45 (1.33 to 1.57)β	162; 3.0	1.61 (1.33 to 1.95)β
5+ years	100; 6.9	1.44 (1.14 to 1.81)α	469; 7.4	1.94 (1.73 to 2.17)β	171; 8.8	2.13 (1.76 to 2.58)β
**Levonorgestrel**						
<1 year	870; 0.5	1.10 (1.02 to 1.19)	156; 0.5	1.11 (0.93 to 1.34)	32; 0.5	1.71 (1.11 to 2.62)

1-4 years	1421; 2.7	1.30 (1.22 to 1.38)[β]	546; 3.2	1.52 (1.37 to 1.68)[β]	69; 2.8	1.57 (1.18 to 2.09)[α]
5+ years	617; 7.1	1.66 (1.51 to 1.83)[β]	702; 8.3	1.74 (1.59 to 1.91)[β]	195; 10.2	2.36 (1.97 to 2.83)[β]
**Norethisterone**						
<1 year	1301; 0.5	0.95 (0.89 to 1.01)	523; 0.5	1.18 (1.06 to 1.31)[α]	107; 0.4	1.31 (1.04 to 1.66)
1-4 years	1939; 2.6	1.31 (1.24 to 1.39)[β]	1236; 3.0	1.40 (1.31 to 1.50)[β]	175; 2.9	1.39 (1.16 to 1.66)[β]
5+ years	559; 7.0	1.55 (1.40 to 1.71)[β]	1225; 8.2	1.99 (1.85 to 2.14)[β]	304; 9.6	2.37 (2.06 to 2.74)[β]
**Dydrogesterone**						
<1 year	395; 0.4	1.07 (0.95 to 1.20)	100; 0.4	1.03 (0.81 to 1.29)	38; 4.8	1.26 (0.86 to 1.85)
1-4 years	308; 2.5	0.95 (0.84 to 1.08)	120; 2.6	0.99 (0.81 to 1.22)	n/d	n/d
5+ years	64; 6.7	1.14 (0.86 to 1.51)	76; 8.1	1.24 (0.96 to 1.61)	n/d	n/d
TIBOLONE						
<1 year	327; 0.4	0.87 (0.77 to 0.99)	286; 0.4	1.07 (0.94 to 1.23)	43; 0.4	0.85 (0.61 to 1.20)
1-4 years	264; 2.3	1.08 (0.94 to 1.23)	409; 2.8	1.14 (1.02 to 1.27)	106; 3.0	1.25 (1.00 to 1.57)
5+ years	48; 7.0	1.21 (0.88 to 1.66)	258; 7.7	1.29 (1.12 to 1.48)[β]	154; 9.0	1.50 (1.25 to 1.81)[β]

[#]Odds ratios are with reference to never-users and based on cases and controls matched by age and practice and adjusted for smoking status, body mass index, family history of cancer, medical conditions and events, other medications and contraceptive drugs.

[α] P-value<0.01; [β] P-value<0.001

eTable 10 Duration of use for different hormones of HRT and tibolone in women with ***past*** *(5 years or more before the index date) different exposures by age at the index date*

	Age 50-59		Age 60-69		Age 70-79	
	N & mean duration for cases	Adjusted odds ratio# (95% confidence interval)	N & mean duration for cases	Adjusted odds ratio# (95% confidence interval)	N & mean duration for cases	Adjusted odds ratio# (95% confidence interval)
Total number of cases	35394		37015		26202	
OESTROGEN ONLY						
<1 year	250; 0.4	1.05 (0.91 to 1.21)	601; 0.4	1.05 (0.96 to 1.16)	390; 0.4	1.09 (0.97 to 1.22)
1-4 years	174; 2.5	0.94 (0.79 to 1.11)	817; 2.8	0.94 (0.87 to 1.02)	494; 2.7	1.08 (0.97 to 1.20)
5+ years	56; 7.1	1.03 (0.77 to 1.38)	590; 7.9	0.93 (0.84 to 1.02)	511; 8.4	1.06 (0.95 to 1.17)
**Conjugated equine oestrogen**						
<1 year	213; 0.4	0.86 (0.74 to 1.00)	534; 0.4	1.02 (0.93 to 1.13)	271; 0.4	0.97 (0.84 to 1.11)
1-4 years	135; 2.3	0.98 (0.81 to 1.19)	462; 2.7	0.91 (0.82 to 1.02)	320; 2.5	1.14 (1.00 to 1.29)
5+ years	27; 7.0	1.01 (0.67 to 1.53)	260; 7.7	0.86 (0.75 to 0.98)	275; 8.3	1.04 (0.91 to 1.19)
**Estradiol**						
<1 year	238; 0.4	0.92 (0.80 to 1.07)	537; 0.4	0.95 (0.86 to 1.05)	308; 0.4	1.03 (0.90 to 1.17)
1-4 years	149; 2.3	0.94 (0.78 to 1.12)	607; 2.6	0.93 (0.85 to 1.03)	326; 2.7	1.09 (0.96 to 1.24)
5+ years	29; 6.7	0.90 (0.60 to 1.34)	334; 7.6	1.00 (0.88 to 1.13)	229; 8.2	0.99 (0.85 to 1.15)
OESTROGEN COMBINED with						
**any progestogen**						
<1 year	850; 0.4	1.00 (0.92 to 1.08)	1880; 0.5	0.96 (0.91 to 1.01)	771; 0.5	1.02 (0.94 to 1.11)
1-4 years	606; 2.3	0.96 (0.87 to 1.05)	2805; 2.7	1.06 (1.01 to 1.11)	1189; 2.8	1.15 (1.07 to 1.23)$^{\beta}$
5+ years	107; 7.0	0.93 (0.75 to 1.14)	1619; 7.5	1.15 (1.08 to 1.22)$^{\beta}$	1117; 8.3	1.26 (1.17 to 1.35)$^{\beta}$
**Medroxyprogesterone**						
<1 year	354; 0.4	0.90 (0.80 to 1.02)	1126; 0.4	1.01 (0.94 to 1.08)	444; 0.4	0.98 (0.87 to 1.09)
1-4 years	104; 2.2	0.83 (0.67 to 1.03)	850; 2.5	1.07 (0.99 to 1.16)	470; 2.7	1.15 (1.03 to 1.29)
5+ years	n/d	n/d	145; 6.2	1.07 (0.89 to 1.29)	121; 6.7	1.10 (0.90 to 1.35)
**Levonorgestrel**						
<1 year	985; 0.4	1.04 (0.96 to 1.12)	1995; 0.4	0.97 (0.91 to 1.02)	736; 0.4	1.01 (0.93 to 1.11)

1-4 years	569; 2.2	1.03 (0.94 to 1.14)	2217; 2.6	1.07 (1.01 to 1.13)	919; 2.6	1.10 (1.02 to 1.20)
5+ years	89; 6.7	1.29 (1.01 to 1.64)	811; 7.0	1.18 (1.09 to 1.29)[β]	511; 7.6	1.32 (1.18 to 1.47)[β]
**Norethisterone**						
<1 year	867; 0.4	0.99 (0.91 to 1.07)	2105; 0.4	1.01 (0.96 to 1.06)	792; 0.4	1.07 (0.98 to 1.16)
1-4 years	394; 2.2	1.00 (0.89 to 1.12)	1760; 2.5	1.05 (1.00 to 1.12)	713; 2.5	1.11 (1.01 to 1.21)
5+ years	34; 6.3	0.89 (0.61 to 1.29)	473; 6.8	1.16 (1.04 to 1.29)[α]	327; 7.5	1.19 (1.04 to 1.35)[α]
**Dydrogesterone**						
<1 year	281; 0.4	0.87 (0.76 to 1.00)	574; 0.4	1.04 (0.94 to 1.15)	98; 0.4	0.74 (0.59 to 0.92)[α]
1-4 years	117; 2.2	0.92 (0.75 to 1.13)	306; 2.3	0.91 (0.80 to 1.03)	89; 2.9	1.06 (0.83 to 1.36)
5+ years	n/d	n/d	48; 6.7	1.04 (0.75 to 1.43)	n/d	n/d
TIBOLONE						
<1 year	219; 0.3	0.95 (0.82 to 1.10)	831; 0.4	0.98 (0.90 to 1.06)	477; 0.4	0.96 (0.87 to 1.07)
1-4 years	49; 2.3	1.04 (0.76 to 1.43)	393; 2.3	0.98 (0.88 to 1.10)	275; 2.6	0.99 (0.86 to 1.13)
5+ years	n/d	n/d	70; 7.0	1.03 (0.80 to 1.34)	99; 7.4	1.00 (0.80 to 1.25)

[#]Odds ratios are with reference to never-users and based on cases and controls matched by age and practice and adjusted for smoking status, body mass index, family history of cancer, medical conditions and events, other medications and contraceptive drugs.

[α] P-value<0.01; [β] P-value<0.001

eFigure 5 Recent (between >1 and <5 years) exposure to different types of HRT in women of different body mass index

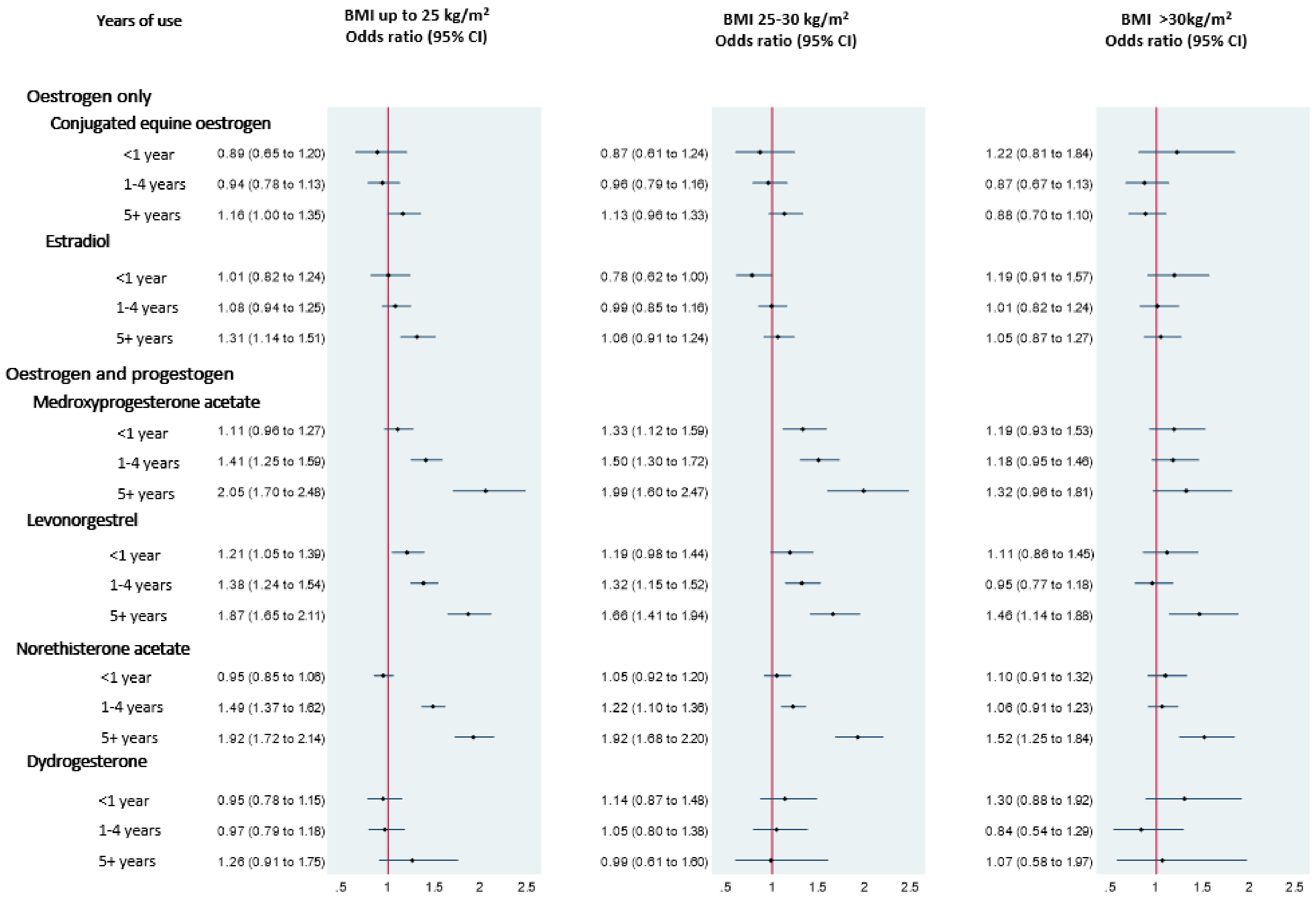

Odds ratios are with reference to never-users and adjusted for smoking, alcohol consumption, Townsend quintile (QResearch only), body mass index, ethnicity, history of other cancers, oophorectomy/hysterectomy, records of menopause, scans, comorbidities, other medications, years of data. Cases are matched to controls by age, general practice and index date.

eTable 11 Duration of use for different hormones of HRT and tibolone in women with ***recent*** *(between >1 and <5 years before the index date) different exposures by body-mass categories at 1years before the index date*

	BMI <25kg/m²		BMI 25-30kg/m²		BMI >30kg/m²	
	N of cases; controls	Adjusted odds ratio[#] (95% confidence interval)	N of cases; controls	Adjusted odds ratio[#] (95% confidence interval)	N of cases; controls	Adjusted odds ratio[#] (95% confidence interval)
Total number of women	25779		23110		17254	
OESTROGEN ONLY						
<1 year	156; 0.5	1.01 (0.82 to 1.24)	113; 0.5	0.81 (0.63 to 1.03)	109; 0.4	1.21 (0.92 to 1.58)
1-4 years	506; 2.9	1.09 (0.96 to 1.23)	441; 3.0	0.97 (0.85 to 1.11)	266; 3.0	1.02 (0.86 to 1.22)
5+ years	713; 9.1	1.24 (1.11 to 1.38)[β]	645; 9.3	1.10 (0.99 to 1.24)	357; 9.3	0.98 (0.85 to 1.14)
**Conjugated equine oestrogen**						
<1 year	71; 0.5	0.89 (0.65 to 1.20)	50; 0.5	0.87 (0.61 to 1.24)	48; 0.4	1.22 (0.81 to 1.84)
1-4 years	204; 3.1	0.94 (0.78 to 1.13)	189; 3.0	0.96 (0.79 to 1.16)	103; 3.0	0.87 (0.67 to 1.13)
5+ years	309; 9.0	1.16 (1.00 to 1.35)	285; 8.7	1.13 (0.96 to 1.33)	137; 9.0	0.88 (0.70 to 1.10)
**Estradiol**						
<1 year	158; 0.5	1.01 (0.82 to 1.24)	114; 0.4	0.78 (0.62 to 1.00)	107; 0.4	1.19 (0.91 to 1.57)
1-4 years	345; 2.7	1.08 (0.94 to 1.25)	318; 2.9	0.99 (0.85 to 1.16)	184; 2.9	1.01 (0.82 to 1.24)
5+ years	374; 8.8	1.31 (1.14 to 1.51)[β]	317; 9.4	1.06 (0.91 to 1.24)	205; 9.2	1.05 (0.87 to 1.27)
OESTROGEN COMBINED with						
**any progestogen**						
<1 year	605; 0.5	1.08 (0.97 to 1.20)	412; 0.5	1.14 (1.00 to 1.31)	239; 0.5	1.12 (0.93 to 1.34)
1-4 years	1987; 3.0	1.49 (1.39 to 1.59)[β]	1228; 3.0	1.36 (1.25 to 1.48)[β]	589; 2.9	1.13 (1.00 to 1.27)
5+ years	2316; 9.0	1.93 (1.80 to 2.06)[β]	1566; 8.8	1.71 (1.58 to 1.85)[β]	691; 8.9	1.38 (1.23 to 1.55)[β]
**Medroxyprogesterone**						
<1 year	399; 0.4	1.11 (0.96 to 1.27)	271; 0.5	1.33 (1.12 to 1.59)[α]	131; 0.5	1.19 (0.93 to 1.53)
1-4 years	582; 2.7	1.41 (1.25 to 1.59)[β]	454; 2.7	1.50 (1.30 to 1.72)[β]	182; 2.8	1.18 (0.95 to 1.46)
5+ years	264; 7.8	2.05 (1.70 to 2.48)[β]	198; 7.6	1.99 (1.60 to 2.47)[β]	83; 7.8	1.32 (0.96 to 1.81)

**Levonorgestrel**						
<1 year	388; 0.5	1.21 (1.05 to 1.39)[α]	217; 0.5	1.19 (0.98 to 1.44)	115; 0.5	1.11 (0.86 to 1.45)
1-4 years	742; 2.9	1.38 (1.24 to 1.54)[β]	437; 2.8	1.32 (1.15 to 1.52)[β]	174; 3.0	0.95 (0.77 to 1.18)
5+ years	561; 8.3	1.87 (1.65 to 2.11)[β]	344; 8.1	1.66 (1.41 to 1.94)[β]	138; 7.8	1.46 (1.14 to 1.88)[α]
**Norethisterone**						
<1 year	634; 0.5	0.95 (0.85 to 1.06)	446; 0.5	1.05 (0.92 to 1.20)	236; 0.4	1.10 (0.91 to 1.32)
1-4 years	1235; 2.7	1.49 (1.37 to 1.62)[β]	727; 2.8	1.22 (1.10 to 1.36)[β]	352; 2.8	1.06 (0.91 to 1.23)
5+ years	766; 8.1	1.92 (1.72 to 2.14)[β]	522; 8.0	1.92 (1.68 to 2.20)[β]	232; 8.3	1.52 (1.25 to 1.84)[β]
**Dydrogesterone**						
<1 year	188; 0.4	0.95 (0.78 to 1.15)	115; 0.5	1.14 (0.87 to 1.48)	60; 0.5	1.30 (0.88 to 1.92)
1-4 years	174; 2.6	0.97 (0.79 to 1.18)	97; 2.5	1.05 (0.80 to 1.38)	38; 2.5	0.84 (0.54 to 1.29)
5+ years	66; 7.7	1.26 (0.91 to 1.75)	28; 7.0	0.99 (0.61 to 1.60)	18; 7.9	1.07 (0.58 to 1.97)
TIBOLONE						
<1 year	210; 0.4	0.94 (0.79 to 1.13)	134; 0.4	0.84 (0.67 to 1.05)	76; 0.4	0.74 (0.55 to 1.00)
1-4 years	241; 2.7	1.10 (0.93 to 1.31)	186; 2.7	1.12 (0.92 to 1.37)	102; 2.7	1.12 (0.85 to 1.47)
5+ years	146; 7.8	1.45 (1.15 to 1.82)[α]	131; 8.1	1.45 (1.14 to 1.84)[α]	53; 8.0	1.02 (0.71 to 1.46)

[#]Odds ratios are with reference to never-users and based on cases and controls matched by age and practice and adjusted for smoking status, body mass index, family history of cancer, medical conditions and events, other medications and contraceptive drugs.

[α] P-value<0.01; [β] P-value<0.001

eTable 12 Duration of use for different hormones of HRT and tibolone in women with ***past*** *(5 years or more before the index date) different exposures by body-mass categories at 1 year before the index date*

	**BMI <25kg/m²**		**BMI 25-30kg/m²**		**BMI >30kg/m²**	
	N of cases; controls	Adjusted odds ratio[#] (95% confidence interval)	N of cases; controls	Adjusted odds ratio[#] (95% confidence interval)	N of cases; controls	Adjusted odds ratio[#] (95% confidence interval)
Total number of women	25779		23110		17254	
OESTROGEN ONLY						
<1 year	248; 0.5	1.03 (0.87 to 1.21)	314; 0.4	1.00 (0.86 to 1.16)	300; 0.4	1.04 (0.89 to 1.22)
1-4 years	307; 2.8	1.05 (0.90 to 1.22)	398; 2.8	0.96 (0.84 to 1.10)	398; 2.6	1.02 (0.89 to 1.18)
5+ years	260; 8.2	0.98 (0.83 to 1.15)	332; 8.1	1.08 (0.93 to 1.25)	299; 8.0	0.99 (0.84 to 1.16)
**Conjugated equine oestrogen**						
<1 year	194; 0.4	0.83 (0.69 to 1.00)	261; 0.4	0.92 (0.78 to 1.08)	239; 0.4	0.92 (0.78 to 1.10)
1-4 years	201; 2.6	1.07 (0.88 to 1.28)	249; 2.6	0.98 (0.83 to 1.16)	228; 2.5	1.15 (0.96 to 1.38)
5+ years	130; 8.1	1.02 (0.81 to 1.27)	167; 7.9	1.09 (0.89 to 1.34)	137; 7.9	0.99 (0.79 to 1.24)
**Estradiol**						
<1 year	226; 0.4	0.90 (0.76 to 1.07)	263; 0.4	0.89 (0.76 to 1.05)	287; 0.4	1.03 (0.88 to 1.21)
1-4 years	221; 2.5	1.00 (0.84 to 1.19)	291; 2.7	0.95 (0.81 to 1.11)	293; 2.6	1.01 (0.86 to 1.19)
5+ years	131; 8.0	0.92 (0.74 to 1.15)	176; 7.7	1.12 (0.92 to 1.36)	139; 7.7	0.86 (0.69 to 1.07)
OESTROGEN COMBINED with						
**any progestogen**						
<1 year	870; 0.5	0.96 (0.88 to 1.04)	931; 0.5	0.93 (0.85 to 1.02)	764; 0.4	1.00 (0.90 to 1.10)
1-4 years	1137; 2.7	1.04 (0.95 to 1.12)	1247; 2.7	1.01 (0.93 to 1.09)	999; 2.6	1.08 (0.99 to 1.19)
5+ years	781; 7.9	1.14 (1.03 to 1.26)	796; 7.8	1.17 (1.06 to 1.30)[α]	557; 7.7	1.19 (1.05 to 1.34)[α]
**Medroxyprogesterone**						
<1 year	540; 0.4	0.99 (0.88 to 1.12)	510; 0.4	0.94 (0.84 to 1.06)	400; 0.4	1.08 (0.94 to 1.25)
1-4 years	387; 2.6	1.11 (0.97 to 1.27)	367; 2.5	1.01 (0.88 to 1.17)	273; 2.5	0.99 (0.84 to 1.17)
5+ years	85; 6.3	1.17 (0.87 to 1.56)	79; 6.5	1.22 (0.90 to 1.65)	37; 6.5	0.65 (0.43 to 0.97)
**Levonorgestrel**						

<1 year	988; 0.5	0.96 (0.88 to 1.05)	948; 0.4	0.93 (0.85 to 1.02)	731; 0.4	1.13 (1.01 to 1.25)
1-4 years	1025; 2.6	1.07 (0.97 to 1.17)	1022; 2.5	1.10 (1.00 to 1.21)	669; 2.5	1.11 (0.99 to 1.24)
5+ years	478; 7.4	1.31 (1.15 to 1.50)[β]	372; 7.1	1.19 (1.03 to 1.37)	231; 7.1	1.26 (1.05 to 1.52)
**Norethisterone**						
<1 year	1019; 0.4	0.96 (0.88 to 1.04)	1044; 0.4	1.02 (0.93 to 1.11)	752; 0.4	1.04 (0.93 to 1.15)
1-4 years	747; 2.5	0.97 (0.88 to 1.07)	815; 2.5	1.08 (0.97 to 1.19)	588; 2.5	1.08 (0.96 to 1.22)
5+ years	225; 7.1	1.03 (0.87 to 1.23)	232; 7.0	1.12 (0.94 to 1.34)	158; 7.0	1.32 (1.05 to 1.65)
**Dydrogesterone**						
<1 year	271; 0.4	0.97 (0.83 to 1.14)	235; 0.4	0.88 (0.74 to 1.05)	187; 0.4	0.97 (0.79 to 1.18)
1-4 years	148; 2.2	0.98 (0.79 to 1.22)	136; 2.2	0.98 (0.77 to 1.24)	86; 2.3	0.80 (0.61 to 1.07)
5+ years	21; 6.9	0.97 (0.55 to 1.74)	18; 6.5	1.13 (0.61 to 2.08)	14; 6.8	1.30 (0.61 to 2.76)
TIBOLONE						
<1 year	366; 0.4	0.93 (0.81 to 1.06)	434; 0.4	0.98 (0.86 to 1.11)	342; 0.4	1.16 (1.00 to 1.36)
1-4 years	151; 2.4	0.82 (0.67 to 1.01)	205; 2.4	0.92 (0.77 to 1.10)	168; 2.4	1.14 (0.92 to 1.41)
5+ years	50; 7.3	1.32 (0.91 to 1.93)	46; 7.4	0.91 (0.63 to 1.31)	31; 6.6	0.88 (0.56 to 1.39)

[#]Odds ratios are with reference to never-users and based on cases and controls matched by age and practice and adjusted for smoking status, body mass index, family history of cancer, medical conditions and events, other medications and contraceptive drugs.

[α] P-value<0.01; [β] P-value<0.001

eFigure 6 Sensitivity analyses: women with 10 years of records and women 55 years and older

Years of use	Recent (between >1 and <5 years) and past use in women with at least 10 years of records: Number of cases	Mean duration of use	Odds ratio (95% CI)	Recent (between >1 and <5 years) and past use in women aged 55 years and older: Number of cases	Mean duration of use	Odds ratio (95% CI)
**Recent use**						
**Oestrogen only**						
<1 year	296	0.4	0.97 (0.85 to 1.10)	415	0.5	1.12 (1.00 to 1.25)
1-4 years	835	2.9	1.07 (0.98 to 1.16)	1464	3.0	1.17 (1.10 to 1.24)
5+ years	1635	9.7	1.10 (1.04 to 1.17)	2223	9.3	1.17 (1.11 to 1.23)
**Oestrogen and progestogen**						
<1 year	1023	0.5	1.09 (1.02 to 1.17)	1059	0.5	1.23 (1.14 to 1.32)
1-4 years	2751	2.9	1.34 (1.28 to 1.40)	4102	3.1	1.52 (1.47 to 1.58)
5+ years	4415	9.4	1.77 (1.70 to 1.84)	5936	9.0	1.88 (1.82 to 1.94)
**Past use**						
**Oestrogen only**						
<1 year	953	0.4	1.09 (1.01 to 1.17)	1156	0.4	1.07 (1.00 to 1.15)
1-4 years	1200	2.8	0.99 (0.93 to 1.06)	1446	2.7	0.99 (0.94 to 1.06)
5+ years	1022	8.1	1.00 (0.93 to 1.07)	1153	8.1	1.00 (0.93 to 1.07)
**Oestrogen and progestogen**						
<1 year	2691	0.5	0.98 (0.94 to 1.03)	3244	0.5	0.98 (0.94 to 1.02)
1-4 years	3819	2.7	1.09 (1.04 to 1.13)	4489	2.7	1.07 (1.04 to 1.11)
5+ years	2543	7.8	1.17 (1.11 to 1.23)	2832	7.8	1.17 (1.12 to 1.23)

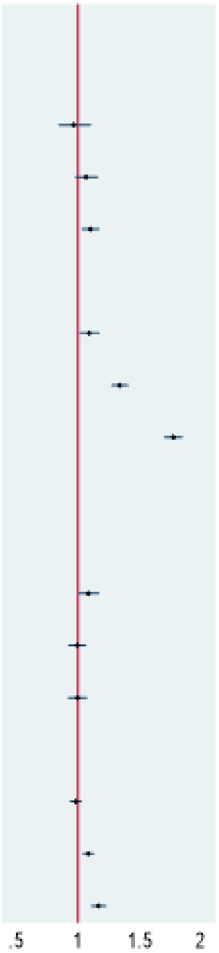

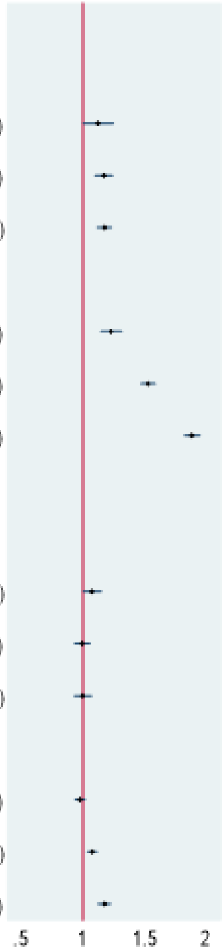

Odds ratios are with reference to never-users and adjusted for smoking, alcohol consumption, Townsend quintile (QResearch only), body mass index, ethnicity, history of other cancers, oophorectomy/hysterectomy, records of menopause, scans, comorbidities, other medications, years of data. Cases are matched to controls by age, general practice and index date.

eTable 13 ***All cases and controls with 10 years of records:*** *Duration of use for different hormones of HRT and tibolone in women with* ***recent*** *(****between >1 and <5 years*** *before the index date) and* ***past*** *(****5 years*** *or more before the index date) exposures, combined analysis*

	**Recent exposure (between >1 and <5 years)**			**Past exposures (5 years or more)**		
Total number of women: 62709 cases and 259147 controls	N of cases; controls	Mean duration: cases; controls	Combined odds ratio[#] (95% confidence interval)	N of cases; controls	Mean duration: cases; controls	Combined odds ratio[#] (95% confidence interval)
OESTROGEN ONLY						
<1 year	296; 1288	0.4; 0.5	0.96 (0.85 to 1.10)	953; 4020	0.4; 0.4	1.09 (1.01 to 1.17)
1-2 years	438; 1828	1.9; 2.0	1.08 (0.97 to 1.21)	696; 3262	1.9; 1.9	0.99 (0.90 to 1.08)
3-4 years	397; 1711	4.0; 4.0	1.05 (0.94 to 1.18)	504; 2240	4.0; 4.0	1.02 (0.92 to 1.13)
5-9 years	986; 4269	7.5; 7.5	1.08 (1.00 to 1.16)	814; 3787	7.2; 7.2	1.00 (0.92 to 1.09)
10+ years	649; 2661	13.1; 13.2	1.15 (1.05 to 1.26)[α]	208; 945	11.9; 11.8	1.02 (0.88 to 1.20)
**Conjugated equine oestrogen**						
<1 year	119; 559	0.4; 0.4	0.84 (0.68 to 1.03)	805; 3646	0.4; 0.4	1.00 (0.92 to 1.09)
1-2 years	164; 662	1.9; 2.0	1.04 (0.87 to 1.25)	454; 2030	1.8; 1.8	1.00 (0.90 to 1.11)
3-4 years	156; 672	4.0; 4.0	1.03 (0.86 to 1.23)	267; 1212	3.9; 4.0	0.98 (0.85 to 1.12)
5-9 years	436; 1824	7.4; 7.5	1.10 (0.98 to 1.23)	494; 2355	8.0; 8.1	0.97 (0.87 to 1.07)
10+ years	240; 1086	13.0; 13.0	1.03 (0.89 to 1.19)	n/d	n/d	n/d
**Estradiol**						
<1 year	310; 1332	0.4; 0.4	0.99 (0.87 to 1.13)	848; 3867	0.4; 0.4	0.99 (0.92 to 1.07)
1-2 years	369; 1528	1.9; 1.9	1.08 (0.96 to 1.22)	550; 2588	1.9; 1.9	0.96 (0.87 to 1.06)
3-4 years	274; 1254	4.0; 4.0	0.99 (0.87 to 1.13)	333; 1475	4.0; 3.9	1.00 (0.89 to 1.14)
5-9 years	542; 2338	7.4; 7.3	1.06 (0.96 to 1.17)	523; 2323	7.8; 7.8	1.01 (0.92 to 1.12)
10+ years	334; 1245	13.0; 13.2	1.21 (1.07 to 1.37)[α]	n/d	n/d	n/d
OESTROGEN COMBINED with						
**any progestogen**						
<1 year	1023; 4064	0.5; 0.5	1.09 (1.01 to 1.17)	2691; 12409	0.5; 0.5	0.98 (0.94 to 1.03)
1-2 years	1444; 5003	2.0; 1.9	1.27 (1.19 to 1.35)[β]	2302; 9617	1.9; 1.9	1.08 (1.03 to 1.14)[α]

3-4 years	1307; 4164	4.0; 4.0	1.42 (1.33 to 1.51)$^{\beta}$	1517; 6203	4.0; 3.9	1.10 (1.04 to 1.17)$^{\alpha}$
5-9 years	2863; 8004	7.4; 7.3	1.65 (1.57 to 1.73)$^{\beta}$	2111; 8212	7.0; 7.0	1.15 (1.09 to 1.21)$^{\beta}$
10+ years	1552; 3394	13.0; 12.9	2.07 (1.94 to 2.20)$^{\beta}$	432; 1512	11.6; 11.8	1.31 (1.17 to 1.46)$^{\beta}$
**Medroxyprogesterone**						
<1 year	624; 1924	0.5; 0.4	1.18 (1.07 to 1.29)$^{\beta}$	1559; 6245	0.4; 0.4	0.99 (0.94 to 1.06)
1-2 years	570; 1620	1.9; 1.9	1.33 (1.20 to 1.47)$^{\beta}$	835; 3022	1.9; 1.9	1.10 (1.01 to 1.19)
3-4 years	406; 1065	3.9; 4.0	1.54 (1.37 to 1.73)$^{\beta}$	404; 1439	3.9; 3.9	1.16 (1.04 to 1.31)$^{\alpha}$
5-9 years	447; 1101	7.0; 6.7	1.73 (1.54 to 1.94)$^{\beta}$	244; 960	6.4; 6.4	1.07 (0.92 to 1.23)
10+ years	107; 174	12.0; 12.2	2.60 (2.02 to 3.36)$^{\beta}$	n/d	n/d	n/d
**Levonorgestrel**						
<1 year	541; 1767	0.4; 0.5	1.15 (1.04 to 1.28)$^{\alpha}$	2840; 11725	0.4; 0.4	0.99 (0.94 to 1.03)
1-2 years	532; 1554	2.0; 1.9	1.31 (1.18 to 1.45)$^{\beta}$	1897; 7237	1.9; 1.9	1.04 (0.98 to 1.10)
3-4 years	413; 1203	4.0; 4.0	1.40 (1.25 to 1.57)$^{\beta}$	1044; 3702	3.9; 3.9	1.11 (1.03 to 1.19)$^{\alpha}$
5-9 years	731; 1826	7.2; 7.2	1.71 (1.56 to 1.87)$^{\beta}$	1235; 4006	7.3; 7.2	1.23 (1.15 to 1.31)$^{\beta}$
10+ years	245; 500	12.4; 12.5	2.20 (1.88 to 2.57)$^{\beta}$	n/d	n/d	n/d
**Norethisterone**						
<1 year	1068; 4133	0.5; 0.4	0.97 (0.90 to 1.04)	2998; 12348	0.4; 0.4	1.01 (0.97 to 1.06)
1-2 years	1047; 3404	1.9; 1.9	1.22 (1.13 to 1.31)$^{\beta}$	1591; 6303	1.8; 1.8	1.05 (0.99 to 1.12)
3-4 years	742; 2218	4.0; 3.9	1.43 (1.31 to 1.56)$^{\beta}$	799; 3025	3.9; 3.9	1.12 (1.03 to 1.21)$^{\alpha}$
5-9 years	1136; 2752	7.2; 7.0	1.81 (1.68 to 1.95)$^{\beta}$	757; 2753	7.0; 7.0	1.18 (1.09 to 1.29)$^{\beta}$
10+ years	351; 685	12.6; 12.3	2.21 (1.93 to 2.52)$^{\beta}$	n/d	n/d	n/d
**Dydrogesterone**						
<1 year	287; 934	0.4; 0.4	1.10 (0.96 to 1.26)	761; 3023	0.4; 0.4	0.93 (0.85 to 1.01)
1-2 years	172; 688	1.9; 1.8	0.95 (0.80 to 1.12)	318; 1250	1.8; 1.8	0.94 (0.83 to 1.07)
3-4 years	83; 302	4.0; 3.9	1.11 (0.86 to 1.42)	91; 444	3.7; 3.8	0.78 (0.62 to 0.98)
5-9 years	95; 338	6.9; 6.8	1.15 (0.91 to 1.45)	63; 227	6.7; 6.9	1.12 (0.84 to 1.51)
10+ years	25; 63	11.4; 11.7	1.54 (0.96 to 2.47)	n/d	n/d	n/d
TIBOLONE						

<1 year	367; 1407	0.4; 0.4	0.92 (0.82 to 1.04)	1206; 4997	0.4; 0.4	0.98 (0.91 to 1.04)
1-2 years	255; 858	1.9; 1.9	1.13 (0.98 to 1.30)	407; 1680	1.8; 1.8	0.99 (0.88 to 1.10)
3-4 years	166; 582	4.0; 3.9	1.19 (1.00 to 1.42)	168; 695	3.9; 3.9	1.00 (0.84 to 1.19)
5-9 years	250; 837	7.2; 7.1	1.28 (1.10 to 1.47)[β]	149; 650	7.2; 7.4	1.02 (0.85 to 1.22)
10+ years	76; 233	12.1; 12.7	1.44 (1.11 to 1.88)[α]	n/d	n/d	n/d

[#]Odds ratios are with reference to never-users and based on cases and controls matched by age and practice and adjusted for smoking status, body mass index, family history of cancer, medical conditions and events, other medications and contraceptive drugs; [α] P-value<0.01; [β] P-value<0.001; n/d – not enough data, few observations added to previous category

eTable 14 ***All cases and controls aged 55 and older:*** *Duration of use for different hormones of HRT and tibolone in women with* ***recent*** *(**between >1 and <5 years** before the index date) and **past** (**5 years or more** before the index date)exposures, combined analysis*

	**Recent exposure (between >1 and <5 years)**			**Past exposures (5 years or more)**		
Total number of women: 79951 cases and 371889 controls	N of cases; controls	Mean duration: cases; controls	Combined odds ratio[#] (95% confidence interval)	N of cases; controls	Mean duration: cases; controls	Combined odds ratio[#] (95% confidence interval)
OESTROGEN ONLY						
<1 year	415; 1885	0.5; 0.5	1.12 (1.00 to 1.25)	1156; 5458	0.4; 0.4	1.07 (1.00 to 1.15)
1-4 years	1464; 6580	3.0; 3.0	1.17 (1.10 to 1.24)[β]	1446; 7392	2.7; 2.7	0.99 (0.94 to 1.06)
5+ years	2223; 10208	9.3; 9.2	1.17 (1.11 to 1.23)[β]	1153; 5924	8.1; 8.1	1.00 (0.93 to 1.07)
**Conjugated equine oestrogen**						
<1 year	187; 882	0.5; 0.5	1.03 (0.87 to 1.21)	946; 4796	0.4; 0.4	0.99 (0.92 to 1.06)
1-4 years	630; 2872	3.1; 3.0	1.11 (1.02 to 1.22)	881; 4337	2.6; 2.6	1.00 (0.93 to 1.08)
5+ years	946; 4443	8.9; 9.1	1.12 (1.04 to 1.21)[α]	559; 2938	8.0; 8.0	0.96 (0.87 to 1.05)
**Estradiol**						
<1 year	406; 1864	0.4; 0.4	1.07 (0.96 to 1.20)	1003; 5088	0.4; 0.4	0.97 (0.90 to 1.04)
1-4 years	994; 4562	2.9; 2.9	1.11 (1.03 to 1.19)[α]	1046; 5316	2.6; 2.6	0.98 (0.92 to 1.05)
5+ years	1170; 5183	9.2; 9.0	1.18 (1.10 to 1.26)[β]	587; 2923	7.8; 7.8	1.00 (0.91 to 1.10)
OESTROGEN COMBINED with						
**any progestogen**						
<1 year	1059; 4569	0.5; 0.5	1.23 (1.14 to 1.32)[β]	3244; 16846	0.5; 0.5	0.98 (0.94 to 1.02)
1-4 years	4102; 14319	3.1; 3.0	1.52 (1.47 to 1.58)[β]	4489; 20994	2.7; 2.7	1.07 (1.04 to 1.11)[β]
5+ years	5936; 16913	9.0; 8.6	1.88 (1.82 to 1.94)[β]	2832; 12163	7.8; 7.7	1.17 (1.12 to 1.23)[β]
**Medroxyprogesterone**						
<1 year	879; 3097	0.5; 0.4	1.12 (1.04 to 1.22)[α]	1833; 8267	0.4; 0.4	0.98 (0.93 to 1.04)
1-4 years	1607; 4895	2.7; 2.7	1.45 (1.36 to 1.54)[β]	1403; 5824	2.5; 2.5	1.08 (1.01 to 1.15)
5+ years	724; 1816	7.7; 7.3	1.91 (1.75 to 2.10)[β]	273; 1192	6.4; 6.4	1.07 (0.94 to 1.23)
**Levonorgestrel**						

<1 year	543; 2000	0.5; 0.5	1.15 (1.04 to 1.27)[α]	3427; 15869	0.4; 0.4	0.98 (0.94 to 1.02)
1-4 years	1390; 4347	3.0; 2.9	1.47 (1.38 to 1.57)[β]	3612; 14685	2.6; 2.5	1.08 (1.03 to 1.12)[β]
5+ years	1368; 3635	8.2; 8.0	1.86 (1.74 to 1.99)[β]	1397; 4996	7.2; 7.2	1.24 (1.16 to 1.32)[β]
**Norethisterone**						
<1 year	1320; 5027	0.5; 0.5	1.14 (1.07 to 1.22)[β]	3533; 16186	0.4; 0.4	1.02 (0.98 to 1.06)
1-4 years	2612; 8741	2.9; 2.8	1.42 (1.35 to 1.49)[β]	2790; 12357	2.5; 2.5	1.06 (1.01 to 1.11)
5+ years	1979; 5039	8.1; 7.8	2.00 (1.89 to 2.11)[β]	831; 3496	7.0; 7.0	1.15 (1.06 to 1.24)[β]
**Dydrogesterone**						
<1 year	303; 1022	0.4; 0.4	1.11 (0.97 to 1.27)	883; 3803	0.4; 0.4	0.96 (0.89 to 1.04)
1-4 years	307; 1316	2.7; 2.6	0.98 (0.86 to 1.12)	467; 2149	2.2; 2.3	0.92 (0.83 to 1.02)
5+ years	137; 493	7.8; 7.6	1.28 (1.06 to 1.56)	67; 288	6.7; 6.8	1.08 (0.82 to 1.42)
TIBOLONE						
<1 year	536; 2263	0.4; 0.4	0.94 (0.86 to 1.04)	1480; 6710	0.4; 0.4	0.99 (0.93 to 1.05)
1-4 years	718; 2777	2.7; 2.7	1.15 (1.05 to 1.25)[α]	706; 3230	2.4; 2.4	0.99 (0.91 to 1.07)
5+ years	448; 1630	8.1; 8.0	1.34 (1.21 to 1.49)[β]	172; 821	7.2; 7.3	1.02 (0.86 to 1.20)

[#]Odds ratios are with reference to never-users and based on cases and controls matched by age and practice and adjusted for smoking status, body mass index, family history of cancer, medical conditions and events, other medications and contraceptive drugs; [α] P-value<0.01; [β] P-value<0.001; n/d – not enough data, few observations added to previous category

eTable 15 ***All cases and controls:*** *Years of use since the first prescription, for different hormones of HRT, by database*

	QResearch		CPRD		Combined analysis	
	N of cases; controls	Adjusted odds ratio[#] (95% confidence interval)	N of cases; controls	Adjusted odds ratio[#] (95% confidence interval)	Combined odds ratio (95% confidence interval)	P-value
**HRT**						
<1 year	3274; 15577	1.03 (0.99 to 1.07)	2469; 12221	1.07 (1.02 to 1.12)[α]	1.05 (1.02 to 1.08)	0.003
1-2 years	3437; 15200	1.11 (1.06 to 1.15)[β]	2390; 10411	1.21 (1.15 to 1.27)[β]	1.15 (1.11 to 1.18)	<0.001
3-4 years	3400; 14103	1.20 (1.15 to 1.25)[β]	2064; 9150	1.21 (1.15 to 1.27)[β]	1.20 (1.16 to 1.24)	<0.001
5-9 years	6866; 26746	1.30 (1.26 to 1.35)[β]	4030; 17502	1.28 (1.23 to 1.33)[β]	1.29 (1.26 to 1.33)	<0.001
10+ years	3517; 11950	1.53 (1.47 to 1.60)[β]	2256; 9531	1.37 (1.30 to 1.44)[β]	1.47 (1.42 to 1.52)	<0.001
OESTROGEN ONLY						
<1 year	768; 3497	1.10 (1.01 to 1.19)	668; 3194	1.09 (0.99 to 1.19)	1.09 (1.03 to 1.16)	0.004
1-2 years	741; 3705	1.01 (0.93 to 1.09)	600; 2825	1.08 (0.99 to 1.19)	1.04 (0.98 to 1.11)	0.2
3-4 years	826; 3848	1.09 (1.01 to 1.18)	553; 2726	1.06 (0.96 to 1.17)	1.08 (1.02 to 1.15)	0.01
5-9 years	1772; 8499	1.08 (1.02 to 1.14)	1181; 5947	1.08 (1.01 to 1.16)	1.08 (1.03 to 1.13)	<0.001
10+ years	1019; 4753	1.13 (1.05 to 1.21)[α]	732; 3805	1.11 (1.01 to 1.21)	1.12 (1.06 to 1.18)	<0.001
**Conjugated equine oestrogen**						
<1 year	597; 3045	0.95 (0.87 to 1.05)	491; 2606	0.97 (0.87 to 1.07)	0.96 (0.90 to 1.03)	0.2
1-2 years	446; 2363	0.93 (0.83 to 1.03)	347; 1713	1.02 (0.91 to 1.16)	0.97 (0.89 to 1.05)	0.4
3-4 years	436; 1935	1.11 (1.00 to 1.24)	278; 1329	1.08 (0.95 to 1.24)	1.10 (1.01 to 1.20)	0.03
5-9 years	798; 3965	1.01 (0.94 to 1.10)	549; 2558	1.13 (1.02 to 1.25)	1.06 (1.00 to 1.13)	0.07
10+ years	403; 1943	1.06 (0.95 to 1.18)	263; 1416	1.04 (0.90 to 1.19)	1.05 (0.96 to 1.14)	0.3
**Estradiol**						
<1 year	685; 3400	0.99 (0.91 to 1.08)	628; 3146	1.01 (0.92 to 1.11)	1.00 (0.94 to 1.06)	0.9
1-2 years	595; 2903	1.01 (0.92 to 1.10)	493; 2270	1.09 (0.98 to 1.21)	1.04 (0.97 to 1.12)	0.2
3-4 years	535; 2551	1.05 (0.95 to 1.15)	367; 1963	0.95 (0.85 to 1.07)	1.01 (0.93 to 1.08)	0.9
5-9 years	1008; 4801	1.05 (0.98 to 1.13)	711; 3591	1.05 (0.96 to 1.14)	1.05 (0.99 to 1.11)	0.09
10+ years	550; 2321	1.21 (1.10 to 1.34)[β]	377; 1981	1.06 (0.94 to 1.19)	1.15 (1.07 to 1.24)	<0.001

OESTROGEN COMBINED with						
**any progestogen**						
<1 year	2510; 12109	1.01 (0.96 to 1.06)	1801; 9027	1.06 (1.00 to 1.12)	1.03 (0.99 to 1.07)	0.1
1-2 years	2696; 11501	1.13 (1.08 to 1.18)[β]	1790; 7586	1.25 (1.18 to 1.32)[β]	1.18 (1.14 to 1.22)	<0.001
3-4 years	2574; 10258	1.23 (1.17 to 1.29)[β]	1511; 6424	1.26 (1.18 to 1.33)[β]	1.24 (1.19 to 1.28)	<0.001
5-9 years	5096; 18248	1.39 (1.34 to 1.44)[β]	2849; 11555	1.36 (1.29 to 1.42)[β]	1.38 (1.34 to 1.42)	<0.001
10+ years	2498; 7198	1.76 (1.68 to 1.85)[β]	1524; 5726	1.50 (1.41 to 1.59)[β]	1.65 (1.59 to 1.72)	<0.001
**Medroxyprogesterone**						
<1 year	1748; 7152	1.02 (0.96 to 1.08)	1051; 4586	1.05 (0.98 to 1.13)	1.03 (0.99 to 1.08)	0.2
1-2 years	1190; 4482	1.10 (1.03 to 1.18)[α]	708; 2662	1.23 (1.13 to 1.35)[β]	1.15 (1.09 to 1.21)	<0.001
3-4 years	810; 2700	1.28 (1.18 to 1.38)[β]	435; 1626	1.25 (1.12 to 1.39)[β]	1.27 (1.19 to 1.35)	<0.001
5-9 years	758; 2501	1.30 (1.20 to 1.42)[β]	395; 1451	1.30 (1.16 to 1.46)[β]	1.30 (1.22 to 1.39)	<0.001
10+ years	184; 318	2.39 (1.98 to 2.89)[β]	57; 205	1.31 (0.97 to 1.77)	2.02 (1.72 to 2.37)	<0.001
**Levonorgestrel**						
<1 year	2443; 10414	1.03 (0.98 to 1.08)	1607; 7599	1.02 (0.96 to 1.08)	1.02 (0.99 to 1.06)	0.2
1-2 years	2072; 7827	1.15 (1.09 to 1.21)[β]	1104; 4881	1.07 (1.00 to 1.15)	1.12 (1.08 to 1.17)	<0.001
3-4 years	1472; 5507	1.16 (1.10 to 1.24)[β]	849; 3248	1.26 (1.16 to 1.36)[β]	1.20 (1.14 to 1.26)	<0.001
5-9 years	2003; 6307	1.41 (1.34 to 1.49)[β]	1102; 4294	1.26 (1.17 to 1.35)[β]	1.35 (1.29 to 1.41)	<0.001
10+ years	484; 1349	1.60 (1.43 to 1.78)[β]	304; 1027	1.49 (1.30 to 1.70)[β]	1.55 (1.43 to 1.69)	<0.001
**Norethisterone**						
<1 year	2805; 12073	1.02 (0.97 to 1.06)	1993; 9319	1.04 (0.99 to 1.10)	1.03 (0.99 to 1.06)	0.1
1-2 years	2034; 8278	1.09 (1.04 to 1.15)[β]	1330; 5369	1.21 (1.14 to 1.29)[β]	1.14 (1.09 to 1.19)	<0.001
3-4 years	1490; 5518	1.21 (1.14 to 1.29)[β]	922; 3686	1.25 (1.16 to 1.35)[β]	1.23 (1.17 to 1.29)	<0.001
5-9 years	2153; 7378	1.33 (1.26 to 1.40)[β]	1110; 4416	1.28 (1.20 to 1.38)[β]	1.31 (1.26 to 1.37)	<0.001
10+ years	669; 1644	1.84 (1.68 to 2.02)[β]	328; 1141	1.47 (1.29 to 1.67)[β]	1.70 (1.58 to 1.83)	<0.001
**Dydrogesterone**						
<1 year	850; 3401	0.97 (0.90 to 1.05)	473; 2156	0.96 (0.86 to 1.06)	0.97 (0.91 to 1.03)	0.3
1-2 years	432; 1778	0.96 (0.86 to 1.07)	215; 1110	0.86 (0.74 to 1.00)	0.92 (0.85 to 1.01)	0.08
3-4 years	209; 962	0.86 (0.74 to 1.00)	102; 503	0.94 (0.76 to 1.17)	0.88 (0.78 to 1.00)	0.05

5-9 years	196; 740	1.07 (0.91 to 1.26)	77; 404	0.91 (0.71 to 1.17)	1.02 (0.89 to 1.17)	0.8
10+ years	42; 121	1.26 (0.88 to 1.82)	18; 71	1.15 (0.68 to 1.95)	1.23 (0.91 to 1.65)	0.2
TIBOLONE						
<1 year	1166; 4910	0.98 (0.91 to 1.04)	837; 4064	0.93 (0.86 to 1.01)	0.96 (0.91 to 1.01)	0.1
1-2 years	558; 2356	0.98 (0.90 to 1.08)	390; 1753	0.99 (0.88 to 1.10)	0.99 (0.92 to 1.06)	0.7
3-4 years	288; 1368	0.90 (0.79 to 1.03)	227; 904	1.16 (1.00 to 1.35)	1.01 (0.91 to 1.11)	0.9
5-9 years	386; 1540	1.09 (0.97 to 1.22)	270; 1153	1.12 (0.98 to 1.28)	1.10 (1.01 to 1.20)	0.03
10+ years	117; 388	1.37 (1.11 to 1.70)[α]	69; 291	1.16 (0.89 to 1.51)	1.29 (1.09 to 1.52)	0.003

[#]Odds ratios are with reference to never-users and based on cases and controls matched by age and practice and adjusted for smoking status, body mass index, family history of cancer, medical conditions and events, other medications and contraceptive drugs.

[α] P-value<0.01; [β] P-value<0.001

eTable 16 Comparison our results with the recent meta-analysis (Lancet 2019)

Our study<br>Duration for recent users (between >1 and <5 years) in women aged 55 and older				Meta-analysis (Lancet 2019)<br>Duration for current users (within 5 years)			
	Number of cases	Mean use in years in cases	Odds Ratio (95% CI)		Number of cases	Mean use in years in cases	Relative Risk (95% CI)
**Oestrogen only**				**Oestrogen only**			
<1 year	415	0.5	1.08 (0.90-1.29)	<1 year	109	<1.0	1.08 (0.86-1.35)
1 -4 years	1464	3.0	1.27 (1.15-1.40)	1-4 years	1150	2.8	1.17 (1.10-1.26)
5 -9 years	1469	7.3	1.16 (1.09-1.23)	5-9 years	2391	7.0	1.22 (1.17-1.28)
10+	754	13.1	1.20 (1.11-1.31)	10-14 years	2478	11.7	1.43 (1.37-1.50)
				15+	2183	20.1	1.58 (1.51-1.66)
**Oestrogen and progestogen**				**Oestrogen and progestogen**			
<1 year	1059	0.5	1.13 (0.87-1.47)	<1 year	189	<1.0	1.20 (1.01-1.43)
1-4 years	4102	3.1	1.37 (1.14-1.64)	1-4 years	2230	2.8	1.60 (1.52-1.69)
5-9 years	4124	7.2	1.78 (1.72-1.86)	5-9 years	4968	7.0	1.97 (1.90-2.04)
10+	1812	12.9	2.14 (2.02-2.27)	10-14 years	3350	11.7	2.26 (2.16-2.36)
				15+	1424	20.1	2.51 (2.35-2.68)
**Main types of hormones 5+ years of recent use**				**Main types of hormones 5-14 years of current use**			
Equine oestrogen	946	8.9	1.12 (1.04-1.21)	Equine oestrogen	1910	9	1.32 (1.25-1.39)
Estradiol	1170	9.2	1.18 (1.10-1.26)	Estradiol	1563	9	1.38 (1.30-1.46)
Levonorgestrel	1368	8.2	1.86 (1.74-1.99)	Levonorgestrel	1735	9	2.12 (1.99-2.25)
Norethisterone	1979	8.1	2.00 (1.89-2.11)	Norethisterone	2642	9	2.20 (2.09-2.32)
Medroxyprogesterone	724	7.7	1.91 (1.75-2.10)	Medroxyprogesterone	2012	9	2.07 (1.96-2.19)
Dydrogesterone	137	7.8	1.28 (1.06-1.56)	Dydrogesterone	162	9	1.41 (1.17-1.71)
Tibolone	448	8.1	1.34 (1.21-1.49)	Tibolone	680	9	1.57 (1.43-1.72)
